# Na@SiO_2_-Mediated Addition of Organohalides to Carbonyl Compounds for the Formation of Alcohols and Epoxides

**DOI:** 10.1038/srep36225

**Published:** 2016-11-17

**Authors:** Mohit Kapoor, Jih Ru Hwu

**Affiliations:** 1 Department of Chemistry, National Tsing Hua University, Hsinchu 300, Taiwan; 2 Frontier Research Center on Fundamental and Applied Sciences of Matters, National Tsing Hua University, Hsinchu 300, Taiwan

## Abstract

Alcohols and epoxides were generated by the addition of organohalides to carbonyl compounds in the presence of sodium metal impregnated with silica gel (Na@SiO_2_) in THF at 25 °C through a radical pathway. Under the same conditions, Schiff bases were also successfully converted to the corresponding amines. Furthermore, the reaction of aldehydes with α-haloesters or 4-(chloromethyl)-coumarin with the aid of Na@SiO_2_ generated trans epoxides. An unprecedented mechanism is proposed for their formation. The advantages associated with these new reactions include: (1) products are obtained in good-to-excellent yields, (2) reactions are completed at room temperatures in a short period of time (<2.0 h), (3) it is unnecessary to perform the reactions under anhydrous conditions, and (4) the entire process requires only simple manipulations.

Carbon–carbon bond formation is the essence of organic synthesis. The development of general strategies for the formation of carbon–carbon and carbon–heteroatom bonds is of great interest to scientists working in the fields of medicinal chemistry, agrochemical production, and natural product synthesis. Common methods to elongate the molecular skeleton involve additions of organometallic reagents to carbonyl compounds^1^. Among these methods, the most notable reactions involve the use of Grignard or alkyllithium reagents to react with ketones for the generation of the corresponding alcohols^2^,^3^,^4^. Nevertheless, competitive reduction products and aldol adducts are often produced^5^,^6^. Improved results for the alkylation of carbonyl compounds with Grignard reagents can be obtained when metal salts are added^1^,^5^, including CeCl_3_, LiCl, LnCl_3_·2LiCl, and ZnCl_2_, among others. Li *et al.* reported the environmentally friendly Barbier–Grignard type alkylation and arylation of carbonyl compounds in water using metal catalysts such as Rh(acac)(CO)_2_/Zn^7^, Zn/CuI^8^, and RuCl_3_/In(OAc)_3_^9^. Capriati *et al.*^10^,^11^,^12^ applied the Barbier–Grignard type reactions to the synthesis of substituted tetrahydrofurans from γ-haloketones. The same research group explored the lithium-induced alkylative ring opening of tetrahydrofurans, which leads to the synthesis of primary alcohols^12^,^13^.

Several reliable reactions exist for the preparation of epoxides, which are versatile intermediates in organic synthesis^14^,^15^. Often alkenes and carbonyl compounds serve as the starting materials. A different approach to obtain epoxides with an α-ester functionality (i.e., glycidic ester) is through the Darzens reaction^16^, in which an α-haloester reacts with an aldehyde or a ketone in the presence of a base. Thus C–C and C–O bond formations take place in sequence to give an α, β-epoxy ester. This reaction has a limited scope due to it being a time-consuming process with relative low yields of the products^17^. Recently, a phase-transfer catalyzed Darzens reaction has been reported^18^. Technical problems associated with this reaction include the requirement of separation of the catalysts from the reaction mixture and their subsequent reuse or disposal. This gives rise to negative environmental impacts. Accordingly, it is in need of a new method that can overcome these problems.

Alkali metals on inert supports^19^ or as solutions in amines^20^ have been widely applied for reductions. Notable examples include the Benkeser reaction, the Birch reduction^21^, the calcium reduction^22^,^23^,^24^, and the Wurtz reaction^20^. Silica gel is an ideal support for heterogeneous catalysts due to its excellent thermal and chemical stability. Its use is also cost-effective. Recently, solid alkali metal-based reductions in organic solvents have become easier than before with the finding that these metals can be made significantly less pyrophoric by thermal absorption into nanostructured silica to form the metal–silica gel reagents^19^. Such an approach provides a green chemistry pathway by reducing the dangers associated with the handling of naked alkali metals^19^. Recently, applications of alkali metals and their salts (Na, Na_2_K, and K_2_Na) in silica gel are demonstrated in the Birch reduction^21^, desulfurization^19^, desulfonation^25^, and ester reduction^26^.

By considering the advantages associated with the metal-silica gel reagents, we developed new C–C bond formation methods involving the use of sodium metal impregnated with silica gel (i.e., Na@SiO_2_). This free-flowing powder can be handled easily in an open atmosphere. Its applications enabled the effective alkylation and arylation of carbonyl compounds **1** with organohalides **2** to give alcohols **3** under mild conditions (Fig. 1). It also assisted the epoxide formation by the reaction of α-haloesters **4** with aldehydes or ketones **1** with high efficiency. To the best of our knowledge, these are the first Na@SiO_2_-mediated syntheses of alcohols and epoxides.

## Results

### Reaction Optimization and Scope

Our initial attempts included the reaction of cyclopropyl phenyl ketone (**1f**, 1.0 equiv) with 4-bromotoluene (**2b**, 1.2 equiv) in the presence of Na@SiO_2_ (10 equiv) in a mixture of THF and methanol (1:1, Fig. 2). The desired alkylated product **3f** was obtained in 19% yield along with the by-product alcohol **6** (28%) through reduction at 25 °C for 2.0 h. The major product was the self-coupled biphenyl **7** (47% yield). The reaction was found to be dependent chiefly on the equivalent of Na@SiO_2_ used and whether the solvent was aprotic. Increased amount of the desired adduct **3f** to 48% yield was obtained when 5.0 equivalents of Na@SiO_2_ were used in THF without any co-solvent. The best result was obtained when the reaction was carried out in the presence of 1.4 equivalents of Na@SiO_2_ in THF at 25 °C for 2.0 h. To our satisfaction, the desired tertiary alcohol **3f** was produced in 89% yield. This newly developed reaction was found tolerable with moisture as shown by the results in the last row of Fig. 2. In a control experiment, arylation of phenone **1f** was performed by addition of ~2.0% of water in THF. The product distribution was found almost the same as that with dry THF.

With the optimized conditions in hand, the scope and applicability of this new C–C bond formation was explored. Different ketones and aldehydes were found to be adaptable to the new reaction, which included alkanones **1a** and **1b**, four- to six-membered cycloalkanones **1c**–**e** (entries 1–5 of Table 1), and phenones **1f**–**h** (entries 6–8). Aryl aldehydes **1i** and **1j** (entries 9 and 10) were also transformed in success to the corresponding secondary alcohols **3i** and **3j**, respectively. Naturally-occurring steroid pregnenolone **1k** (entry 11) underwent smooth alkylation with *n*-butyl bromide **2d** to afford the corresponding alcohol **3k** in 78% yield. Furthermore, treatment of the *N*-protected alkaloid, indole-3-carboxyaldehyde **1l** (entry 12), with allyl bromide **2e** in presence of Na@SiO_2_ produced the homoallylic alcohol **3l** in 80% yield through a 1,2-addition pathway.

Schiff bases are often inert toward alkylation under mild conditions^27^. By utilization of the Na@SiO_2_ reagent, Schiff bases **8a** and **8b** reacted with allyl bromide (**2e**) or *tert*-butyl bromide (**2c**) to give the amines **9a**–**c** in very good yields (87–90%, entries 13–15 of Table 1). In contrast, when the sterically hindered *tert*-butyl bromide is employed in the Grignard reaction, the adducts are usually generated in moderate yields^28^.

**Figure 1:**
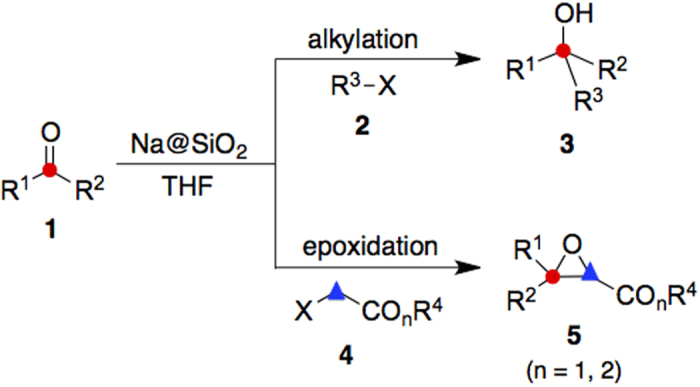
Application of Na@SiO_2_ in the C–C and C–O bond formations. Carbonyl compound **1** underwent C–C and C–O bond formations with organohalides **2** and α-haloesters **4** to give alcohols **3** and epoxides **5**, respectively.

The C–C bond formation methods shown in Figs 1 and 3 were adaptable to various organohalides. They included aryl chloride **2a**, aryl bromide **2b**, sterically hindered bromide **2c**, alkyl bromide **2d**, and allyl bromide **2e**. The corresponding alcohols and amines were obtained in good to excellent yields (77–91%, Table 1).

### Epoxide Formation and Antiviral Activity

Encouraged by the success on the Na@SiO_2_-mediated formation of alcohols and amines, we expanded its applicability to the Darzens reaction for epoxide synthesis. Treatment of an α-halo ester **4a**, lactone **4b,** or ketone **4c** with aldehydes **1i–o** (entries 1–5 of Table 2) in the presence of Na@SiO_2_ (1.8–2.0 equiv) in THF provided glycidic esters **5i–q** in 80–90% yields in 30 min. The moieties attached to the glycidic esters could be substituted phenyl, naphthyl, and benzodioxole groups. In comparison with the results obtained from the standard conditions associated with the Darzens reaction, the yields are much higher (80–91% versus 19–82%) and the reaction time is shorter (30 min versus 16–70 h)^29^,^30^ for the newly developed method shown in Fig. 1.

During the epoxide formation, the ester moiety in compounds **4** (see Fig. 1) could be replaced by an α, β-unsaturated lactone moiety. Efficient formation of epoxides **11a–c** was accomplished by reaction of aldehydes **1j** and **1n** with chlorocoumarins **10a–c** (1.2 equiv) and Na@SiO_2_ (1.8–2.0 equiv) at room temperature (see entries 10–12 of Table 2).

Compounds containing a coumarin moiety in connection with benzothiazole, benzoxazole, or imidazopyridine exhibit antiviral activity^31^,^32^,^33^,^34^. Thus the biological activity of coumarin epoxides **11a–c** (see entries 10–12 of Table 2) was evaluated in the hepatitis C virus (HCV) subgenomic replicon system in huh 5-2 cells. All of these three new compounds showed positive results. For the coumarin epoxide **11b**, its 50% inhibitory concentration for virus replication (EC_50_) and host cell growth (CC_50_) were 0.90 and 91.9 μM, respectively. The SI value (CC_50_/EC_50_) was as high as 102. To the best of our knowledge, this compound exhibited the most appealing anti-HCV activities in comparison with all others in the coumarin family.

## Discussion

**Figure 2:**
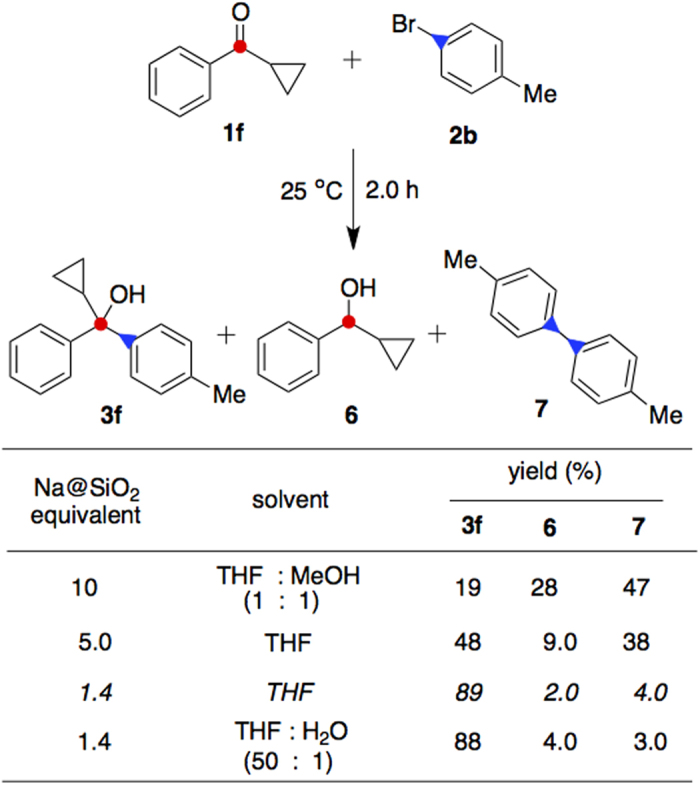
Optimization of the alkylation reaction. Reaction conditions were optimized by use of THF, methanol, and water as solvents with different molar equivalents of Na@SiO_2_. The best results were obtained in the presence of 1.4 equivalents of Na@SiO_2_ in THF.

**Figure 3:**
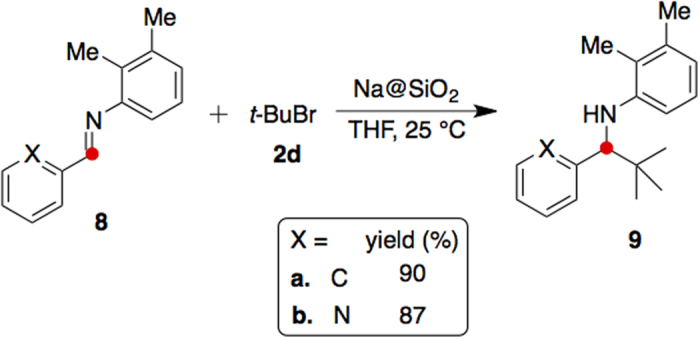
Alkylation of Schiff bases with a sterically hindered bromide. *tert*-Butyl bromide was found reactive for alkylation of the Schiff bases **8** in the presence of Na@SiO_2_.

In the Na@SiO_2_-mediated alkylation reactions, the self-coupled dimer **7** from *p*-bromotoluene was isolated as the by-product (Fig. 2)^35^. In a control experiment, this reaction was carried out in the presence of (2,2,6,6-tetramethyl-1-piperidinyl)oxy (TEMPO), which acted as a radical scavenger. Consequently, the desired adduct **3f** was not detected at all. The same radical quenching phenomenon was also observed in the examples for the formation of hindered amines as shown in Fig. 3. These results indicate a radical pathway involved when Na@SiO_2_ was employed to initiate the alkylation.

**Figure 4:**
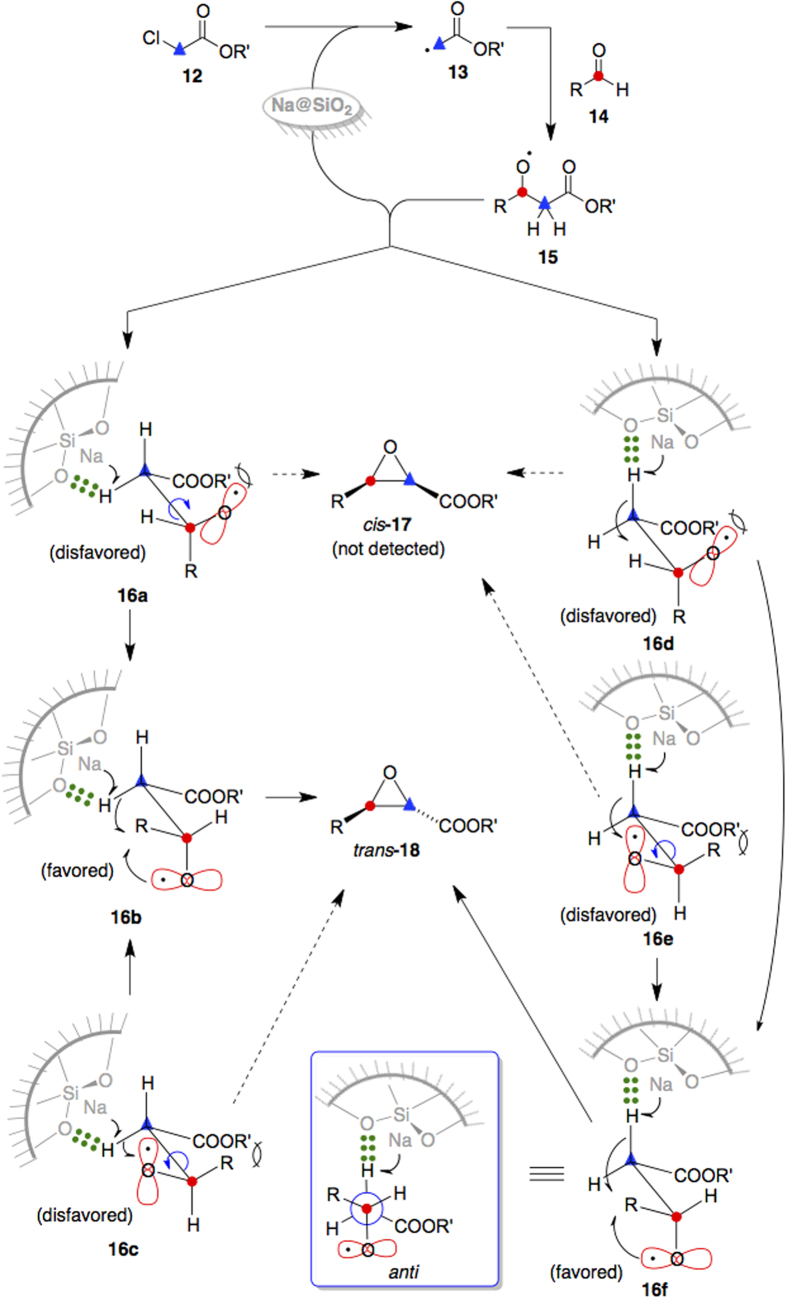
A plausible mechanistic for formation of glycidic esters from α-chloroacetates and carbonyl compounds in the presence of Na@SiO_2_. The epoxide products **18** were generated in the trans form; their formation originates from the thermodynamically more stable anti conformation between the R and the COOR’ groups in the conformers **16b** and **16f**.

A plausible mechanism for the glycidic ester formation is presented in Fig. 4. After Na@SiO_2_ removes the chlorine atom from α-chloroacetates **12**, the carboradicals **13** adds to aldehydes **14** to give the radical intermediates **15**. It has been reported that metal-silica gel reagents can adsorb substrates^36^. Adsorption of the alkoxyl radicals **15** to silica gel impregnated with sodium metal could bring the substrates and the electron donors into proximity^36^. Use of silica gel as support also increases the effective surface area and constrain both the substrates and the metals in pores for decreasing the entropy of activation for electron transfer^37^. As a consequence, at least six possible isomers **16a**–**f** can be generated as shown in Fig. 4. The isomers **16a** and **16c** with internal steric hindrance would transform to the thermodynamically more stable conformers **16b**; likewise, isomers **16d** and **16e** would transform to conformers **16f**. After a diastereotopic methylene hydrogen in the isomers **16b** or **16f** is removed by Na@SiO_2_, an epoxide formation reaction takes place to give primarily the trans epoxides **18** (not the cis epoxide **17**) with high diastereoselectivity. The trans configuration of the epoxide **18** originates from the thermodynamically more stable *anti* conformation between the R and the COOR’ groups in **16b** and **16f** (cf. the Newman projection of **16f**).

The mechanism shown in Fig. 4 indicates that abstraction of diastereotopic protons by Na@SiO_2_ triggers the formation of epoxides. Nevertheless, we did not observe the similar phenomenon for allyl bromide **2e**, which also has two diastereotopic protons. Allyl bromide **2e** underwent alkylation with carbonyl compound **1l** and imine **8a** to form secondary alcohol **3l** or amine **9c**, respectively, instead of an epoxide or aziridine. Therefore, the presence of an electron-withdrawing group (such as –COR and –COOR) in an organohalide is the borderline between the alcohol and epoxide formation.

The Darzens reaction is the anionic condensation of an α-haloester with an aldehyde in the presence of base to produce a glycidic ester. It goes through an intramolecular S_N_2 attack process to generate an α-chloro-β-oxide as the intermediate. In the newly developed epoxide formation reaction, the same starting materials were used and the base was replaced by Na@SiO_2_. α-Chloro-β-hydroxy esters were not detected in any reaction mixtures. Therefore, our glycidic ester formation reaction deviates from the conventional Darzens reaction with respect to their reaction mechanisms^16^.

In conclusion, Na@SiO_2_ was developed as a powerful reagent to solve one of the most important themes in organic synthesis — carbon to carbon bond formation. In the presence of Na@SiO_2_, the addition of organohalides to ketones and aldehydes gave the corresponding adducts (i.e., alcohols) in good to excellent yields. The Na@SiO_2_ reagent also assisted the reaction of α-halocarbonyl compounds with aldehydes in the formation of glycidic esters through an unprecedented radical pathway. In comparison with the myriad other available methods, the four advantages associated with the newly developed reactions include: (1) The desired adducts are often obtained in very good yields. (2) Reactions are completed at room temperature within 2.0 hours. (3) The requirement for anhydrous solvents is unnecessary. (4) The manipulation of the heterogeneous reactions is simple. Application of the environment-benign reagent Na@SiO_2_ in organic synthesis fits into five of the twelve guidelines of green chemistry^38^. Furthermore, a new coumarin-containing epoxide was generated by this newly developed method; it possessed high potency and selectivity against the hepatitis C virus.

## Methods

### The Standard Procedure 1 for the Syntheses of Alcohols and Amines

A reaction flask equipped with a magnetic stirring bar, rubber stopper, and nitrogen balloon was charged with Na@SiO_2_ (1.3–1.7 equiv). To this reaction mass was added THF (2.0–4.0 mL) via syringe at room temperature. Then an aldehyde, ketone, or imine (**1** or **8**, 1.0 equiv) was premixed with an organohalide (**2**, 1.2 equiv). The mixture was diluted with THF (0.50 mL) and the resultant solution was injected into the reaction mass via syringe. After the reaction mixture was stirred at 25 °C for 1.0–3.0 h, the inorganic residue was filtered. The filtrate was concentrated under reduced pressure and then purified by use of column chromatography packed with silica gel and eluented with a mixture of EtOAc and hexanes to give the desired alcohols **3** or amines **9**.

### The Standard Procedure 2 for the Synthesis of Epoxides

A reaction flask equipped with a magnetic stirring bar, rubber stopper, and nitrogen balloon was charged with Na@SiO_2_ (1.8–2.0 equiv). To this reaction mass was added THF (1.0–2.0 mL) via syringe at room temperature. Then an aldehyde **1** was premixed with an organohalide **4** or **10** (1.2 equiv). The mixture was diluted with THF (0.50 mL) and the resultant solution was injected into the reaction mass via syringe. After the reaction mixture was stirred at 25 °C for 0.50–2.0 h, the inorganic residue was filtered. The filtrate was concentrated under reduced pressure and then purified by use of column chromatography packed with silica gel and eluented with a mixture of EtOAc and hexanes to give the desired *trans*-epoxides **5** or **11**.

## Additional Information

**How to cite this article**: Kapoor, M. and Hwu, J. R. Na@SiO_2_-Mediated Addition of Organohalides to Carbonyl Compounds for the Formation of Alcohols and Epoxides. *Sci. Rep.* **6**, 36225; doi: 10.1038/srep36225 (2016).

**Publisher’s note**: Springer Nature remains neutral with regard to jurisdictional claims in published maps and institutional affiliations.

## Supplementary Material

Supplementary Information

## Figures and Tables

**Table 1:**
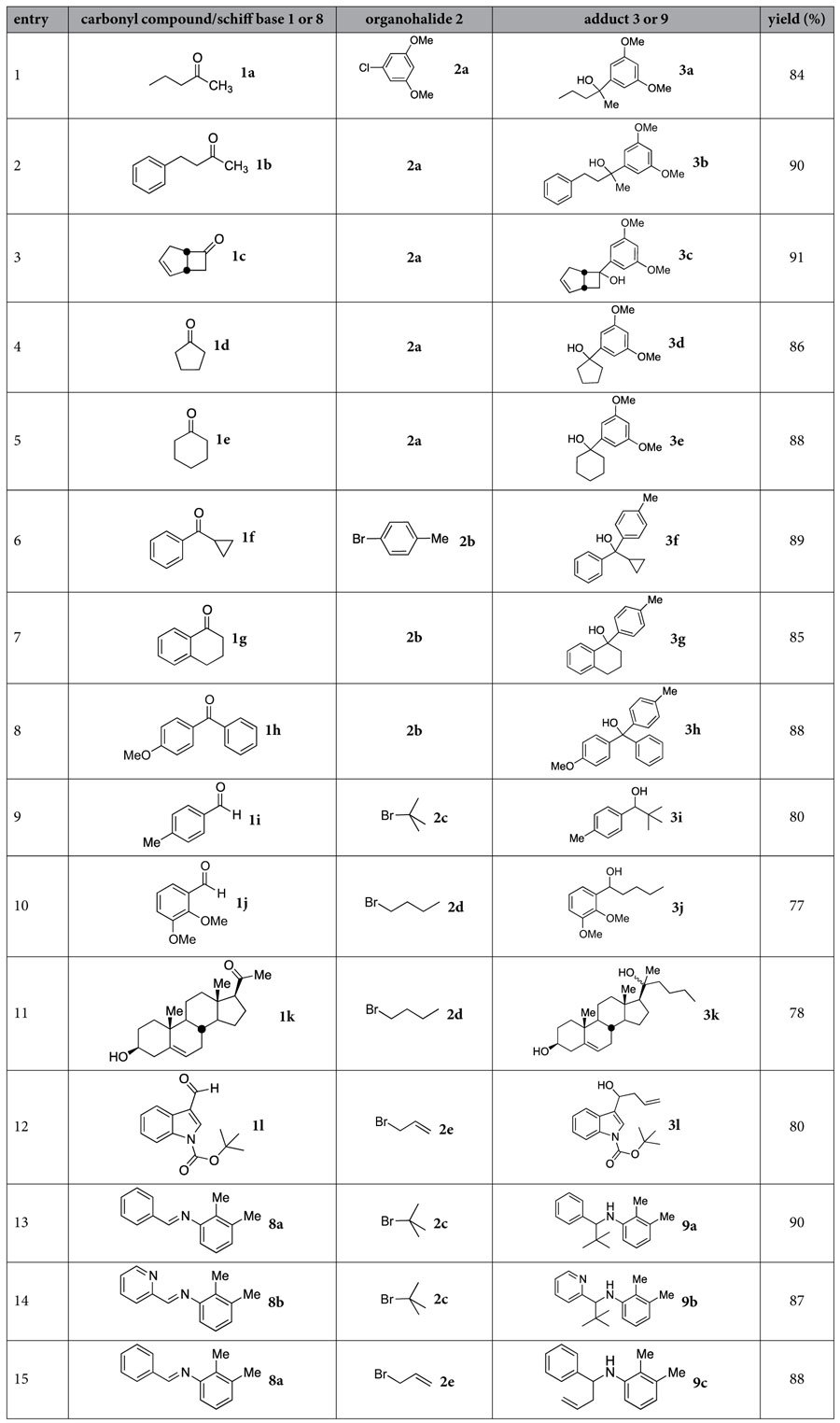
Reaction of ketones, aldehydes, and Schiff bases with organohalides in the presence of Na@SiO_2_ to give the corresponding adducts by formation of C–C bonds.

**Table 2:**
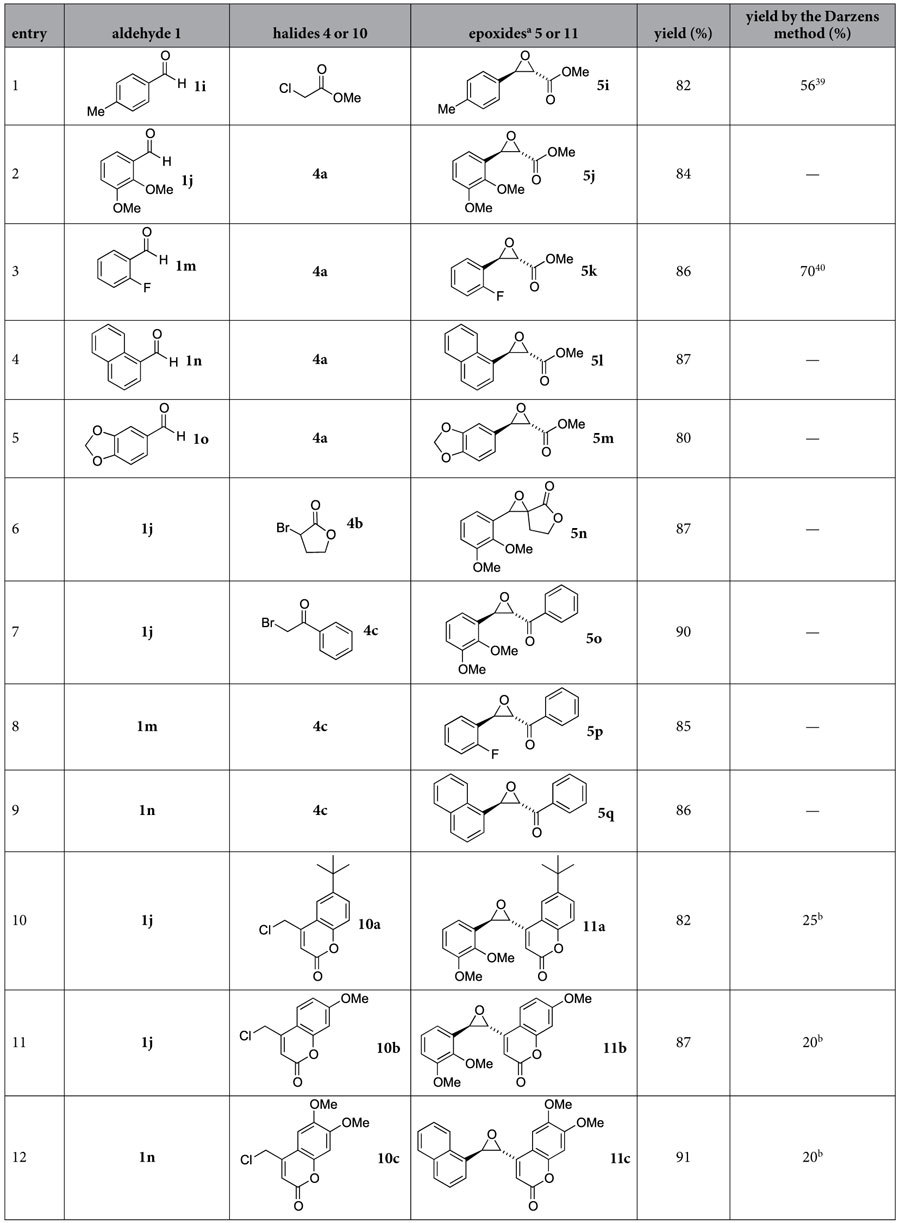
Epoxide formation by reaction of aldehydes with α-haloesters or 4-(chloromethyl)coumarins with the aid of Na@SiO_2_.

^a^Epoxides were obtained in the trans form exclusively.

^b^The maximum yields obtained in our laboratory for the Darzens reaction by using NaOH, NaOMe, or kO*t*Bu as the base.
