# Supplementary material for: Na@SiO2-Mediated Addition of Organohalides to Carbonyl Compounds for the Formation of Alcohols and Epoxides
Source: Sci Rep. 2016 Nov 17;6:36225. doi: 10.1038/srep36225 (PMC5113255; doi:10.1038/srep36225)
Supplement: Supplementary Information [file srep36225-s1.pdf]

Supplementary Information

Na@SiO_2-Mediated Addition of Organohalides to Carbonyl Compounds for the Formation of Alcohols and Epoxides

Mohit Kapoor[†,‡] *and Jih Ru Hwu*[†,‡,*]

[†]Department of Chemistry, National Tsing Hua University, Hsinchu 300, Taiwan

[‡]Frontier Research Center on Fundamental and Applied Sciences of Matters, National Tsing Hua University, Hsinchu 300, Taiwan

Email: jrhwu@mx.nthu.edu.tw

Contents

Content ……………………………………………………………………….. S1

Experimental ……………………………………………………………………. S2

Reference ……………………………………………………………………. S18

^{1}H, ^{13}C NMR, and Mass Spectra of New Compounds ………………………… S20

General Experimental

All reactions were carried out in oven-dried glassware (120 °C) under an atmosphere of nitrogen unless as indicated otherwise. Dichloromethane, ethyl acetate, and hexanes from Mallinckrodt Chemical Co. were dried and distilled from CaH_2. THF from Mallinckrodt Chemicals Co. was dried by distillation from sodium and benzophenone under an atmosphere of nitrogen. 2-Acetylpyridine, 4-methoxybenzophenone, methyl 2-chloroacetate, and phenacyl bromide were purchased from Fluka. Benzylacetone, 4-bromotoluene, 3,5-dimethoxy chlorobenzene, 4-heptanone, and α-tetralone were purchased from TCI. α-Bromo-γ-butyrolactone, cyclopentanone, cyclohexanone, cyclopropyl phenyl ketone, and 3,4-methylene dioxybenzaldehyde were purchased from Aldrich. Allyl bromide, bicyclo[3.2.0]hept-2-en-6-one, *n*-bromobutane, *tert*-butyl bromide, 2-fluoro benzaldehyde, 4-methoxybenzaldehyde, and 1-naphthaldehyde were purchased from Alfa. Sodium Silica gel Stage 1 (Na@SiO_2 ~ 35–40% Na) was purchased from Aldrich.

Analytical thin layer chromatography (TLC) was performed on precoated plates (silica gel 60 F-254), purchased from Merck Inc. Purification by gravity column chromatography was carried out by use of Silicycle ultra pure silica gel (particle size 40-63 μM, 230-400 mesh).

Infrared (IR) spectra were measured on a Perkin–Elmer Model Spectrum 100 spectrophotometer. Absorption intensities are recorded by the following abbreviations: s = strong; m = medium; and w = weak. Proton NMR spectra were obtained on a Varian Mercury-400 (400MHz) spectrometer by use of chloroform-*d* ($CDCl_3$) as the solvent. Proton NMR chemical shifts were referenced to residual protonated solvent (δ 7.24 for chloroform). Carbon-13 NMR spectra were obtained

on a Varian Mercury-400 (100MHz) spectrometer by use of chloroform-*d* ($CDCl_3$) as the solvent. Carbon-13 chemical shifts are referenced to the center of the $CDCl_3$ triplet (δ 77.0 ppm). Multiplicities are recorded by the following abbreviations: s, singlet; d, doublet; t, triplet; q, quartet; m, multiplet; *J*, coupling constant (hertz). High-resolution mass spectra were obtained by means of a JEOL JMS-700 mass spectrometer.

The Standard Procedure 1 for the Syntheses of Alcohols and Amines. A reaction flask equipped with a magnetic stirring bar, rubber stopper, and nitrogen balloon was charged with Na@SiO_2 (1.3–1.7 equiv). To this reaction mass was added THF (2.0–4.0 mL) via syringe at room temperature. Then an aldehyde, ketone, or imine (**1** or **8**, 1.0 equiv) was premixed with an organohalide (**2**, 1.2 equiv). The mixture was diluted with THF (0.50 mL) and the resultant solution was injected into the reaction mass via syringe. After the reaction mixture was stirred at 25 °C for 1.0–3.0 h, the inorganic residue was filtered. The filtrate was concentrated under reduced pressure and then purified by use of column chromatography packed with silica gel and eluented with a mixture of EtOAc and hexanes to give the desired alcohols **3** or amines **9**.

2-(3,5-Dimethoxyphenyl)pentan-2-ol (3a). The Standard Procedure **1** was followed by use of Na@SiO_2 (17.4 mg, 0.758 mmol, 1.3 equiv) in THF (2.0 mL), 2-pentanone (**1a**, 50.1 mg, 0.582 mmol, 1.0 equiv), and 1-chloro-3,5-dimethoxybenzene (**2a**, 0.121 g, 0.699 mmol, 1.2 equiv). After the reaction mixture was stirred at 25 °C for 1.0 h, the residue was purified by use of column chromatography (2.0% EtOAc in hexanes as the eluent) to give the desired alcohol **3a** (0.110 g, 0.489 mmol) in 84% yield as a colorless oil: ^1H NMR ($CDCl_3$, 400 MHz) δ 6.57–6.56 (m, 2 H, 2 × ArH), 6.32 (t, J = 2.0 Hz, 1 H, ArH), 3.78 (s, 6 H, 2 × OCH_3), 1.76–1.70 (m, 2 H, CH_2), 1.67 (s, 1 H,

OH), 1.50 (s, 3 H, CH_3), 1.31–1.23 (m, 2 H, CH_2), 0.84 (t, J = 7.2 Hz, 3 H, CH_3); ^{13}C NMR ($CDCl_3$, 100 MHz) δ 160.80, 150.89, 103.25, 97.98, 74.79, 55.43 (OCH_3), 46.33, 30.05, 17.20, 14.33; IR (neat) 3522 (s, OH), 2950 (s), 1596 (s), 1456 (s), 1421 (s), 1330 (m), 1201 (s), 1149 (s), 1053 (m), 831 (s) cm^{-1}; ESIMS calcd for ($C_{13}H_{20}O_3$): 224.1412; found 224.1411.

2-(3,5-Dimethoxyphenyl)-4-phenylbutan-2-ol (3b). The Standard Procedure **1** was followed by use of Na@SiO_2 (12.5 mg, 0.544 mmol, 1.6 equiv) in THF (4.0 mL), benzyl acetone (**1b**, 50.3 mg, 0.339 mmol, 1.0 equiv), and 1-chloro-3,5-dimethoxybenzene (**2a**, 70.3 mg, 0.407 mmol, 1.2 equiv). After the reaction mixture was stirred at 25 °C for 2.0 h, the residue was purified by use of column chromatography (4.0% EtOAc in hexanes as the eluent) to give the desired alcohol **3b** (87.5 mg, 0.305 mmol) in 90% yield as a colorless oil: ^1H NMR ($CDCl_3$, 400 MHz) δ 7.26–7.22 (m, 2 H, 2 × ArH), 7.17–7.11 (m, 3 H, 3 × ArH), 6.63 (s, 2 H, 2 × ArH), 6.36 (s, 1 H, ArH), 3.81 (s, 6 H, 2 × OCH_3), 2.66–2.59 (m, 1 H, CH), 2.50–2.42 (m, 1 H, CH), 2.16–2.03 (m, 2 H, CH_2), 1.82 (s, 1 H, OH), 1.58 (s, 3 H, CH_3); ^{13}C NMR ($CDCl_3$, 100 MHz) δ 160.75, 150.34, 142.23, 128.33, 128.29, 125.71, 103.28, 98.25, 74.80, 55.31, 45.74, 30.51, 30.40; IR (neat) 3478 (s, OH), 2945 (s), 2104 (w), 1607 (s), 1423 (s), 1341 (s), 1196 (m), 1152 (s), 1048 (m), 924 (m), 842 (s) cm^{-1}; ESIMS calcd for ($C_{18}H_{23}O_3$): 286.1570; found 286.1570.

(±)-(1*S*5*R*6*R*)- and (1*S*5*R*6*S*)-6-(3,5-Dimethoxyphenyl)bicyclo[3.2.0]hept-2-en-6-ol (3c). The Standard Procedure **1** was followed by use of Na@SiO_2 (17.1 mg, 0.744 mmol, 1.6 equiv) in THF (4.0 mL), (±)-*cis*-bicyclo[3.2.0]hept-2-en-6-one (**1c**, 50.2 mg, 0.464 mmol, 1.0 equiv), and 1-chloro-3,5-dimethoxybenzene (**2a**, 96.1 mg, 0.557 mmol, 1.2 equiv). After the reaction mixture was stirred at 25 °C for 1.0 h, the residue was purified by use of column chromatography (2.0% EtOAc in hexanes as

the eluent) to give the desired alcohol **3c** (0.103 g, 0.415 mmol) in 91% yield as a colorless oil: ^{1}H NMR ($CDCl_3$, 400 MHz) δ 6.62–6.61 (m, 2 H, 2 × ArH), 6.35–6.34 (m, 1 H, ArH), 5.93 (s, 2 H, CH), 3.79 (s, 6 H, 2 × OCH_3), 3.42–3.30 (m, 1 H, CH), 3.20–3.18 (m, 1 H, CH), 2.94–2.89 (m, 1 H, CH), 2.83–2.77 (m, 1 H, CH), 2.51–2.45 (m, 1 H, CH), 2.17 (s, 1 H, OH), 2.05–2.01 (m, 1 H, CH); ^{13}C NMR ($CDCl_3$, 100 MHz) δ 160.76, 150.00, 135.41, 132.83, 103.03, 98.56, 77.00, 55.34, 47.99, 44.47, 39.48, 32.89; IR (neat) 3439 (s, OH), 2071 (w), 1509 s (m), 1363 (m), 1248 (m), 1032 (m), 963 (m), 820 (m) cm^{-1}; ESIMS calcd for ($C_{15}H_{18}O_3$): 246.1256; found 246.1256.

1-(3,5-Dimethoxyphenyl)cyclopentanol (3d). The Standard Procedure **1** was followed by use of Na@SiO_2 (17.8 mg, 0.776 mmol, 1.3 equiv) in THF (3.0 mL), cyclopentanone (**1d**, 50.1 mg, 0.595 mmol, 1.0 equiv), and 1-chloro-3,5-dimethoxybenzene (**2a**, 0.123 g, 0.715 mmol, 1.2 equiv). After the reaction mixture was stirred at 25 °C for 2.0 h, the residue was purified by use of column chromatography (1.0% EtOAc in hexanes as the eluent) to give the desired alcohol **3d** (0.114 g, 0.513 mmol) in 86% yield as a light yellow oil: ^{1}H NMR ($CDCl_3$, 400 MHz) δ 6.63 (s, 2 H, 2 × ArH), 6.33 (t, J = 2.2 Hz, 1 H, ArH), 3.76 (s, 6 H, 2 × OCH_3), 2.00–1.91 (m, 6 H, 3 × CH_2), 1.82–1.79 (m, 2 H, CH_2); ^{13}C NMR ($CDCl_3$, 100 MHz) δ 160.43, 149.78, 103.44, 98.41, 83.53, 55.30, 41.86, 23.83; IR (neat) 3522 (s, OH), 2945 (s), 1593 (s), 1454 (s), 1418 (m), 1336 (s), 1199 (m), 1146 (s), 1053 (s), 971 (w), 836 (s) cm^{-1}; ESIMS calcd for ($C_{13}H_{18}O_3$): 222.1256; found 222.1257.

1-(3,5-Dimethoxyphenyl)cyclohexanol (3e). The Standard Procedure **1** was followed by use of Na@SiO_2 (17.6 mg, 0.765 mmol, 1.5 equiv) in THF (3.0 mL), cyclohexanone (**1e**, 50.1 mg, 0.510 mmol, 1.0 equiv), and 1-chloro-3,5-dimethoxybenzene (**2a**, 0.106 g, 0.612 mmol, 1.2 equiv). After the reaction mixture was stirred at 25 °C for 2.0 h, the residue was purified by use of column

chromatography (1.0% EtOAc in hexanes as the eluent) to give the desired alcohol **3e** (0.107 g, 0.454 mmol) in 88% yield as a light yellow oil: ^{1}H NMR ($CDCl_3$, 400 MHz) δ 6.66–6.65 (m, 2 H, 2 × ArH), 6.33 (t, J = 2.1 Hz, 1 H, ArH), 3.76 (s, 6 H, 2 × OCH_3), 1.83–1.67 (m, 6 H, 3 × CH_2), 1.63–1.60 (m, 2 H, CH_2), 1.58–1.56 (m 2 H, CH_2); ^{13}C NMR ($CDCl_3$, 100 MHz) δ 160.65, 152.28, 103.00, 98.40, 73.31, 55.31, 38.72, 25.49, 22.13; IR (neat) 3516 (s, OH), 2873 (s), 1596 (s), 1421 (s), 1338 (s), 1201 (s), 1149 (s), 1050 (s), 927 (w), 828 (s) cm^{-1}; ESIMS calcd for ($C_{14}H_{20}O_3$): 236.1412; found 236.1417.

Cyclopropyl(phenyl)(*p*-tolyl)methanol (3f). The Standard Procedure **1** was followed by use of Na@SiO_2 (11.1 mg, 0.483 mmol, 1.4 equiv) in THF (4.0 mL), cyclopropyl phenyl ketone (**1f**, 50.3 mg, 0.344 mmol, 1.0 equiv), and 4-bromotoluene (**2b**, 70.6 mg, 0.413 mmol, 1.2 equiv). After the reaction mixture was stirred at 25 °C for 1.5 h, the residue was purified by use of column chromatography (4.0% EtOAc in hexanes as the eluent) to give the desired alcohol **3f** (72.9 mg, 0.306 mmol) in 89% yield as a colorless oil: ^{1}H NMR ($CDCl_3$, 400 MHz) δ 7.45–7.43 (m, 2 H, 2 × ArH), 7.34–7.29 (m, 4 H, 4 × ArH), 7.25–7.24 (m, 1 H, ArH), 7.13–7.11 (m, 2 H, 2 × ArH), 2.33 (s, 3 H, CH_3), 1.63–1.59 (m, 1 H, **H**C(CH_2)$_2$, 0.60–0.47 (m, 4 H, 2 × CH_2). The spectroscopic data are in accordance with literature data[1].

1-(*p*-Tolyl)-1,2,3,4-tetrahydronaphthalen-1-ol (3g). The Standard Procedure **1** was followed by use of Na@SiO_2 (23.8 mg, 1.03 mmol, 1.5 equiv) in THF (4.0 mL), α–tetralone (**1g**, 0.101 g, 0.691 mmol, 1.0 equiv), and 4-bromotoluene (**2b**, 0.142 g, 0.829 mmol, 1.2 equiv). After the reaction mixture was stirred at 25 °C for 2.0 h, the residue was purified by use of column chromatography (5.0% EtOAc in hexanes as the eluent) to give the desired alcohol **3g** (0.139 g, 0.581 mmol) in 85% yield as a colorless oil: ^{1}H NMR ($CDCl_3$, 400 MHz) δ 7.20–7.18 (m, 2 H, 2 × ArH), 7.16–7.12

(m, 3 H, 3 × ArH), 7.10–7.08 (m, 2 H, 2 × ArH), 7.05–7.03 (m, 1 H, ArH), 2.89–2.85 (m, 2 H, CH_2), 2.32 (s, 3 H, CH_3), 2.16 (s, 1 H, OH), 2.14–2.09 (m, 2 H, CH_2), 2.06–1.92 (m, 1 H, CH), 1.80–1.72 (m, 1 H, CH). The spectroscopic data are in accordance with literature data[2].

(4-Methoxyphenyl)(phenyl)(*p*-tolyl)methanol (3h). The Standard procedure **1** was followed by use of Na@SiO_2 (15.7 mg, 0.680 mmol, 4.0 equiv) in THF (4.0 mL) was added 4-methoxybenzophenone (**1h**, 0.102 g, 0.480 mmol, 1.0 equiv), and 4-bromotoluene (**2b**, 98.6 mg, 0.577 mmol, 1.2 equiv). After the reaction mixture was stirred at 25 °C for 2.0 h, the residue was purified by use of column chromatography (5.0% EtOAc in hexanes as the eluent) to give the desired alcohol **3h** (0.129 g, 0.423 mmol) in 88% yield as a light yellow oil: ^{1}H NMR ($CDCl_3$, 400 MHz) δ 7.28–7.24 (m, 6 H, 6 × ArH), 7.16–7.08 (m, 5 H, 5 × ArH), 6.82–6.80 (m, 2 H, 2 × ArH), 3.78 (s, 3 H, OCH_3), 2.32 (s, 3 H, CH_3). The spectroscopic data are in accordance with literature data[2].

2,2-Dimethyl-1-(*p*-tolyl)propan-1-ol (3i). The Standard procedure **1** was followed by use of Na@SiGel (15.4 mg, 0.669 mmol, 1.6 equiv) in THF (4.0 mL) was added 4-tolaldehyde (**1i**, 50.2 mg, 0.418 mmol, 1.0 equiv), and 2-chloro-2-methylpropane (**2c**, 46.4 mg, 0.501 mmol, 1.2 equiv). After the reaction mixture was stirred at room temperature for 1.5 h and then worked up, the residue was purified by use of column chromatography (2.0% EtOAc in hexanes as the eluent) to give the desired alcohol **3i** (59.6 mg, 0.334 mmol) in 80% yield as a yellow oil: ^{1}H NMR ($CDCl_3$, 400 MHz) δ 7.18–7.16 (m, 2 H, 2 × ArH), 7.11–7.09 (m, 2 H, 2 × ArH), 4.35 (s, 1 H, **H**C(OH)C$(CH_3)_3$ 2.32 (s, 3 H, CH_3), 1.77 (s, 1 H OH), 0.94 (s, 9 H, 3 × CH_3). The spectroscopic data were found to be in accordance with literature data[3].

1-(2,3-Dimethoxyphenyl)pentan-1-ol (3j). The Standard procedure **1** was followed by use of Na@SiGel (11.1 mg, 0.483 mmol, 1.6 equiv) in THF (4.0 mL) was added 2,3-dimethoxy benzaldehyde (**1j**, 50.3 mg, 0.303 mmol, 1.0 equiv), and *n*-butylbromide (**2d**, 49.8 mg, 0.363 mmol, 1.2 equiv). After the reaction mixture was stirred at room temperature for 1.5 h and then worked up, the residue was purified by use of column chromatography (5.0% EtOAc in hexanes as the eluent) to give the desired alcohol **3j** (52.2 mg, 0.233 mmol) in 77% yield as a brown oil: ^{1}H NMR ($CDCl_3$, 400 MHz) δ 7.03 (t, J = 8.0 Hz, 1 H, ArH), 6.92 (d, J = 8.0 Hz, 1 H, ArH), 6.82 (d, J = 8.0 Hz, 1 H, ArH), 4.89 (br, 1 H, **H**C(OH)CH_2), 3.86 (s, 3 H, OCH_3), 3.85 (s, 3 H, OCH_3), 1.89–1.71 (m, 2 H, $\mathbf{H_2}$C(CH)OH), 1.47–1.26 (m, 4 H, 2 × CH_2), 0.86 (t, J = 6.8 Hz, CH_3). The spectroscopic data were found to be in accordance with literature data[4].

(20-Butyl)pregna-5-en-3β,20-diol (3k). The Standard procedure 1 was followed by use of Na@SiGel (6.07 mg, 0.264 mmol, 1.6 equiv) in THF (4.0 mL) was added pregnenolone (**1k**, 49.1 mg, 0.155 mmol, 1.0 equiv), and *n*-butylbromide (**2d**, 49.8 mg, 0.186 mmol, 1.2 equiv). After the reaction mixture was stirred at room temperature for 3.0 h and then worked up, the residue was purified by use of column chromatography (7.0% EtOAc in hexanes as the eluent) to give the desired alcohol **3k** (45.3 mg, 0.121 mmol) in 78% yield as white solids: mp (recrystallized from EtOAc/hexanes) 96.8–97.5 °C; ^{1}H NMR ($CDCl_3$, 400 MHz) δ 5.32–5.31 (m, 1 H, HC=C), 3.52–3.45 (m, 1 H, CHO), 2.29–2.17 (m, 2 H, CH_2CO), 2.07–2.04 (m, 1 H), 1.96–1.93 (m, 1 H), 1.81–1.74 (m, 2 H), 1.73–1.71 (m, 1 H), 1.70–1.40 (m, 11 H), 1.33–1.04 (m, 11 H), 0.98 (s, 3 H, CH_3), 0.92–0.83 (m, 3 H, CH_3), 0.83 (s, 3 H, CH_3). The spectroscopic data were found to be in accordance with literature data[5].

***tert*-Butyl 3-(1-hydroxybut-3-en-1-yl)-indole-1-carboxylate (3l).** The Standard procedure 1 was followed by use of Na@SiGel (7.68 mg, 0.334 mmol, 1.6 equiv) in THF (4.0 mL) was added *N*-protected indole[6] (**1l**, 51.2 mg, 0.209 mmol, 1.0 equiv), and allyl bromide (**2e**, 30.3 mg, 0.250 mmol, 1.2 equiv). After the reaction mixture was stirred at room temperature for 2.5 h and then worked up, the residue was purified by use of column chromatography (5.0% EtOAc in hexanes as the eluent) to give the desired alcohol **3l** (48.1 mg, 0.167 mmol) in 80% yield as a yellow oil: ^{1}H NMR ($CDCl_3$, 400 MHz) δ 8.12 (br, 1 H, ArH), 7.65 (d, J = 7.8 Hz, 1 H, ArH), 7.54 (s, 1 H, NCH=C), 7.31 (t, J = 7.8 Hz, 1 H, ArH), 7.23–7.21 (m, 1 H, ArH), 5.88–5.84 (m, 1 H, C**H**=CH_2), 5.22–5.15 (m, 2 H, =CH_2), 5.02–4.99 (m, 1 H, CHO), 2.75–2.64 (m, 2 H, CH_2CO), 1.65 (s, 9 H, 3 × CH_3); ^{13}C NMR (100 MHz, $CDCl_3$) δ 149.70 (C=O), 136.02, 134.33, 128.51, 124.51, 123.30, 122.54, 122.45, 119.71, 118.55, 115.36, 83.71, 67.08, 41.85, 28.18 (CH_3); IR (neat) 3423 (m, OH), 2978 (m), 1725 (s, C=O), 1451 (s), 1374 (s), 1254 (s), 1158 (s), 1092 (m) cm^{-1}; ESIMS calcd for ($C_{17}H_{21}NO_3$ + Na): 310.1420; found 310.1423.

***N*-(2,2-Dimethyl-1-phenylpropyl)-2,3-dimethylaniline (9a).** The Standard procedure **1** was followed by use of Na@SiO_2 (9.35 mg, 0.407 mmol, 1.7 equiv) in THF (4.0 mL) was added *N*-benzylidene-2,3-dimethylaniline (**8a**, 50.1 mg, 0.239 mmol, 1.0 equiv), and 2-chloro-2-methylpropane (**2c**, 26.6 mg, 0.287 mmol, 1.2 equiv). After the reaction mixture was stirred at 25 °C for 1.5 h, the residue was purified by use of column chromatography (10% EtOAc in hexanes as the eluent) to give the desired amine **9a** (57.5 mg, 0.215 mmol) in 90% yield as yellow solids: mp (recrystallized from EtOAc/hexanes) 79.4–80.3 °C; ^{1}H NMR ($CDCl_3$, 400 MHz) δ 7.29–7.17 (m, 5 H, 5 × ArH), 6.76 (t, J = 8.0 Hz, 1 H, ArH), 6.44 (d, J = 7.6 Hz, 1 H, ArH), 6.15 (d, J = 8.4 Hz, 1 H, ArH), 4.05 (s, 1 H, CH), 2.24 (s, 3 H, CH_3), 2.15 (s, 3

H, CH_3), 1.00 (s, 9 H, 3 × CH_3); ^{13}C NMR ($CDCl_3$, 100MHz) δ 145.30, 141.21, 136.12, 128.39, 127.64, 126.69, 125.97, 119.70, 118.69, 108.79, 67.04, 30.01, 27.19, 20.70, 12.50; IR (neat) 3390 (m, NH), 2897 (s), 1596 (s), 1464 (s), 1421 (w), 1302 (w), 1097 (m), 804 (m) cm^{-1}; ESIMS calcd for ($C_{19}H_{25}N$): 267.1987; found 267.1989.

***N*-[2,2-Dimethyl-1-(pyridin-2-yl)propyl]-2,3-dimethylaniline (9b)**. The Standard procedure **1** was followed by use of Na@SiO_2 (7.48 mg, 0.325 mmol, 1.7 equiv) in THF (4.0 mL) was added 2,3-dimethyl-*N*-(pyridin-2-ylmethylene)aniline (**8b**, 40.2 mg, 0.191 mmol, 1.0 equiv), and 2-chloro-2-methylpropane (**2c**, 21.2 mg, 0.229 mmol, 1.2 equiv). After the reaction mixture was stirred at 25 °C for 3.0 h, the residue was purified by use of column chromatography (12% EtOAc in hexanes as the eluent) to give the desired amine **9b** (45.1 mg, 0.167 mmol) in 87% yield as brown sticky solids: mp (recrystallized from EtOAc/hexanes) 81.1−82.2 °C; ^{1}H NMR ($CDCl_3$, 400 MHz) δ 8.55–8.54 (m, 1 H, H-6), 7.53–7.50 (m, 1 H, H-4), 7.21–7.07 (m, 2 H, H-3 + H-5), 6.82 (t, J = 7.6 Hz, 1 H, ArH), 6.46 (d, J = 7.6 Hz, 1 H, ArH), 6.32 (d, J = 8.0 Hz, 1 H, ArH), 4.64 (br, 1 H, NH), 4.25–4.24 (m, 1 H, CH), 2.19 (s, 3 H, CH_3), 2.15 (s, 3 H, CH_3), 1.08 (s, 9 H, 3 × CH_3); ^{13}C NMR ($CDCl_3$, 100 MHz) δ 161.40, 148.59, 145.96, 136.37, 135.58, 125.98, 122.96, 121.76, 120.48, 118.99, 109.05, 67.88, 35.62, 27.17, 20.74, 12.64; IR (neat) 3394 (w, NH), 2957 (s), 1887 (w), 1587 (s), 1476 (s), 1434 (w), 1316 (w), 1097 (m), 804 (m) cm^{-1}; ESIMS calcd for ($C_{18}H_{24}N_2$): 268.1939; found 268.1938.

2,3-Dimethyl-*N*-(1-phenylbut-3-en-1-yl)aniline (9c). The Standard Procedure **1** was followed by use of Na@SiO_2 (9.39 mg, 0.407 mmol, 1.7 equiv) in THF (4.0 mL), *N*-benzylidene-2,3-dimethylaniline (**8a**, 50.2 mg, 0.240 mmol, 1.0 equiv), and allyl bromide (**2e**, 34.8 mg, 0.288 mmol, 1.2 equiv). After the reaction mixture was stirred

at 25 °C for 1.5 h, the residue was purified by use of column chromatography (10% EtOAc in hexanes as the eluent) to give the desired amine **9c** (57.6 mg, 0.229 mmol) in 88% yield as a colorless oil: ^{1}H NMR ($CDCl_3$, 400 MHz) δ 7.36–7.27 (m, 3 H, 3 × ArH), 7.23–7.19 (m, 2 H, 2 × ArH), 6.80 (t, J = 7.6 Hz, 1 H, ArH), 6.50 (d, J = 7.6 Hz, 1 H, ArH), 6.16 (d, J = 7.6 Hz, 1 H, ArH), 5.83–5.73 (m, 1 H, HC=), 5.24–5.19 (m, 2 H, =CHH), 4.40–4.36 (m, 1 H, **H**C(NH)CH_2), 4.09 (s, 1 H, NH), 2.67–2.46 (m, 2 H, **H**$_2$C(HC=)), 2.26 (s, 3 H, CH_3), 2.16 (s, 3H, CH_3); ^{13}C NMR ($CDCl_3$, 100 MHz) δ 145.12, 143.86, 136.13, 135.04, 128.55, 126.87, 126.19, 125.95, 120.23, 119.23, 118.27, 109.42, 57.12, 43.72, 20.69, 12.48; IR (neat) 3284 (br, NH), 2885 (s), 1884 (w), 1591 (s), 1483 (s), 1421 (w), 1321 (w), 1041 (m), 804 (m) cm^{-1}; ESIMS calcd for ($C_{18}H_{21}N$): 251.1674; found 251.1675.

The Standard Procedure 2 for the Synthesis of Epoxides. A reaction flask equipped with a magnetic stirring bar, rubber stopper, and nitrogen balloon was charged with Na@SiO_2 (1.8–2.0 equiv). To this reaction mass was added THF (1.0–2.0 mL) via syringe at room temperature. Then an aldehyde **1** was premixed with an organohalide **4** or **10** (1.2 equiv). The mixture was diluted with THF (0.50 mL) and the resultant solution was injected into the reaction mass via syringe. After the reaction mixture was stirred at 25 °C for 0.50–2.0 h, the inorganic residue was filtered. The filtrate was concentrated under reduced pressure and then purified by use of column chromatography packed with silica gel and eluented with a mixture of EtOAc and hexanes to give the desired *trans*-epoxides **5** or **11**.

***trans*-Methyl 3-(*p*-Tolyl)glycidate (5i)**. The Standard Procedure **2** was followed by use of Na@SiO_2 (11.1 mg, 0.482 mmol, 1.8 equiv) in THF (1.0 mL), 4-toluenebenzaldehyde (**1i**, 40.3 mg, 0.335 mmol, 1.0 equiv), and methyl chloroacetate **4a** (43.7 mg, 0.403 mmol, 1.2 equiv). After the reaction mixture was stirred at 25 °C

for 30 min, the residue was purified by use of column chromatography (6.0% EtOAc in hexanes as the eluent) to give the *trans*-epoxide **5i** (52.9 mg, 0.275 mmol) exclusively in 82% yield as a colorless oil: ^{1}H NMR ($CDCl_3$, 400 MHz) δ 7.15 (s, 4 H, 4 × ArH), 4.04 (d, J = 1.8 Hz, 1 H, OCHPh), 3.80 (s, 3 H, $COOCH_3$), 3.49 (d, J = 1.8 Hz, 1 H, OCHC=O), 2.33 (s, 3 H, CH_3). The spectroscopic data are in accordance with literature data[7].

***trans*-Methyl 3-(2,3-Dimethoxyphenyl)glycidate (5j)**. The Standard Procedure **2** was followed by use of Na@SiO_2 (13.2 mg, 0.574 mmol, 1.9 equiv) in THF (1.0 mL), 2,3-dimethoxybenzaldehyde (**1j**, 50.2 mg, 0.302 mmol, 1.0 equiv), and methyl chloroacetate (**4a**, 39.3 mg, 0.363 mmol, 1.2 equiv). After the reaction mixture was stirred at 25 °C for 1.0 h, the residue was purified by use of column chromatography (8.0% EtOAc in hexanes as the eluent) to give the *trans*-epoxide **5j** (60.5 mg, 0.254 mmol) exclusively in 84% yield as a colorless oil: ^{1}H NMR ($CDCl_3$, 400 MHz) δ 7.02 (t, J = 8.0 Hz, 1 H, ArH), 6.87 (dd, J = 8.0, 1.6 Hz, 1 H, ArH), 6.69 (dd, J = 8.0, 1.6 Hz, 1 H, ArH), 4.38 (d, J = 1.8 Hz, 1 H, OCHPh), 3.82 (s, 6 H, 2 × OCH_3), 3.79 (s, 3 H, $COOCH_3$), 3.46 (d, J = 1.8 Hz, 1 H, OCHC=O). The spectroscopic data are in accordance with literature data[8].

***trans*-Methyl 3-(2-Fluorophenyl)glycidate (5k)**. The Standard Procedure **2** was followed by use of Na@SiO_2 (16.7 mg, 0.727 mmol, 1.8 equiv) in THF (1.0 mL), 2-fluorobenzaldehyde (**1m**, 52.2 mg, 0.404 mmol, 1.0 equiv), and methyl chloroacetate in (**4a**, 52.7 mg, 0.485 mmol, 1.2 equiv). After the reaction mixture was stirred at 25 °C for 30 min, the residue was purified by use of column chromatography (5.0% EtOAc in hexanes as the eluent) to give the *trans*-epoxide **5k** (68.3 mg, 0.348 mmol) exclusively in 86% yield as a yellow oil: ^{1}H NMR ($CDCl_3$, 400 MHz) δ 7.33–7.28 (m, 1 H, ArH), 7.19–7.11 (m, 2 H, 2 × ArH), 7.08–7.03 (m, 1 H, ArH), 4.35 (d, J =

2.0 Hz, 1 H, OCHPh), 3.82 (s, 3 H, $COOCH_3$), 3.50 (d, J = 2.0 Hz, 1 H, OCHC=O). The spectroscopic data are in accordance with literature data[9].

***trans*-Methyl 3-(Naphthalen-1-yl)glycidate (5l).** The Standard Procedure **2** was followed by use of Na@SiO_2 (13.3 mg, 0.576 mmol, 1.8 equiv) in THF (1.0 mL), 1-napthaldehyde (**1n**, 50.1 mg, 0.321 mmol, 1.0 equiv), and methyl chloroacetate (**4a**, 41.7 mg, 0.385 mmol, 1.2 equiv). After the reaction mixture was stirred at 25 °C for 30 min, the residue was purified by use of column chromatography (6.0% EtOAc in hexanes as the eluent) to give the *trans*-epoxide **5l** (63.7 mg, 0.279 mmol) exclusively in 87% yield as a colorless oil: ^{1}H NMR ($CDCl_3$, 400 MHz) δ 8.03 (d, J = 8.0 Hz, 1 H, ArH), 7.89−7.87 (m, 1 H, ArH), 7.83 (d, J = 8.0 Hz, 1 H, ArH), 7.58−7.51 (m, 2 H, 2 × ArH), 7.48−7.43 (m, 2 H, 2 × ArH), 4.72 (d, J = 1.0 Hz, 1 H, OCHPh), 3.88 (s, 3 H, $COOCH_3$), 3.53 (d, J = 1.0 Hz, 1 H, OCHC=O). The spectroscopic data are in accordance with literature data[10].

***trans*-Methyl 3-(Benzo[*d*][1,3]dioxol-5-yl)glycidate (5m).** The Standard Procedure **2** was followed by use of Na@SiO_2 (13.8 mg, 0.601 mmol, 1.8 equiv) in dry THF (1.0 mL), 3,4-dioxymethylene benzaldehyde (**1o**, 50.2 mg, 0.334 mmol, 1.0 equiv), and methyl chloroacetate in (**4a**, 43.5 mg, 0.401 mmol, 1.2 equiv). After the reaction mixture was stirred at 25 °C for 30 min, the residue was purified by use of column chromatography (8.0% EtOAc in hexanes as the eluent) to give the *trans*-epoxide **5m** (59.4 mg, 0.267 mmol) exclusively in 80% yield as a yellow oil: ^{1}H NMR ($CDCl_3$, 400 MHz) δ 6.79−6.76 (m, 2 H, 2 ArH), 6.68 (s, 1 H, ArH), 5.95 (s, 2 H, OCH_2O), 4.00 (d, J = 2.0 Hz, 1 H, OCHPh), 3.80 (s, 3 H, $COOCH_3$), 3.44 (d, J = 2.0 Hz, 1 H, OCHC=O). The spectroscopic data are in accordance with literature data[11].

2-(2,3-Dimethoxyphenyl)-1,5-dioxaspiro[2.4]heptan-4-one (5n). The Standard Procedure **2** was followed by use of Na@SiO_2 (13.2 mg, 0.574 mmol, 1.8 equiv) in

dry THF (4.0 mL), 2,3-dimethoxybenzaldehyde (**1j**, 50.2 mg, 0.302 mmol, 1.0 equiv), and α-bromobutyrolactone (**4b**, 59.8 mg, 0.363 mmol, 1.2 equiv). After the reaction mixture was stirred at 25 °C for 2.0 h, the residue was purified by use of column chromatography (10% EtOAc in hexanes as the eluent) to give the epoxide **5n** (65.4 mg, 0.261 mmol) in 87% yield as white solids: mp (recrystallized from EtOAc/hexanes) 92.1−92.2 °C; ^{1}H NMR ($CDCl_3$, 400 MHz) δ 7.05 (t, J = 7.9 Hz, 1 H, ArH), 6.91 (dd, J = 7.9, 1.2 Hz, 1 H, ArH), 6.73 (dd, J = 7.9, 1.2 Hz, 1 H, ArH), 4.54 (s, 1 H, OCHPh), 4.52–4.46 (m, 1 H, CH), 4.33–4.26 (m, 1 H, CH), 3.88 (s, 3 H, OCH_3), 3.86 (s, 3 H, OCH_3), 2.43–2.35 (m, 1 H, CH), 1.99–1.92 (m, 1 H, CH); ^{13}C NMR ($CDCl_3$, 100 MHz) δ 173.79 (C=O), 152.39, 147.79, 127.26, 124.21, 118.03, 112.86, 64.80, 61.10, 60.84, 59.18 (OCH_3), 55.78 (OCH_3), 23.09; IR (neat) 2791 (br, C=CH), 1755 (s, C=O), 1515 (m, Ar), 1364 (m, Ar), 1205 (s, C–O), 1024 (m), 963 (w) cm^{-1}; ESIMS calcd for ($C_{13}H_{14}O_5$): 250.0841; found 250.0843.

***trans*-Epoxy-3-(2,3-dimethoxyphenyl)-1-phenyl propan-1-one (5o).** The Standard Procedure **2** was followed by use of Na@SiO_2 (12.5 mg, 0.544 mmol, 1.8 equiv) in dry THF (1.0 mL), 2,3-dimethoxybenzaldehyde (**1j,** 50.1 mg, 0.301 mmol, 1.0 equiv), and phenacyl bromide (**4c**, 72.1 mg, 0.361 mmol, 1.2 equiv). After the reaction mixture was stirred at 25 °C for 30 min, the residue was purified by use of column chromatography (8.0% EtOAc in hexanes as the eluent) to give the *trans*-epoxide **5o** (77.2 mg, 0.271 mmol) exclusively in 90% yield as white solids: mp (recrystallized from EtOAc/hexanes) 110.1−111.2 °C; ^{1}H NMR ($CDCl_3$, 400 MHz) δ 8.03–8.01 (m, 2 H, 2 × ArH), 7.61–7.57 (m, 1 H, ArH), 7.49–7.45 (m, 1 H, ArH), 7.08 (t, J = 7.9 Hz, 1 H, ArH), 6.91 (dd, J = 7.9, 1.4 Hz, 1 H, ArH), 6.87 (dd, J = 7.9, 1.4 Hz, 1 H, ArH), 4.35 (d, J = 2.0 Hz, 1 H, OCHPh), 4.24 (d, J = 2.0 Hz, 1 H, OCHC=O), 3.87 (s, 3 H, OCH_3), 3.81 (s, 3 H, OCH_3); ^{13}C NMR ($CDCl_3$, 100 MHz) δ 193.38 (C=O), 152.48,

148.09, 135.46, 133.88, 128.84, 128.80, 128.31, 124.50, 117.02, 112.70, 61.10, 60.28, 55.81, 55.75; IR (neat) 3071 (m, CH stretch), 1690 (s, C=O), 1591 (w, Ar), 1492 (s, Ar), 1451 (s, Ar), 1416 (s, Ar), 1229 (s, C–O), 889 (m), 757 (s) cm^{-1}; ESIMS calcd for ($C_{17}H_{16}O_4$): 284.1049; found 284.1047.

***trans*-Epoxy-3-(2-fluorophenyl)-1-phenyl propan-1-one (5p).** The Standard Procedure **2** was followed by use of Na@SiO_2 (16.8 mg, 0.727 mmol, 1.8 equiv) in dry THF (1.0 mL) was added 2-fluorobenzaldehyde (**1m**, 50.3 mg, 0.405 mmol, 1.0 equiv), and phenacylbromide (**4c**, 96.7 mg, 0.486 mmol, 1.2 equiv). After the reaction mixture was stirred at 25 °C for 30 min, the residue was purified by use of column chromatography (6.0% EtOAc in hexanes as the eluent) to give the *trans*-epoxide **5p** (83.6 mg, 0.344 mmol) exclusively in 85% yield as a light yellow oil: ^1H NMR ($CDCl_3$, 400 MHz) δ 8.03–8.00 (m, 2 H, 2 ArH), 7.61 (t, J = 7.6 Hz, 1 H, ArH), 7.51–7.47 (m, 2 H, 2 ArH), 7.34–7.31 (m, 2 H, 2 ArH), 7.18 (t, J = 8.0 Hz, 1 H, ArH), 7.11–7.06 (m, 1 H, ArH), 4.33 (d, J = 1.8 Hz, 1 H, OCHPh), 4.28 (d, J = 1.8 Hz, 1 H, OCHC=O). The spectroscopic data are in accordance with literature data[12].

***trans*-Epoxy-3-naphthyl-1-phenyl propan-1-one (5q).** The Standard Procedure **2** was followed by use of Na@SiO_2 (13.3 mg, 0.576 mmol, 1.8 equiv) in dry THF (1.0 mL), 1-napthaldehyde (**1n**, 50.1 mg, 0.321 mmol, 1.0 equiv), and phenacylbromide (**4c**, 76.7 mg, 0.385 mmol, 1.2 equiv). After the reaction mixture was stirred at 25 °C for 30 min, the residue was purified by use of column chromatography (8.0% EtOAc in hexanes as the eluent) to give the *trans*-epoxide **5q** (75.7 mg, 0.276 mmol) exclusively in 86% yield as a colorless oil: ^1H NMR ($CDCl_3$, 400 MHz) δ 8.07–8.05 (m, 2 H, 2 ArH), 7.99–7.97 (m, 1 H, ArH), 7.91 (d, J = 8.0 Hz, 1 H, ArH), 7.86 (d, J = 8.0 Hz, 1 H, ArH), 7.64–7.57 (m, 3 H, 3 ArH), 7.54-7.45 (m, 4 H, 4 ArH), 4.72 (d, J =

1.8 Hz, 1 H, OCHPh), 4.30 (d, J = 1.8 Hz, 1 H, OCHC=O). The spectroscopic data are in accordance with literature data[13].

***trans*-6-(*tert*-Butyl)-4-[3-(2,3-dimethoxyphenyl)oxiran-2-yl]-2*H*-chromen-2-one (11a).** The Standard Procedure **2** was followed by use of Na@SiO_2 (13.9 mg, 0.603 mmol, 2.0 equiv) in dry THF (2.0 mL), 2,3-dimethoxybenzaldehyde (**1j**, 50.3 mg, 0.303 mmol, 1.0 equiv), and 6-(*tert*-butyl)-4-(chloromethyl)-2*H*-chromen-2-one (**10a**, 91.3 mg, 0.363 mmol, 1.2 equiv). After the reaction mixture was stirred at 25 °C for 2.0 h, the residue was purified by use of column chromatography (9.0% EtOAc in hexanes as the eluent) to give the *trans*-epoxide **11a** (94.4 mg, 0.248 mmol) exclusively in 82% yield as pale yellow solids: mp (recrystallized from methanol/CH_2Cl_2) 133.4–134.2 °C; ^1H NMR ($CDCl_3$, 400 MHz) δ 7.60 (d, J = 2.0 Hz, 1 H, H-5'), 7.58–7.56 (m, 1 H, H-7'), 7.31–7.29 (m, 1 H, H-8'), 7.14–7.09 (m, 1 H, H-5), 6.96–6.90 (m, 2 H, H-4 + H-6), 6.51 (s, 1 H, H-3'), 4.16 (d, J = 1.8 Hz, 1 H, OCHPh), 4.10 (d, J = 1.8 Hz, 1 H, OCHC=C), 3.88 (s, 3 H, OCH_3), 3.80 (s, 3 H, OCH_3), 1.26 (s, 9 H, $C(CH_3)_3$); ^{13}C NMR ($CDCl_3$, 100 MHz) δ 161.06 (C=O), 152.52, 151.64, 150.98, 148.18, 147.55, 129.77, 129.36, 124.48, 119.82, 117.24, 117.02, 116.87, 113.02, 111.22, 61.11, 58.02 (OCH_3), 57.46 (OCH_3), 55.90, 34.61, 31.26; IR (neat) 2791 (br, C=CH), 1756 (s, C=O), 1514 (m, Ar), 1363 (m, Ar), 1204 (s, C–O), 1023 (m), 963 (w) cm^{-1}; ESIMS calcd for ($C_{23}H_{24}O_5$): 380.1624; found 380.1627.

***trans*-4-[3-(2,3-Dimethoxyphenyl)oxiran-2-yl]-7-methoxy-2*H*-chromen-2-one (11b).** The Standard Procedure **2** was followed by use of Na@SiO_2 (11.2 mg, 0.483 mmol, 2.0 equiv) in dry THF (2.0 mL), 2,3-dimethoxybenzaldehyde (**1j**, 40.2 mg, 0.242 mmol, 1.0 equiv), and 4-(chloromethyl)-7-methoxy-2*H*-chromen-2-one (**10b**, 65.2 mg, 0.290 mmol, 1.2 equiv). After the reaction mixture was stirred at 25 °C for 2.0 h, the residue was purified by use of column chromatography (11% EtOAc in

hexanes as the eluent) to give the *trans*-epoxide **11b** (74.6 mg, 0.211 mmol) exclusively in 87% yield as white solids: mp (recrystallized from methanol/CH_2Cl_2) 137.6–138.3 °C; ^{1}H NMR ($CDCl_3$, 400 MHz) δ 7.52 (d, J = 8.8 Hz, 1 H, H-5'), 7.10 (t, J = 8.2 Hz, 1 H, ArH), 6.92 (d, J = 8.2 Hz, 1 H, ArH), 6.88 (d, J = 8.2 Hz 1 H, ArH), 6.83 (s, 1 H, H-8'), 6.79 (dd, J = 8.8, 2.4 Hz, 1 H, H-6'), 6.35 (s, 1 H, H-3'), 4.13 (d, J = 1.8 Hz, 1 H, OCHPh), 4.03 (d, J = 1.8 Hz, 1 H, OCHC=C), 3.88 (s, 3 H, OCH_3), 3.85 (s, 3 H, OCH_3), 3.79 (s, 3 H, OCH_3); ^{13}C NMR ($CDCl_3$, 100 MHz) δ 162.86 (C=O), 161.23, 155.50, 152.45, 150.85, 147.90, 124.77, 124.55, 124.15, 116.83, 112.61, 112.58, 112.11, 107.73, 101.03, 61.13, 60.82, 57.80 (OCH_3), 57.64 (OCH_3), 55.77; IR (neat) 2945 (m, C=CH), 1723 (s, C=O), 1610 (s, Ar), 1479 (s, Ar), 1281 (s, C–O), 1144 (m), 1075 (m), 1007 (w) cm^{-1}; ESIMS calcd for ($C_{20}H_{18}O_6$): 354.1103; found 354.1106.

***trans*-6,7-Dimethoxy-4-[3-(naphthalen-1-yl)oxiran-2-yl]-2*H*-chromen-2-one (11c).** The Standard Procedure **2** was followed by use of Na@SiO_2 (14.7 mg, 0.640 mmol, 2.0 equiv) in dry THF (2.0 mL), 1-napthaldehyde (**1n**, 50.1 mg, 0.321 mmol, 1.0 equiv), and 4-(chloromethyl)-6,7-dimethoxy-2*H*-chromen-2-one (**10c**, 97.9 mg, 0.385 mmol, 1.2 equiv). After the reaction mixture was stirred at 25 °C for 30 min, the residue was purified by use of column chromatography (13% EtOAc in hexanes as the eluent) to give the *trans*-epoxide **11c** (0.109 g, 0.289 mmol) exclusively in 91% yield as white solids: mp (recrystallized from methanol/CH_2Cl_2) 139.2–139.4 °C; ^{1}H NMR ($CDCl_3$, 400 MHz) δ 7.93–7.91 (m, 2 H, 2 × ArH), 7.88 (d, J = 8.0 Hz, 1 H, ArH), 7.65 (d, J = 6.8 Hz, 1 H, H-5'), 7.55–7.48 (m, 3 H, 3 × ArH), 6.90 (s, 1 H, ArH), 6.89 (s, 1 H, H-8'), 6.50 (s, 1 H, H-3'), 4.47 (d, J = 1.2 Hz, 1 H, OCHPh), 4.02 (d, J = 1.2 Hz, 1 H, OCHC=C), 3.95 (s, 3 H, OCH_3), 3.55 (s, 3 H, OCH_3); ^{13}C NMR ($CDCl_3$, 100 MHz) δ 161.35 (C=O), 153.10, 150.42, 149.76, 146.44, 133.30, 131.70, 130.90,

129.08, 128.94, 126.74, 126.27, 125.68, 122.33, 122.29, 110.20, 108.36, 103.92, 100.39, 60.25, 58.02 (OCH_3), 56.40 (OCH_3), 56.18; IR (neat) 3065 (m, C=CH), 1694 (s, C=O), 1598 (s, Ar), 1495 (s, Ar), 1451 (s, Ar), 1416 (s, Ar), 1234 (s, C–O), 1007 (w), 888 (m), 759 (m) cm^{-1}; ESIMS calcd for ($C_{23}H_{18}O_5$): 374.1154; found 374.1157.

Reference

1. Mothe, S. R., Kothandaraman, P., Rao, W. & Chan, P. W. H. Rapid access to halohydrofurans via brønsted acid-catalyzed hydroxylation/halocyclization of cyclopropyl methanols with water and electrophilic halides. *J. Org. Chem.* **76**, 2521–2531 (2011).
2. Liao, Y. X., Xing, C. H. & Hu, Q. S. Rhodium(I)/diene-catalyzed addition reactions of arylborons with ketones. *Org. Lett*. **14**, 1544−1547 (2012).
3. Baciocchi, E., Mattioli, M. & Romano, R. Anodic oxidation of .alpha.-substituted p-xylenes. Electronic and stereoelectronic effects of .alpha.-substituents in the deprotonation of alkylaromatic radical cations. *J. Org. Chem.* **56**, 7154–7160 (1991).
4. Albrecht, M. *et al.* Hierarchical Assembly of Helicate-Type Dinuclear Titanium(IV) Complexes**.** *J. Am. Chem. Soc.* **127**, 10371−10387 (2005).
5. Nedelcu, D., Liu, J., Xu, Y., Jao, C. & Salic, A. Oxysterol binding to the extracellular domain of Smoothened in Hedgehog signaling. *Nat. Chem. Biol.* **9**, 557–564 (2013).
6. Netz, N. & Opatz, T. A modular formal total synthesis of (±)-Cycloclavine. *J. Org. Chem.* **81**, 1723−1730 (2016).

7. Wilcke, D. & Bach, T. $Sc(OTf)_3$-catalyzed diastereoselective Friedel–Crafts reactions of arenes and hetarenes with 3-phenylglycidates. *Org. Biomol. Chem.* **10**, 6498–6503 (2012).

8. Polniaszek, R. P. & Belmont, S. E. Stereospecific preparation of transglycidic esters. *Synth. Commun.* **19**, 221–232 (1989).

9. Moran-Ramallal, R., Liz, R. & Gotor, V. Enantiopure trans-3-arylaziridine-2-carboxamides: preparation by bacterial hydrolysis and ring-openings toward enantiopure, unnatural D-a-amino acids. *J. Org. Chem.* **75**, 6614–6624 (2010).

10. Imashiro, R. & Seki, M. A catalytic asymmetric synthesis of chiral glycidic acid derivatives through chiral dioxirane-mediated catalytic asymmetric epoxidation of cinnamic acid derivatives. *J. Org. Chem.* **69**, 4216–4226 (2004).

11. Li, S. W., Spaziano, V. T. & Burke, W. J. Synthesis of a biochemically important aldehyde, 3,4-dihydroxyphenylacetaldehyde. *Bioorg. Chem.* **26**, 45–50 (1998).

12. Jew, S. S. *et al.* Highly enantioselective epoxidation of 2,4-diarylenones by using dimeric cinchona phase-transfer catalysts: enhancement of enantioselectivity by surfactants. *Angew. Chem. Int. Ed.* **44**, 1383–1385 (2005).

13. Bakó, P., Makó, A., Keglevich, G., Kubinyi, M. & Pal, K. Synthesis of D-mannose-based azacrown ethers and their application in enantioselective reactions. *Tetrahedron: Asymmetry* **16**, 1861–1871 (2005).

NMR and Mass Spectra of New Compounds

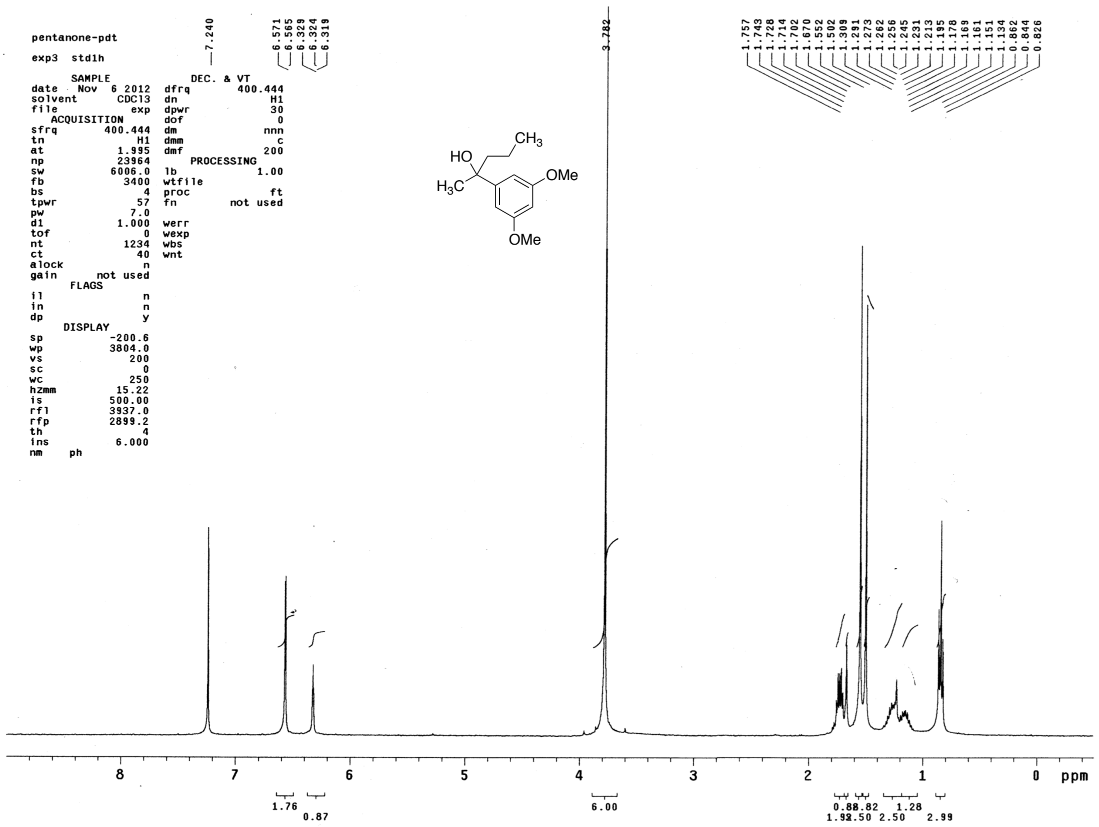

^{1}H NMR spectrum of compound **3a**

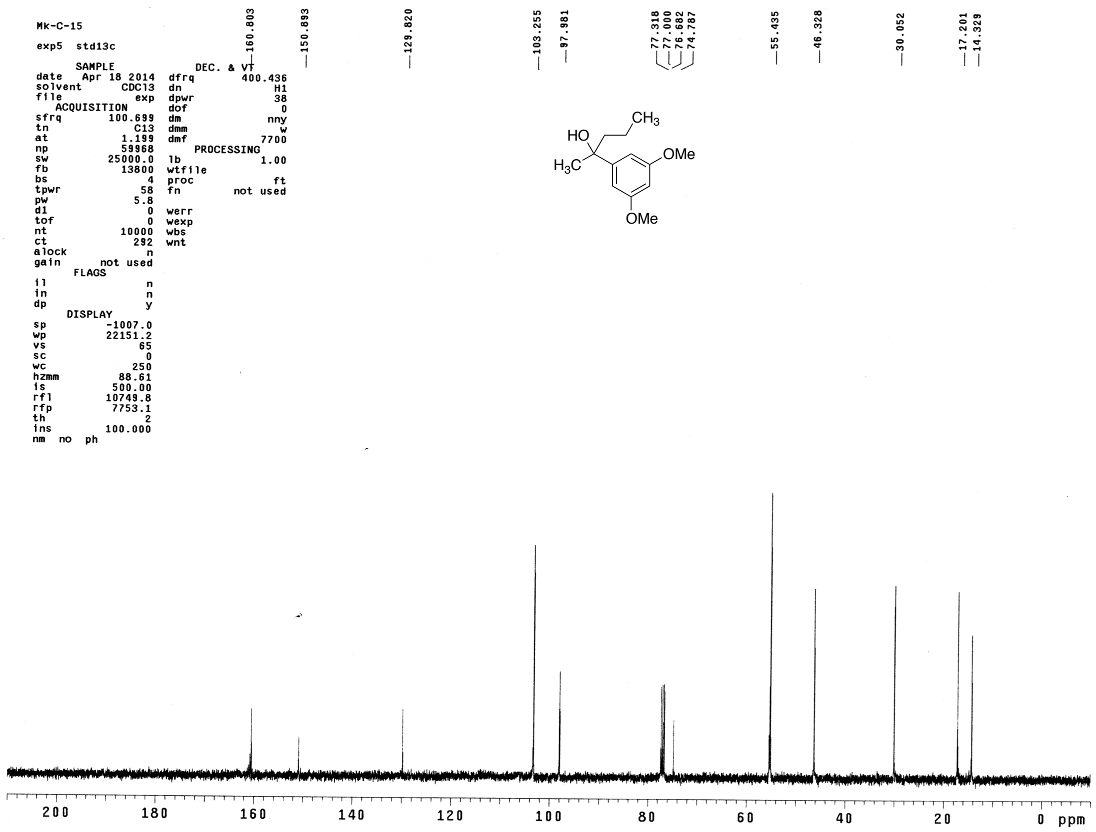

^{13}C NMR spectrum of compound **3a**

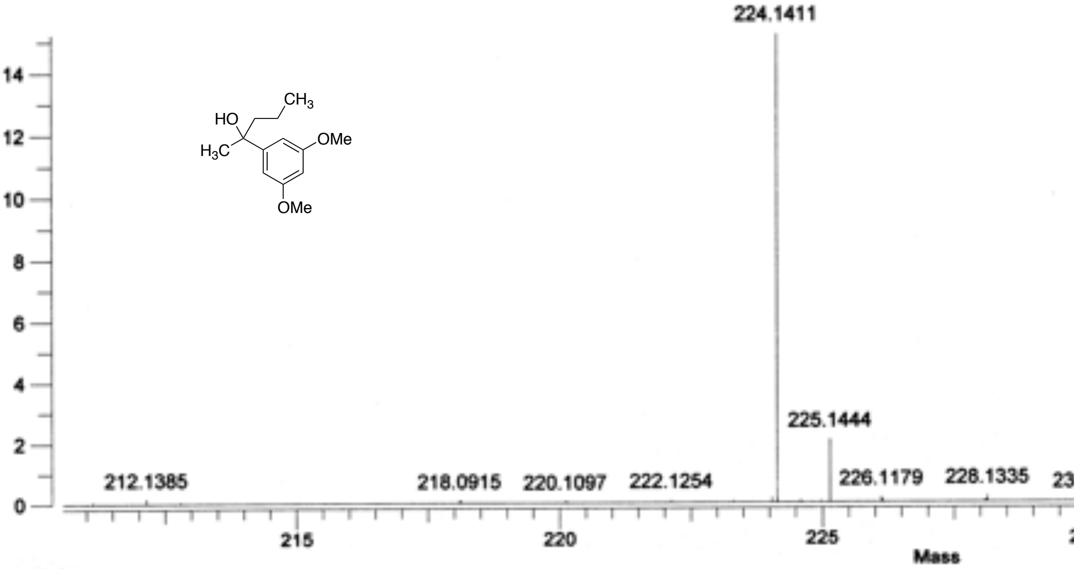

Mass spectrum of compound **3a**

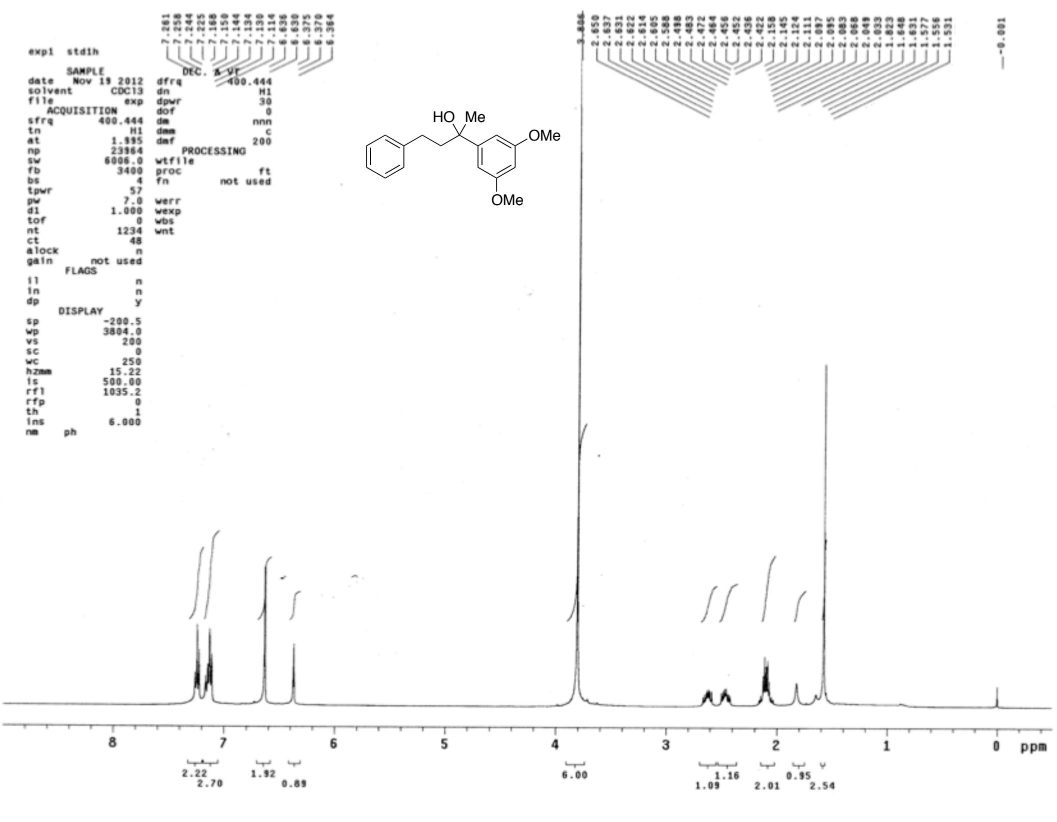

^{1}H NMR spectrum of compound **3b**

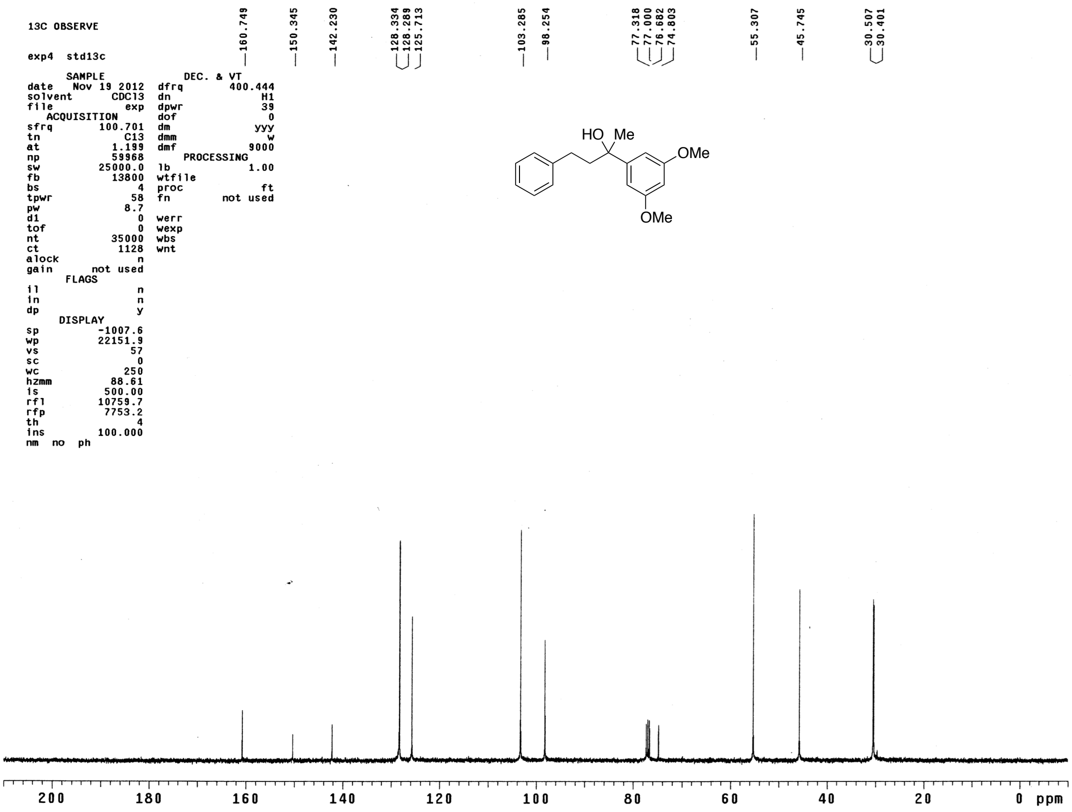

^{13}C NMR spectrum of compound **3b**

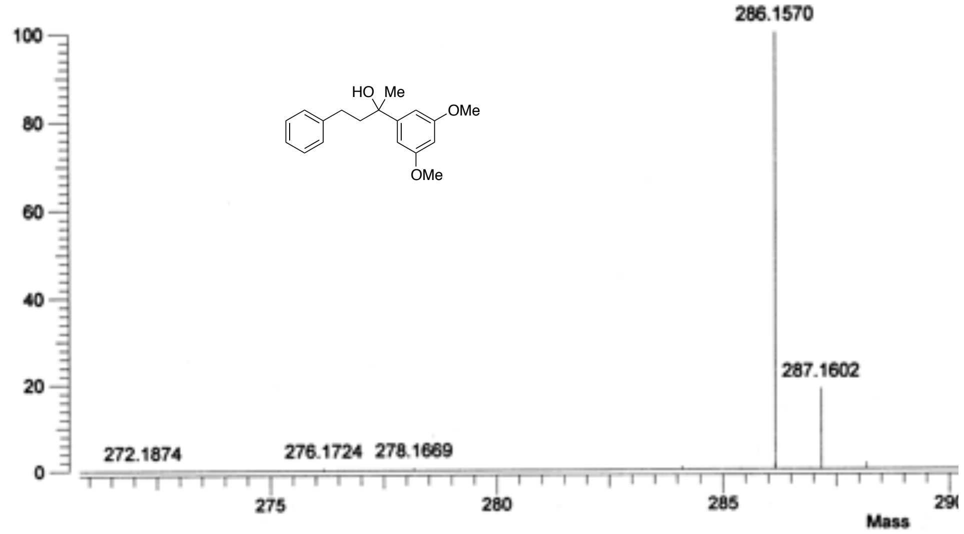

Mass spectrum of compound **3b**

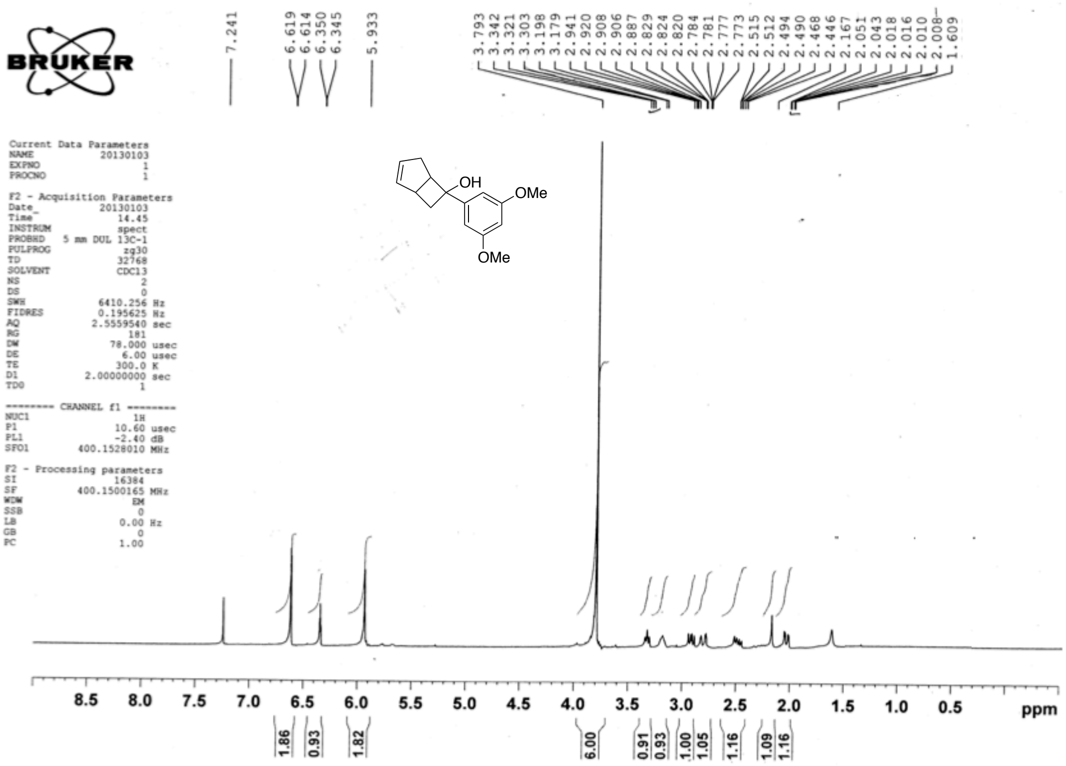

^{1}H NMR spectrum of compound **3c**

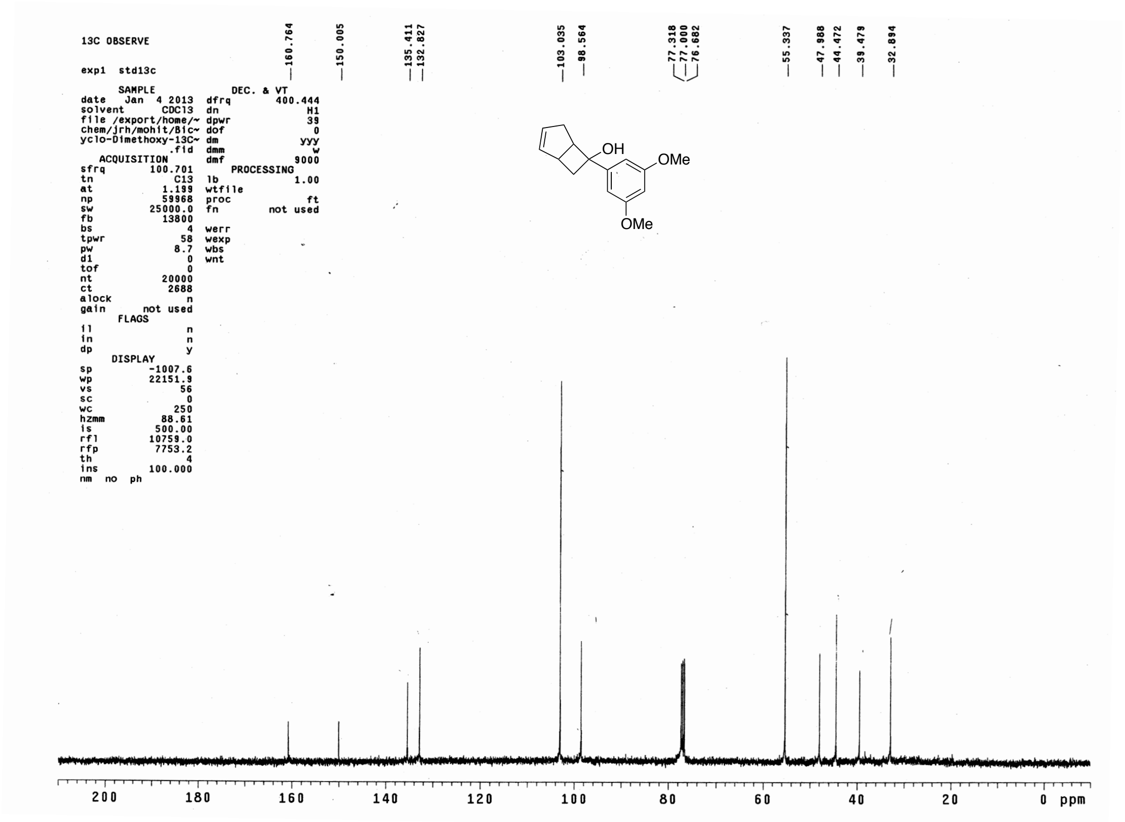

^{13}C NMR spectrum of compound **3c**

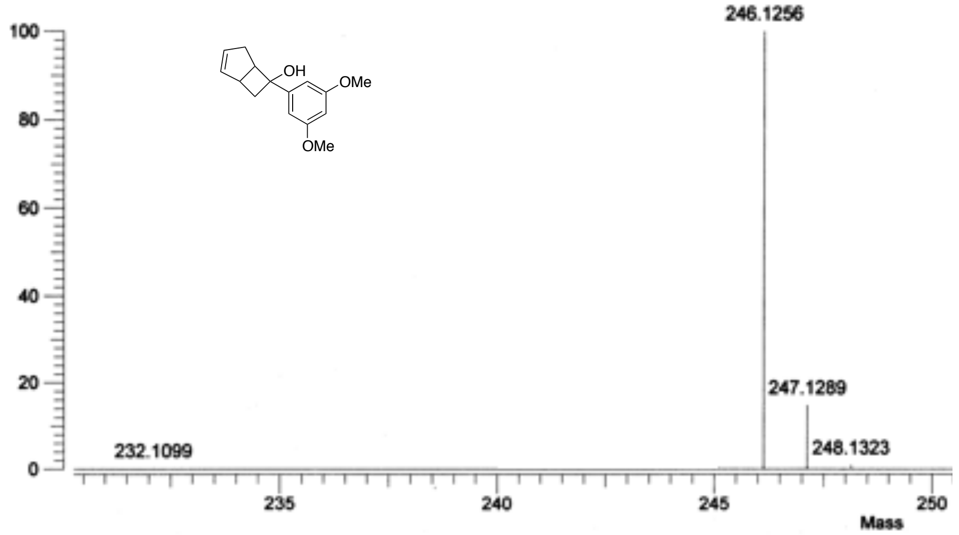

Mass spectrum of compound **3c**

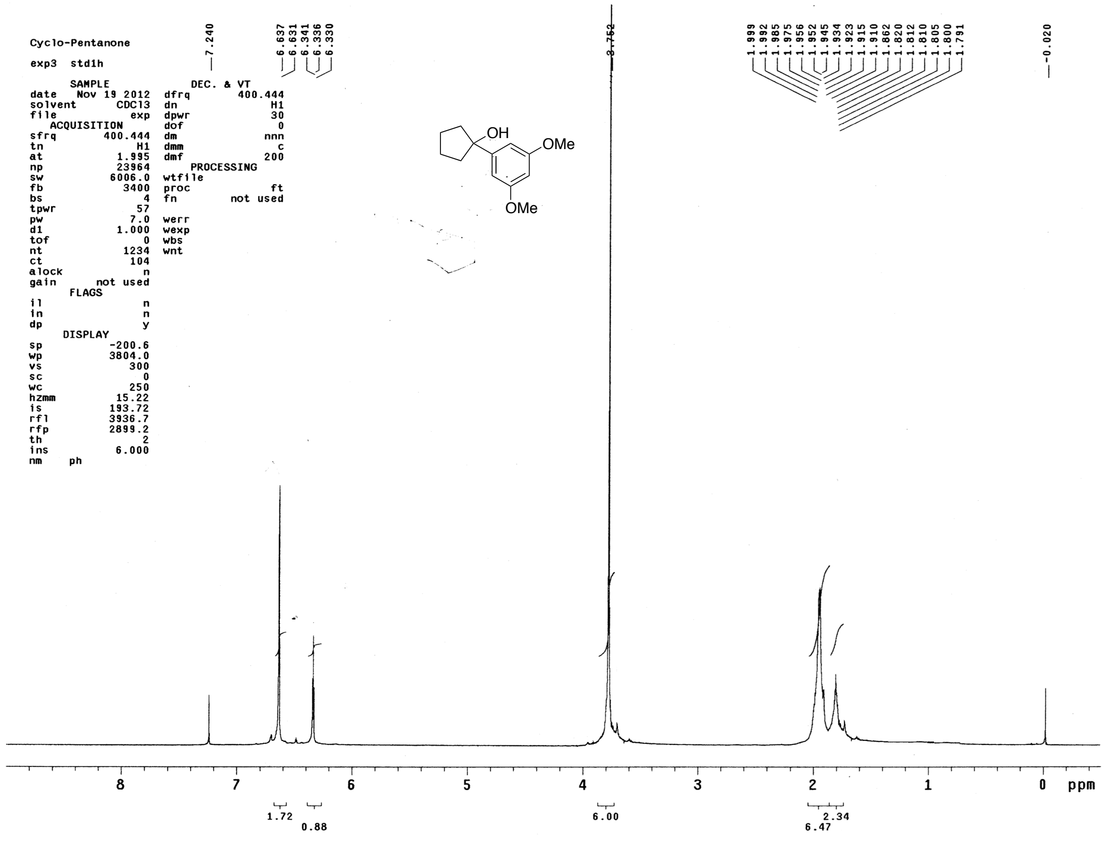

^{1}H NMR spectrum of compound **3d**

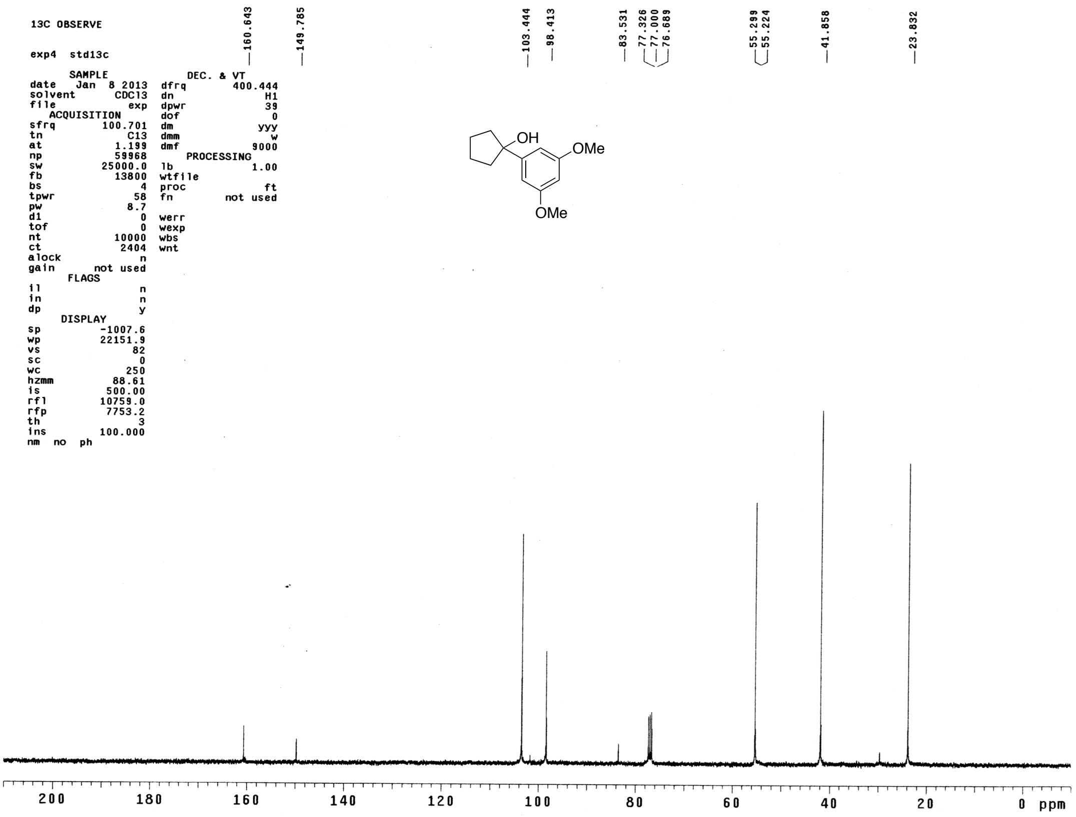

^{13}C NMR spectrum of compound **3d**

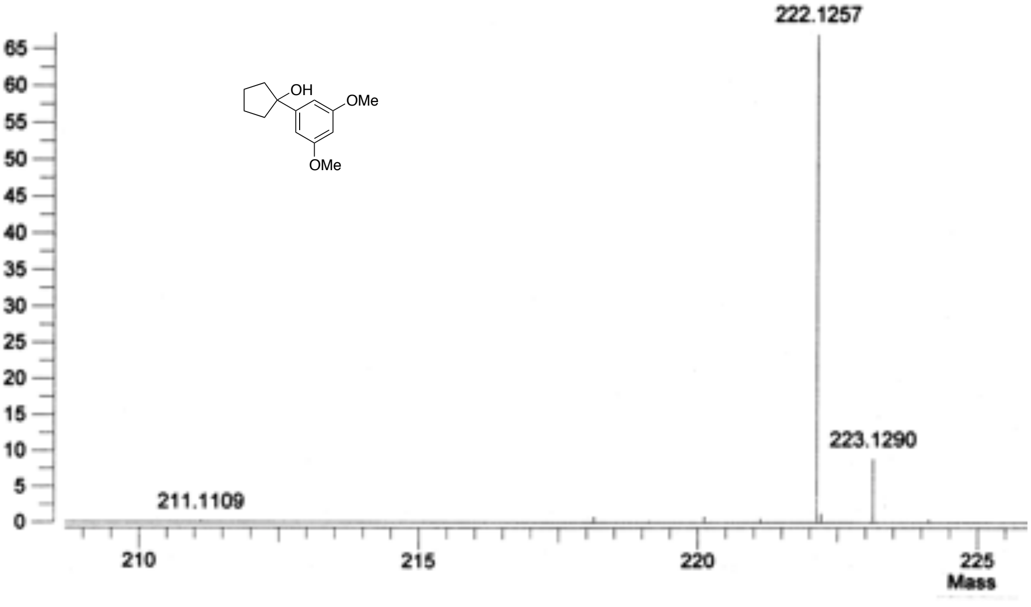

Mass spectrum of compound **3d**

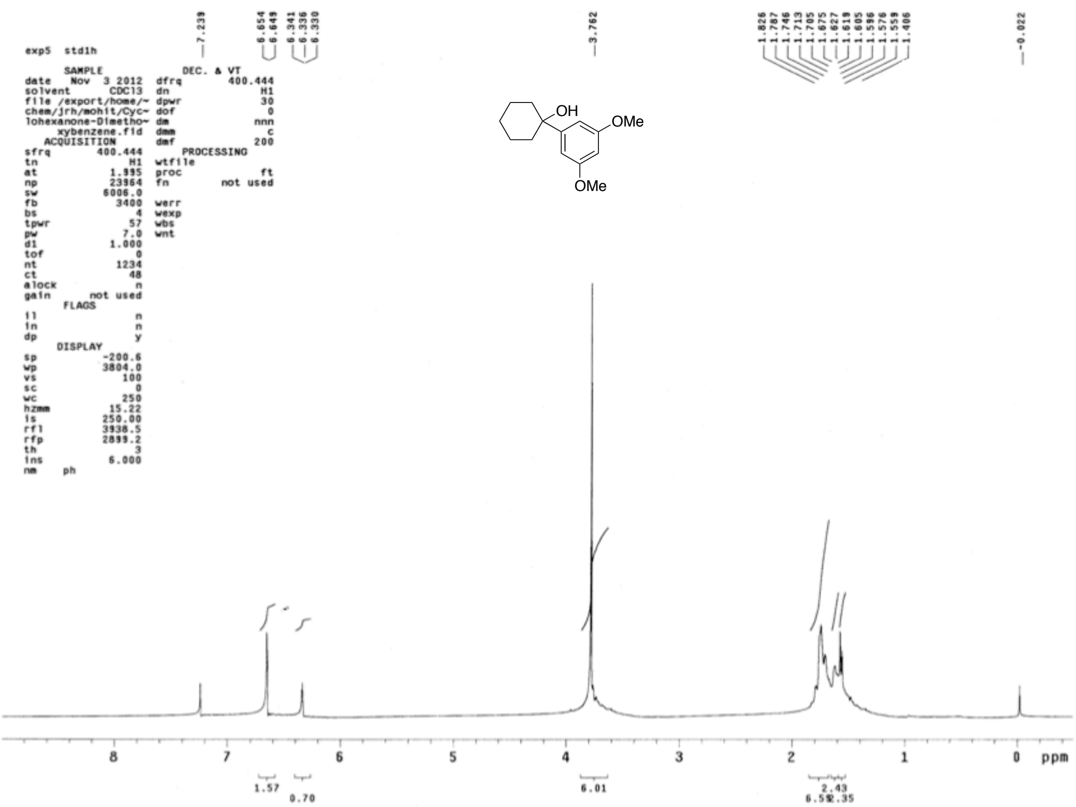

^{1}H NMR spectrum of compound **3e**

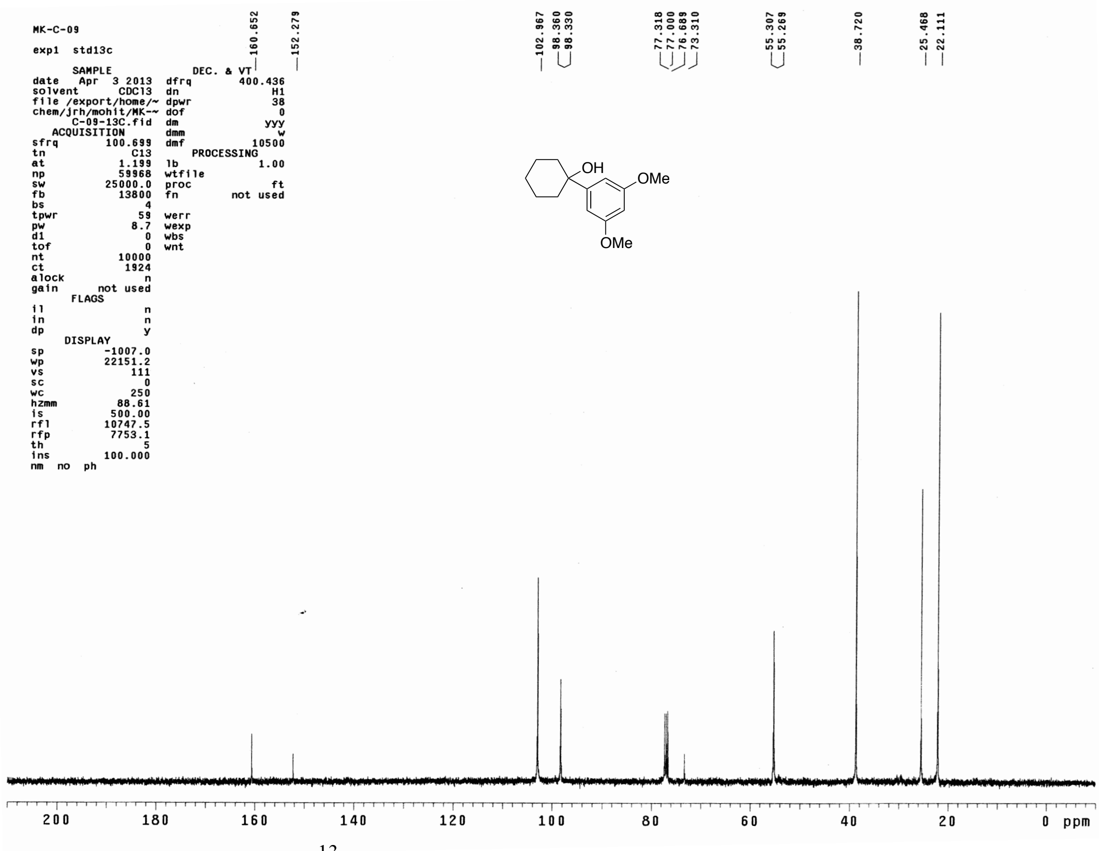

^{13}C NMR spectrum of compound **3e**

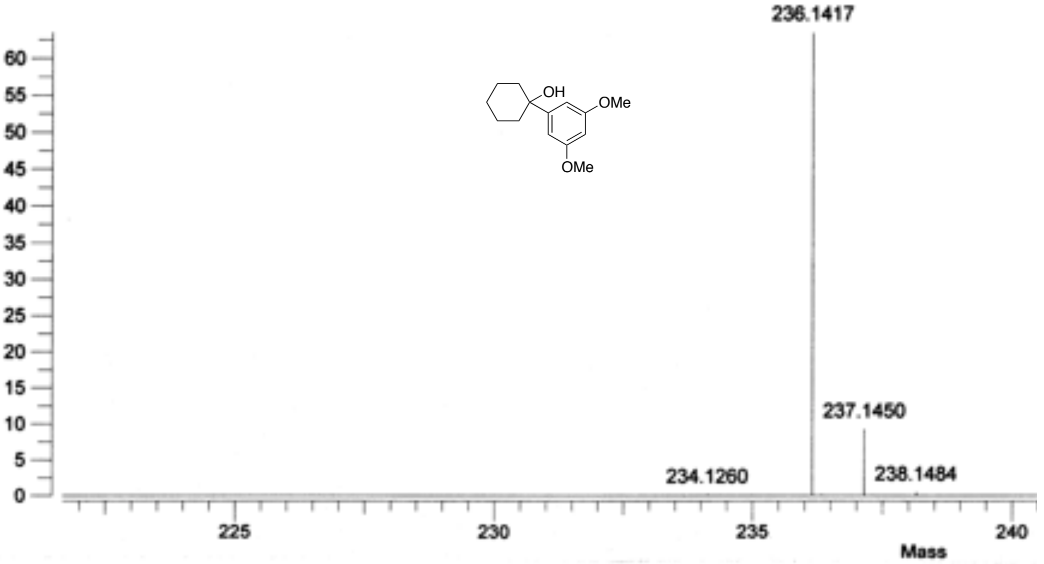

Mass spectrum of compound **3e**

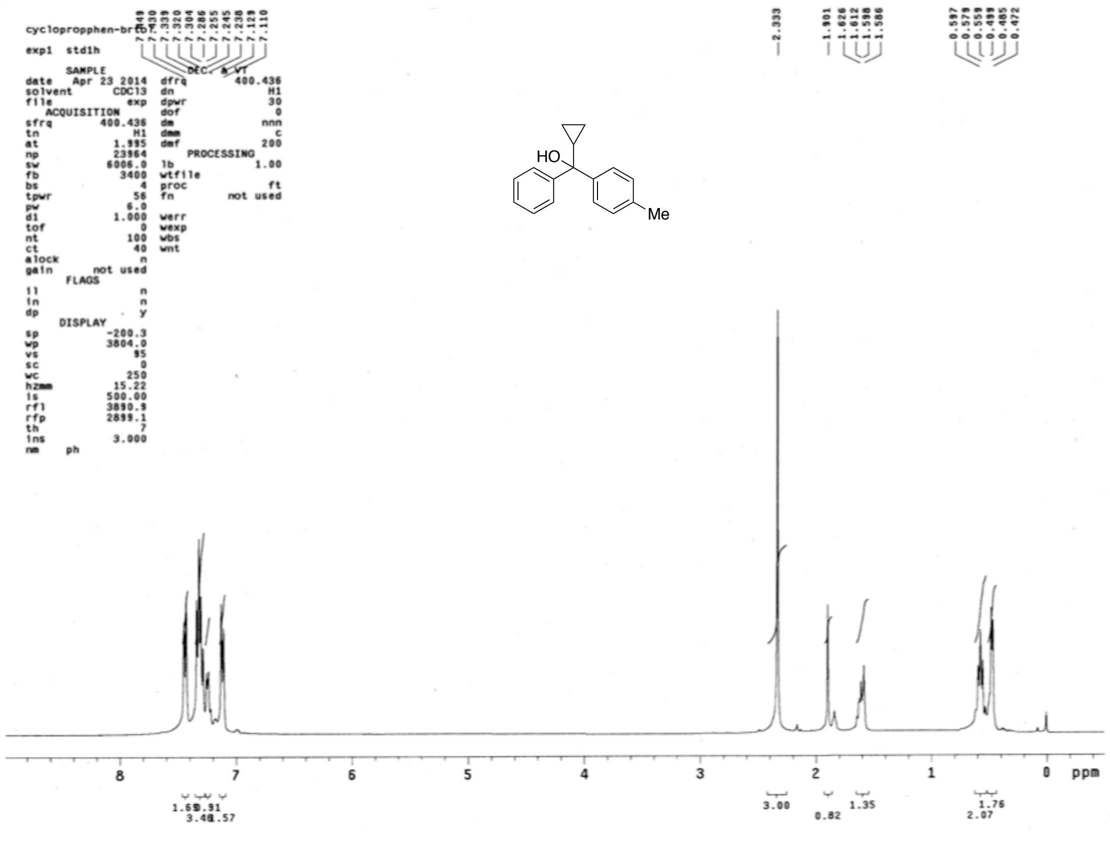

^{1}H NMR spectrum of compound **3f**

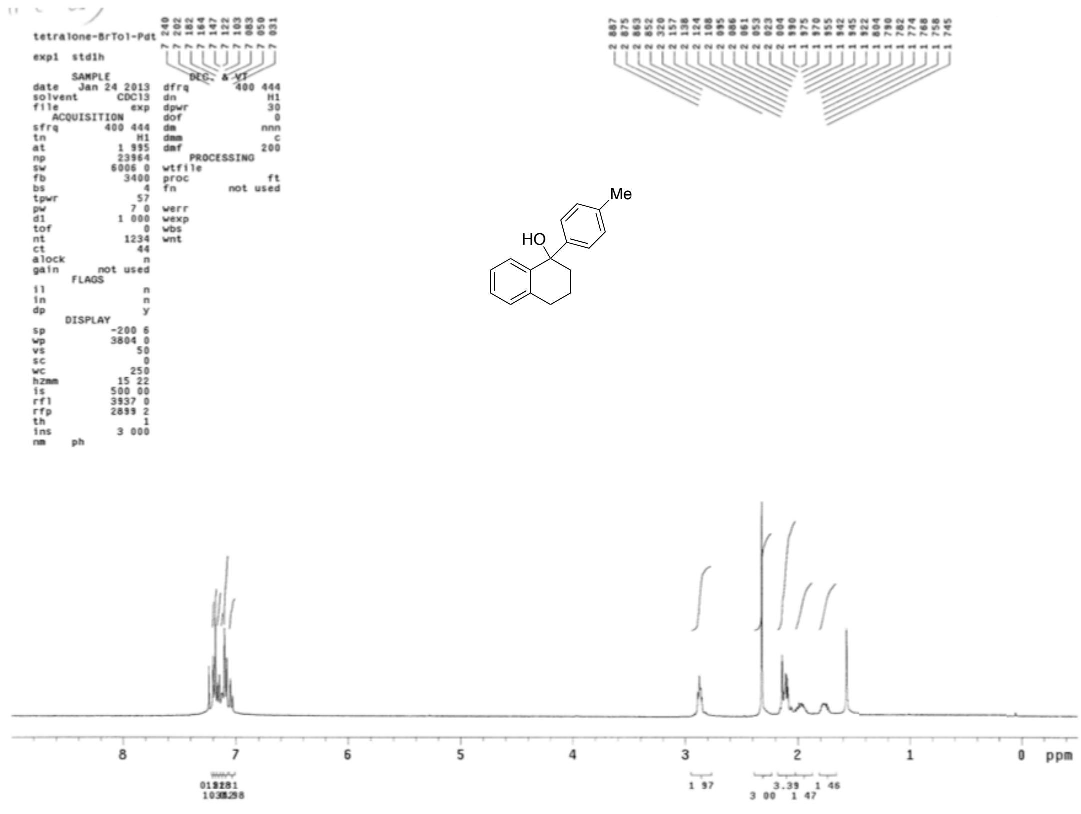

^{1}H NMR spectrum of compound **3g**

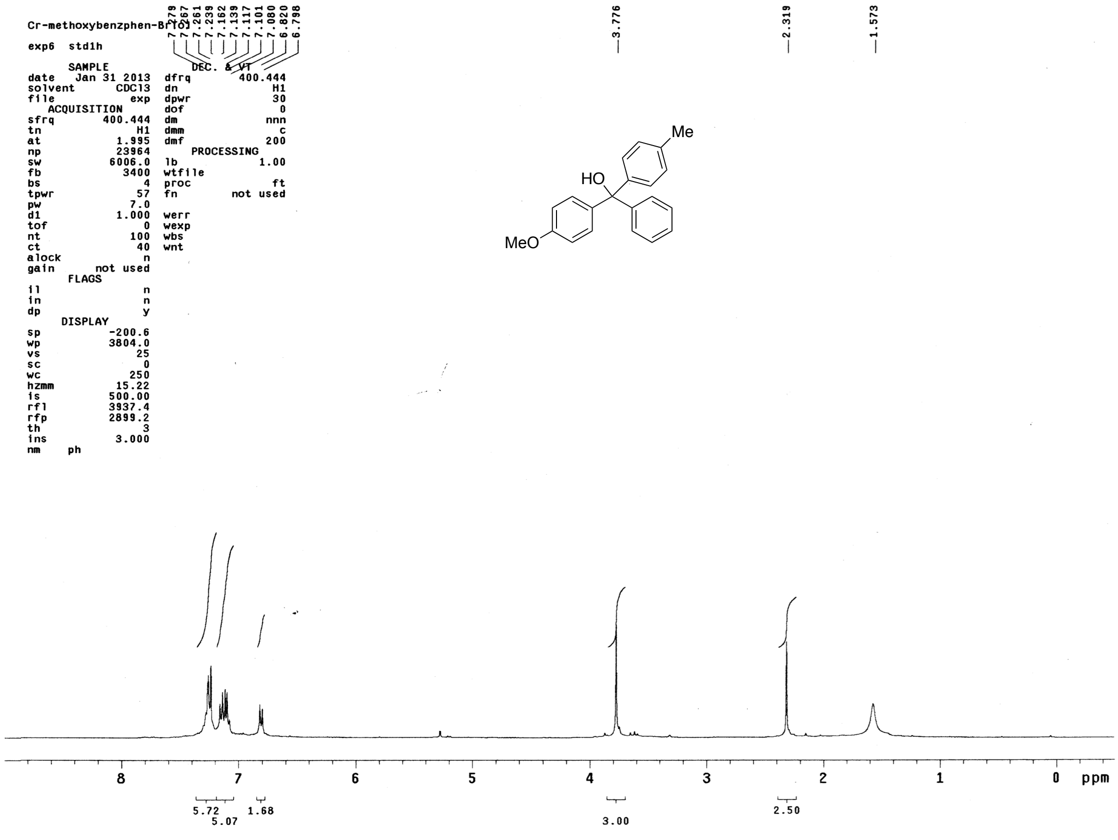

^{1}H NMR spectrum of compound **3h**

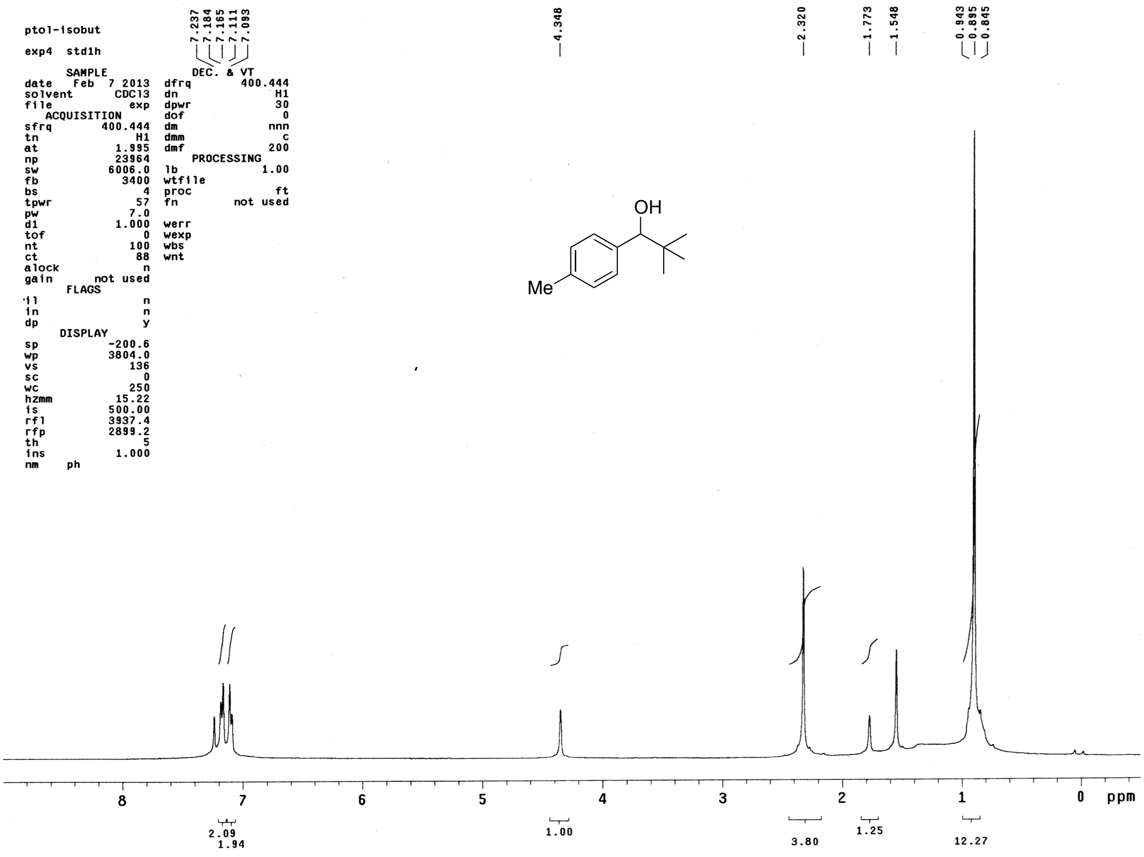

^{1}H NMR spectrum of compound **3i**

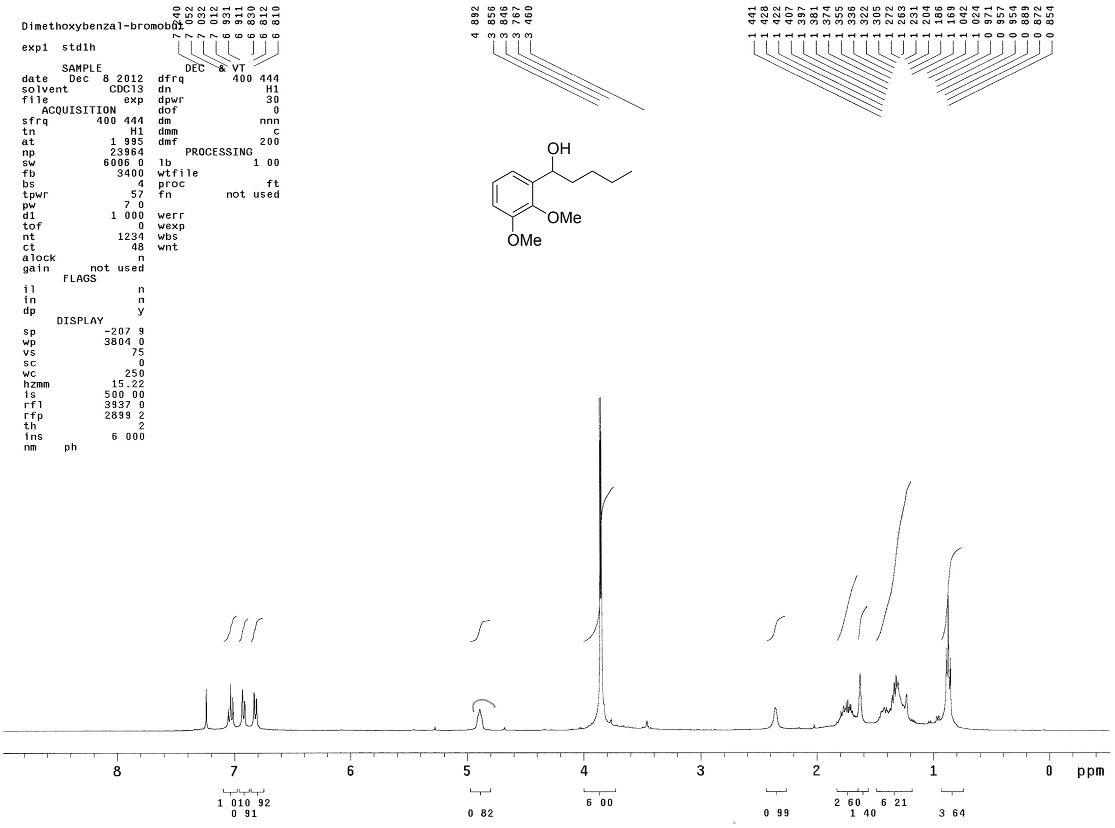

^{1}H NMR spectrum of compound **3j**

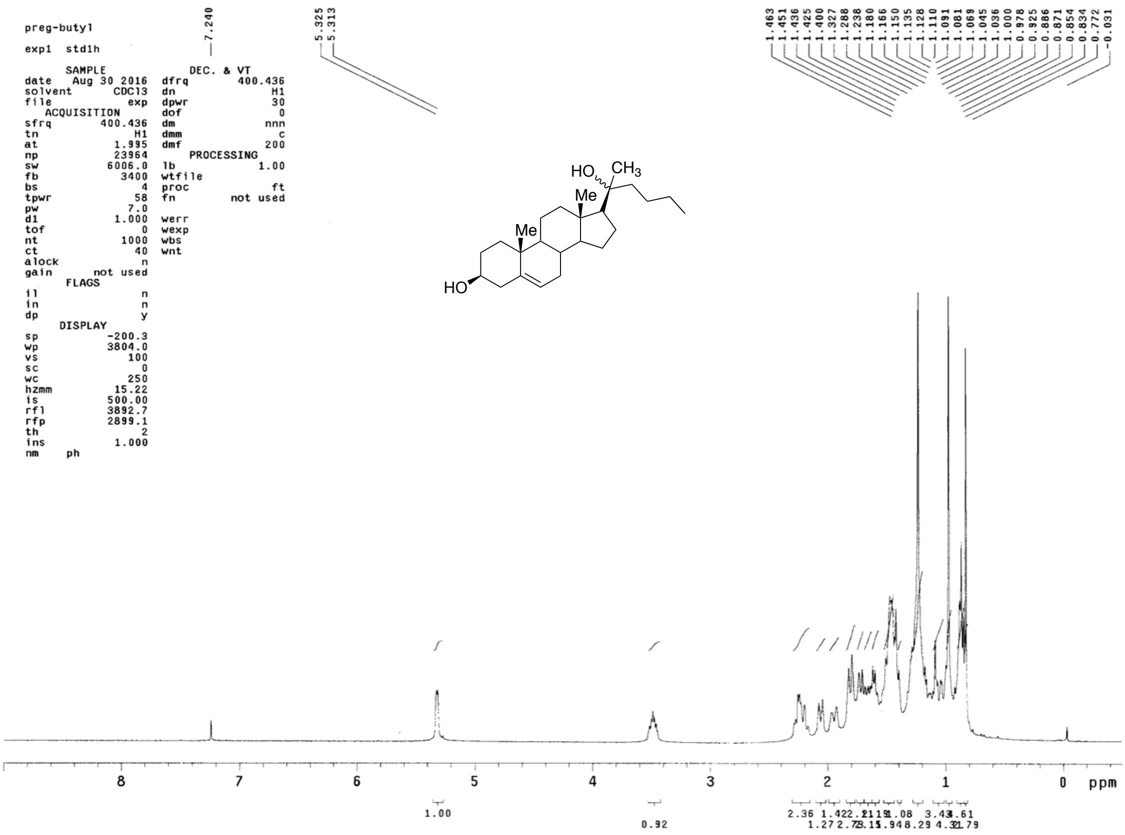

^{1}H NMR spectrum of compound **3k**

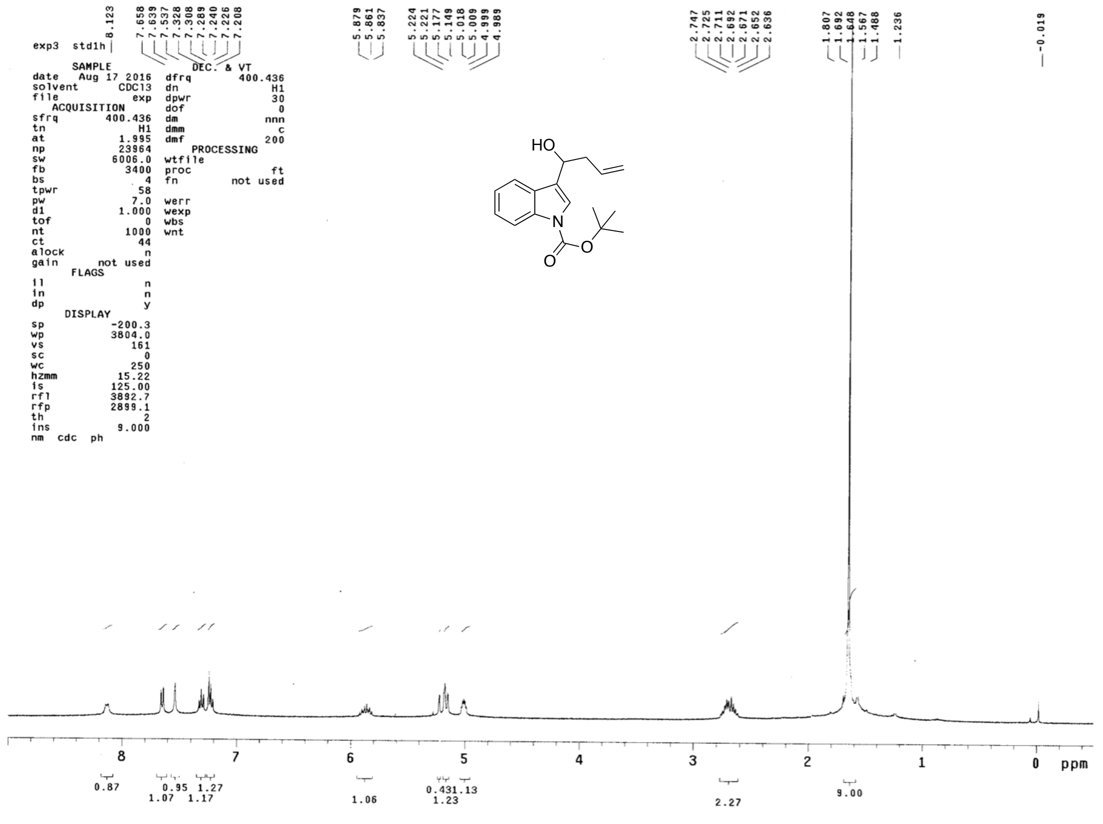

^{1}H NMR spectrum of compound **3l**

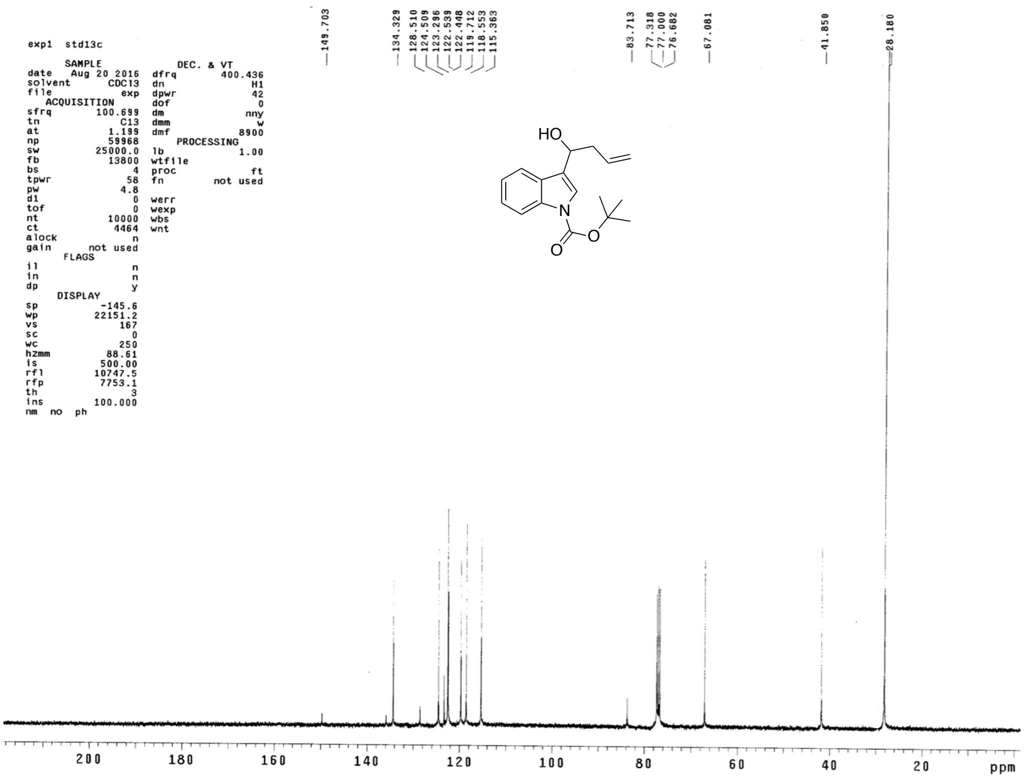

^{13}C NMR spectrum of compound **3l**

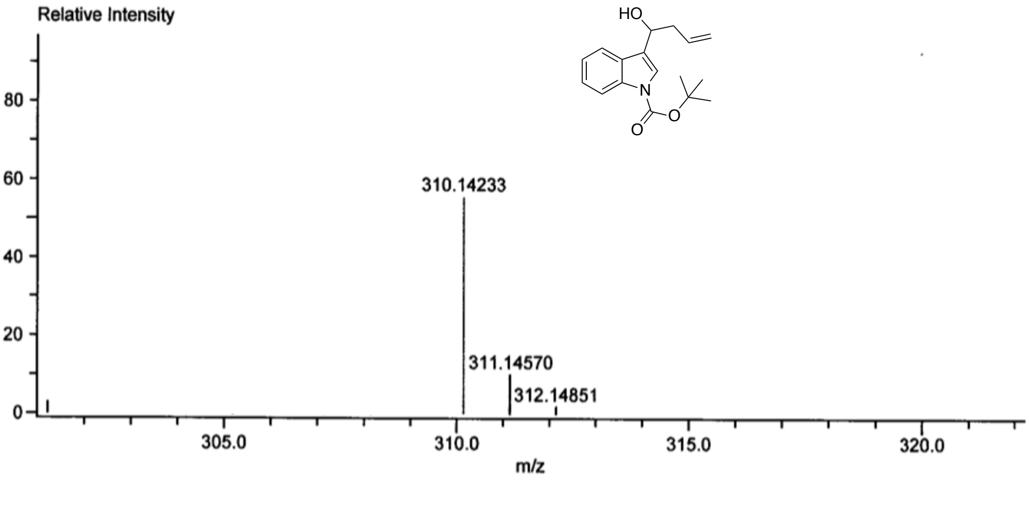

Mass spectrum of compound **3l**

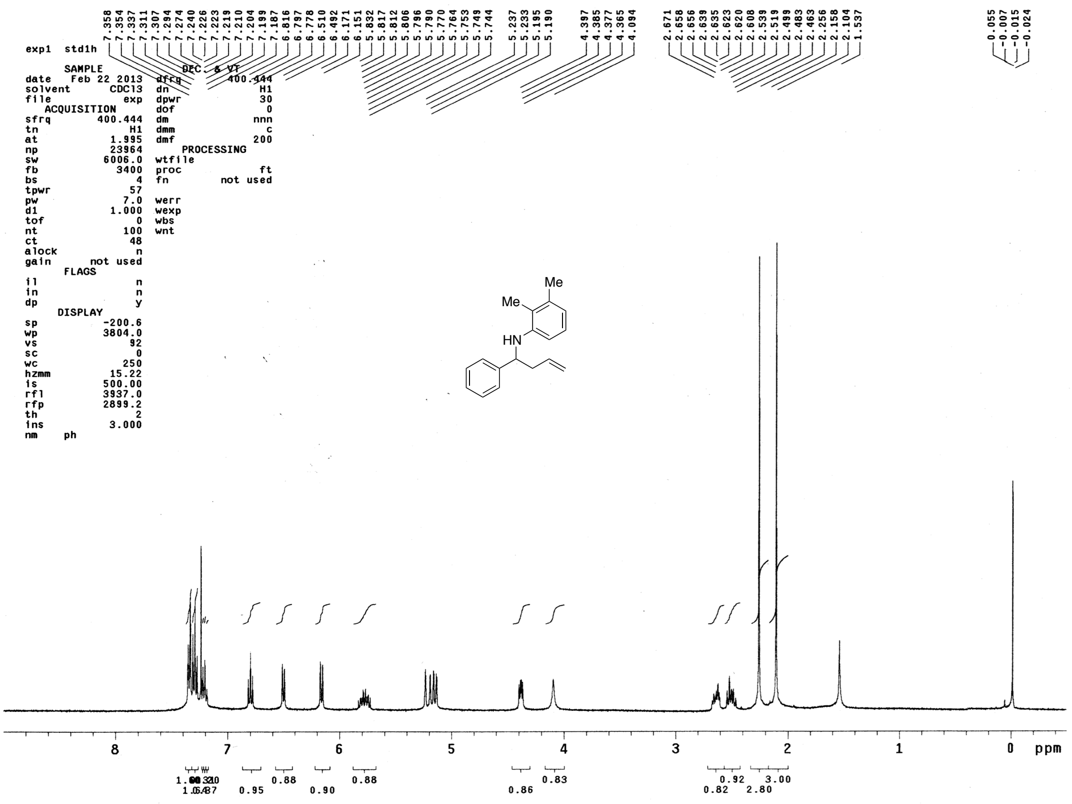

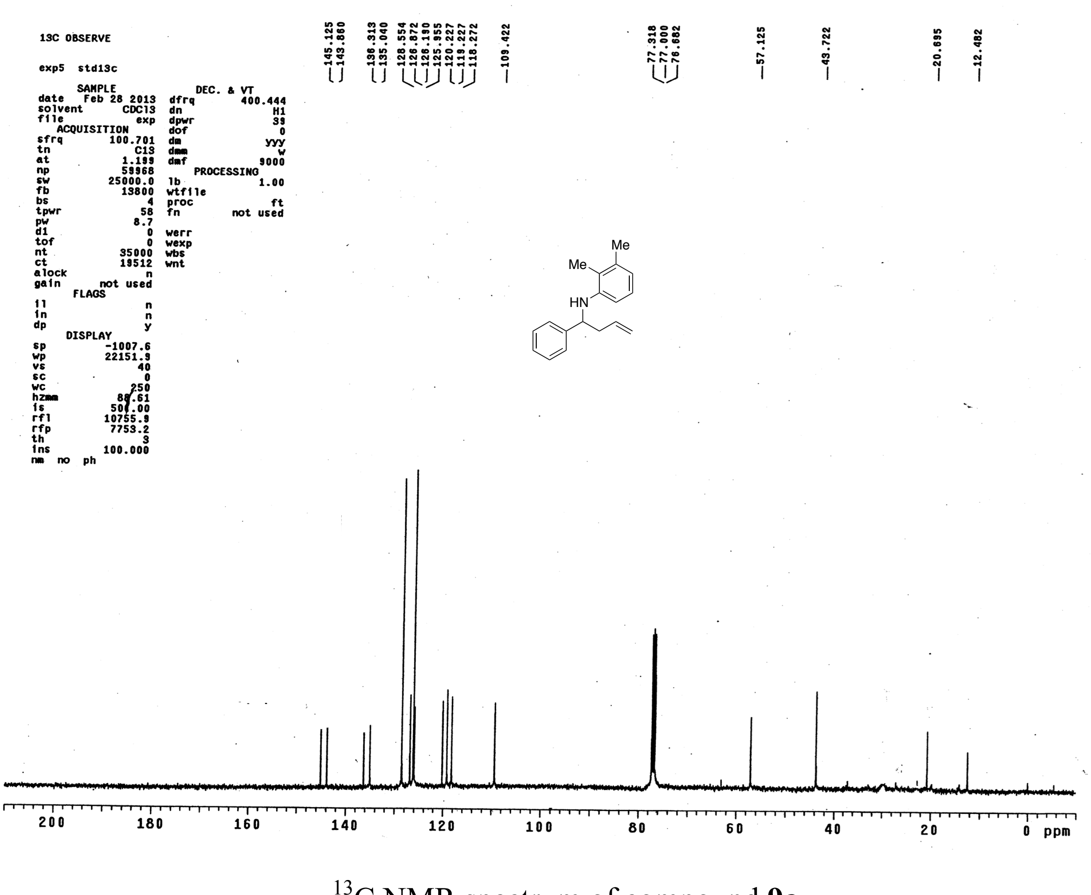

^{13}C NMR spectrum of compound **9a**

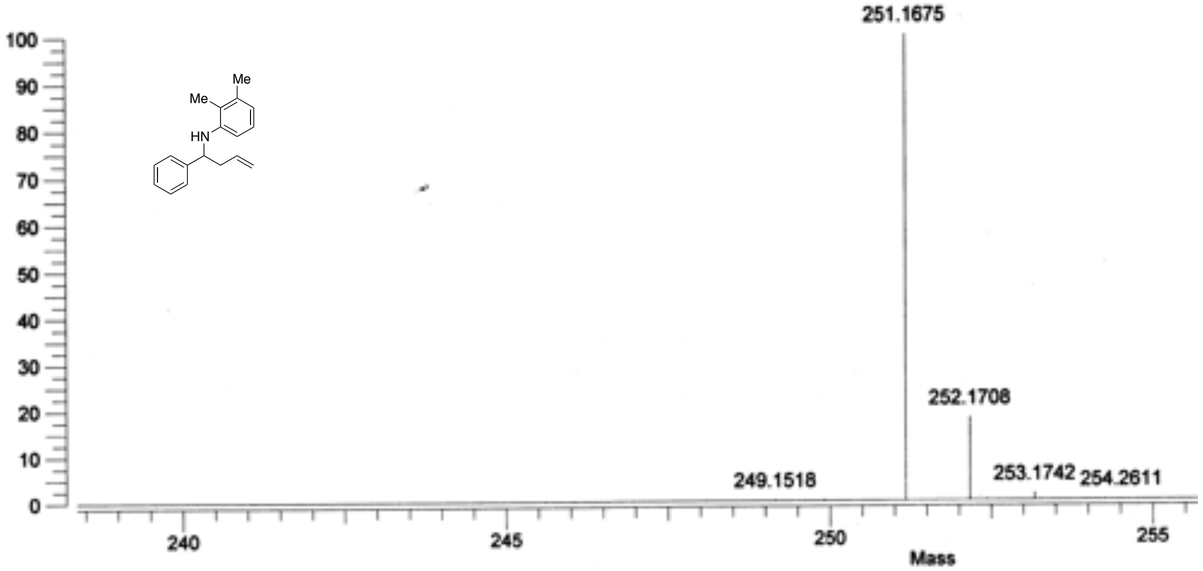

Mass spectrum of compound **9a**

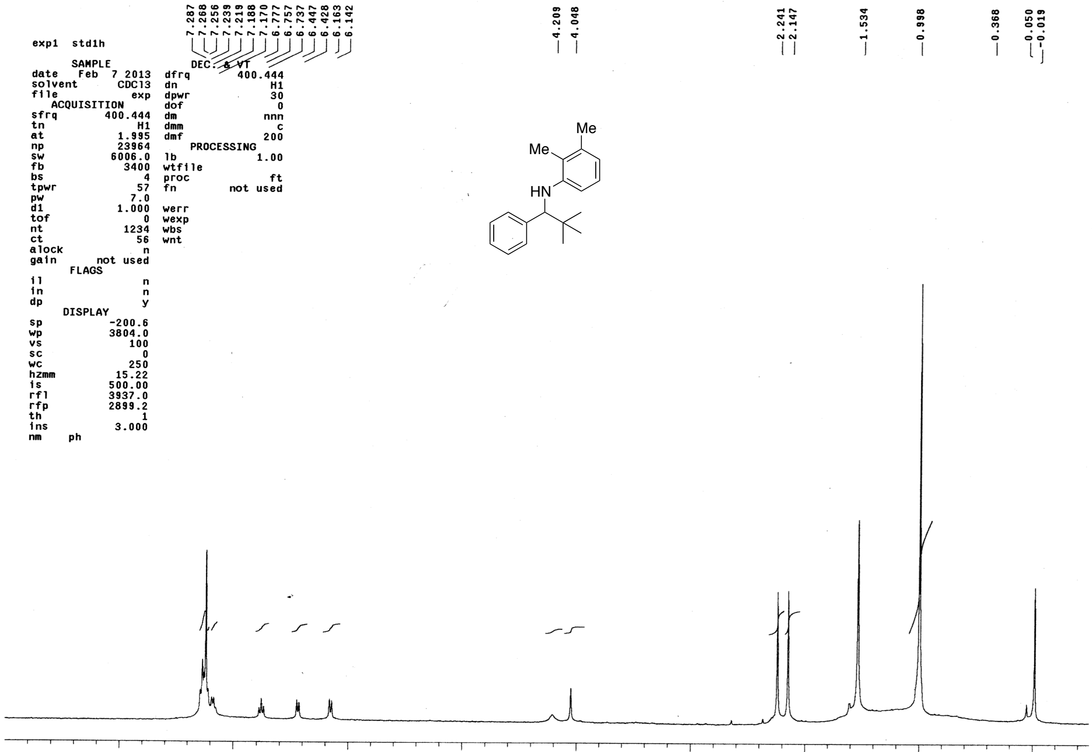

^{1}H NMR spectrum of compound **9b**

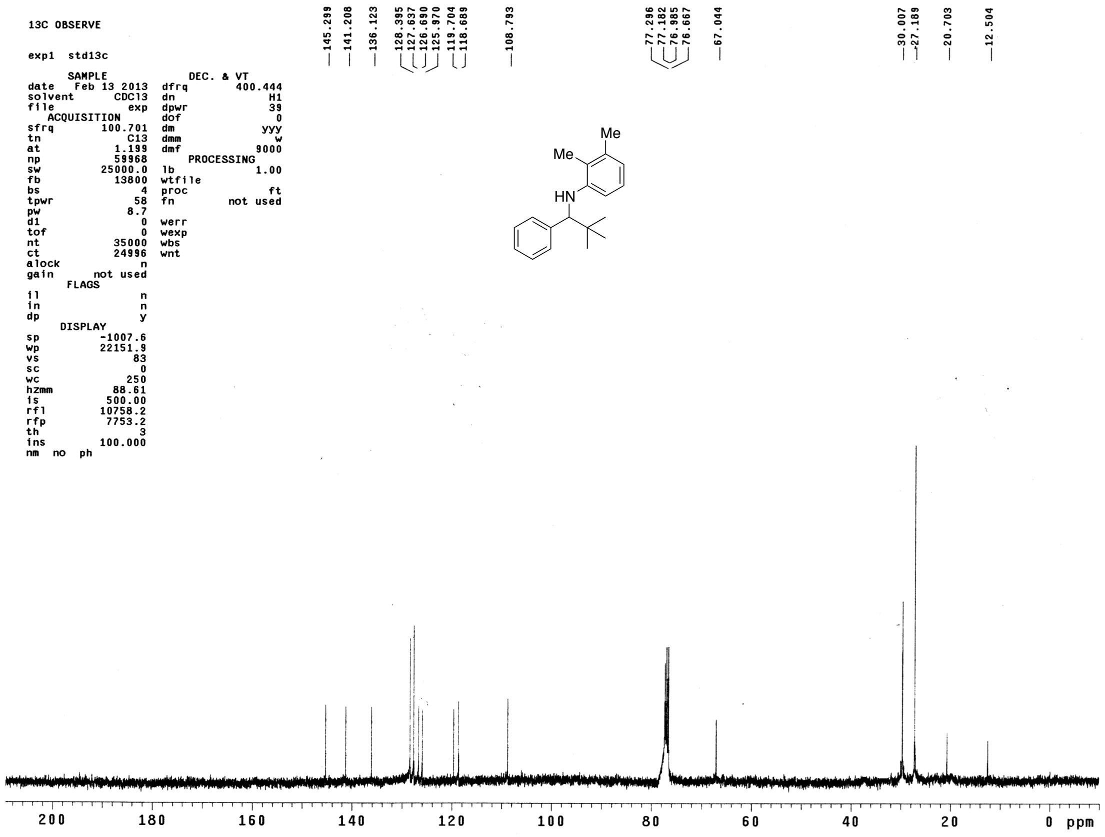

^{13}C NMR spectrum of compound **9b**

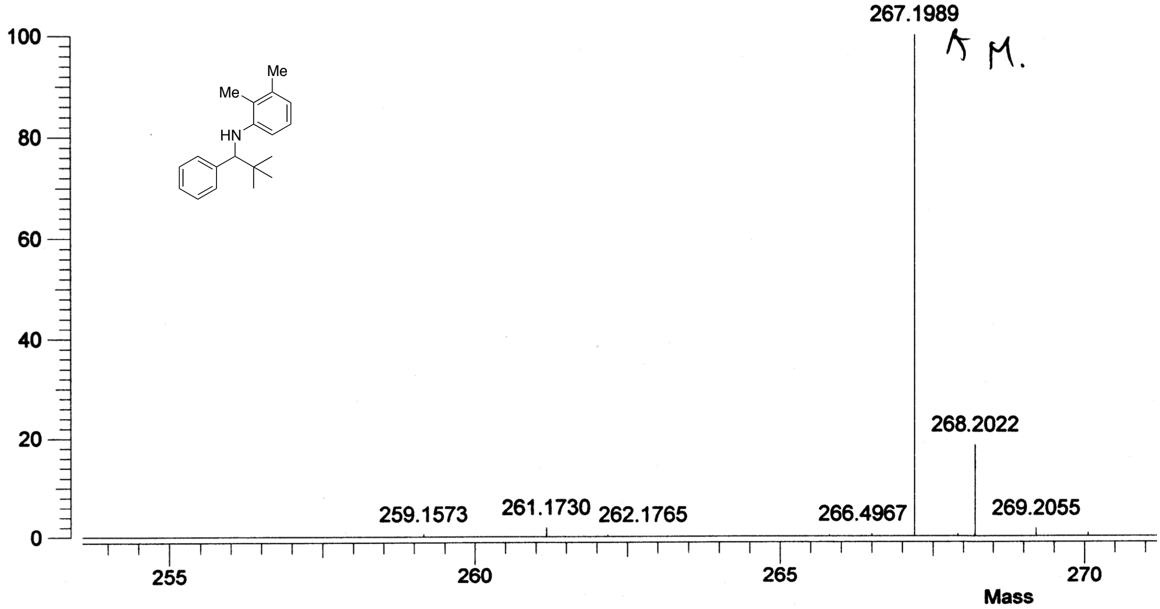

Mass spectrum of compound **9b**

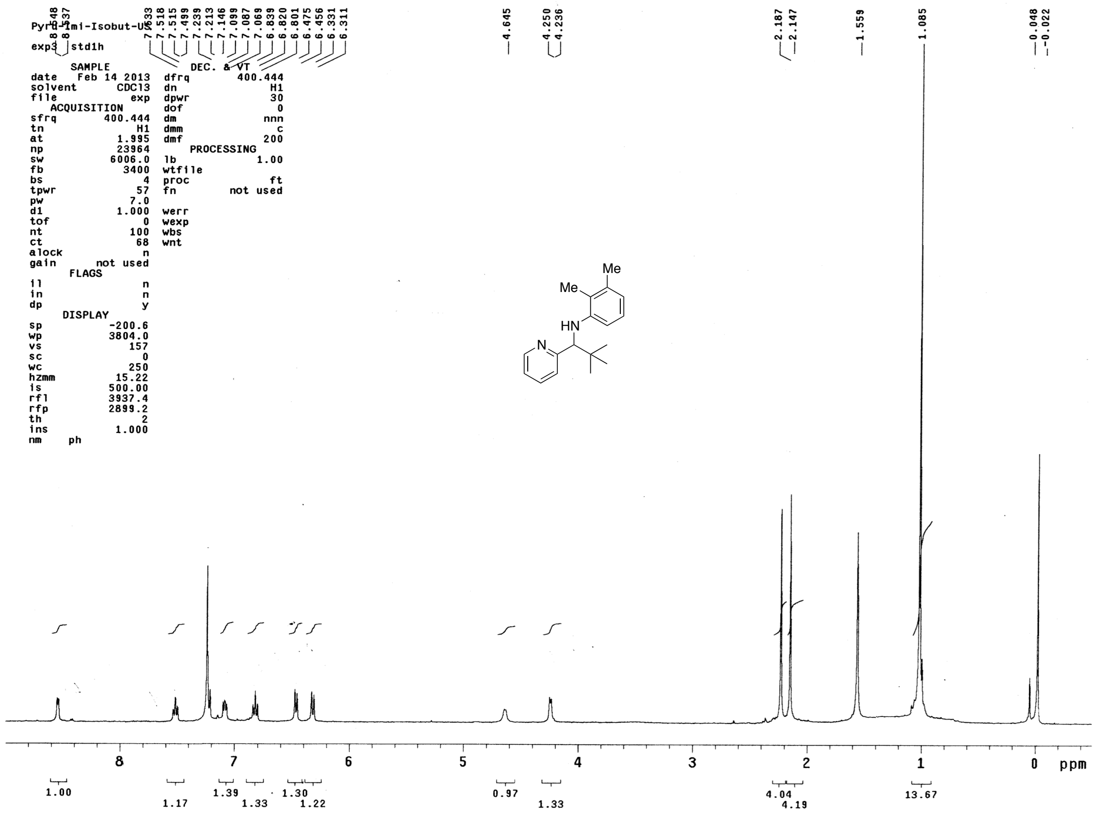

^{1}H NMR spectrum of compound **9c**

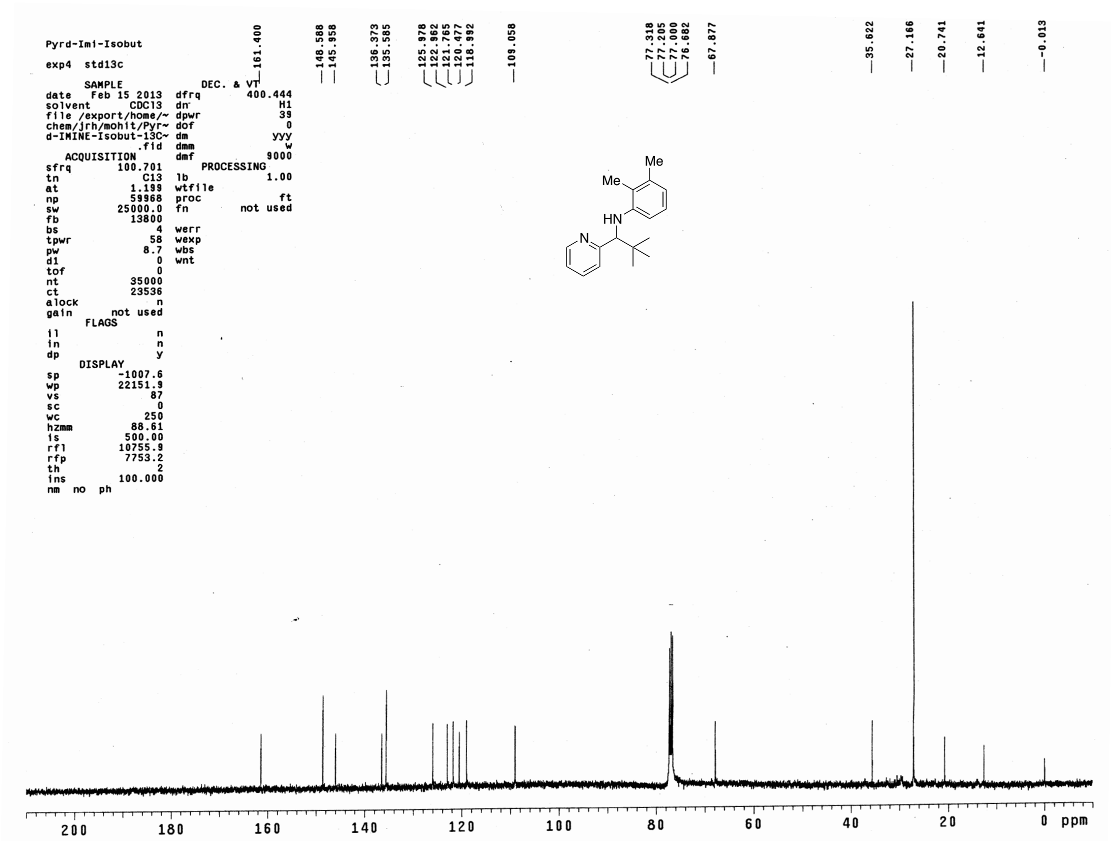

^{13}C NMR spectrum of compound **9c**

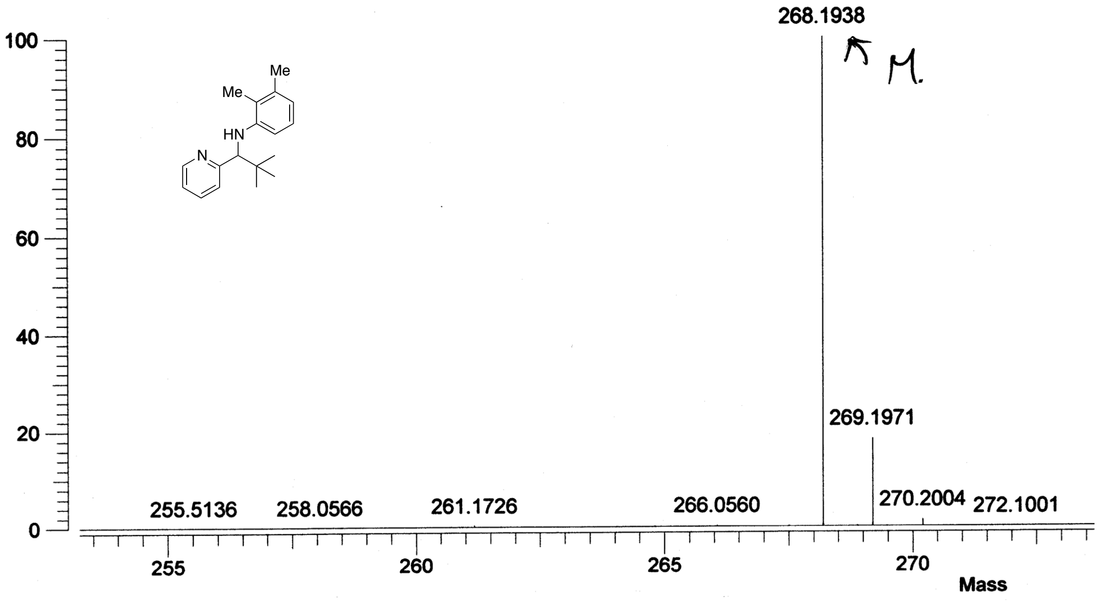

Mass spectrum of compound **9c**

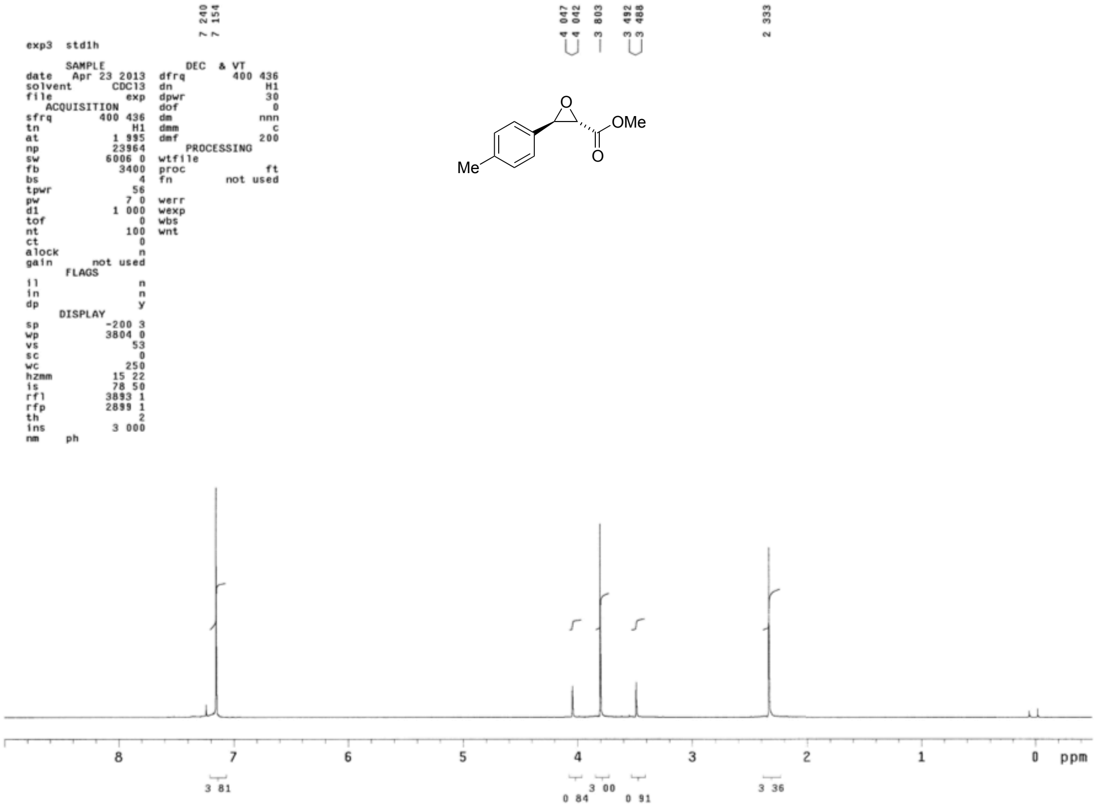

^{1}H NMR spectrum of compound **5i**

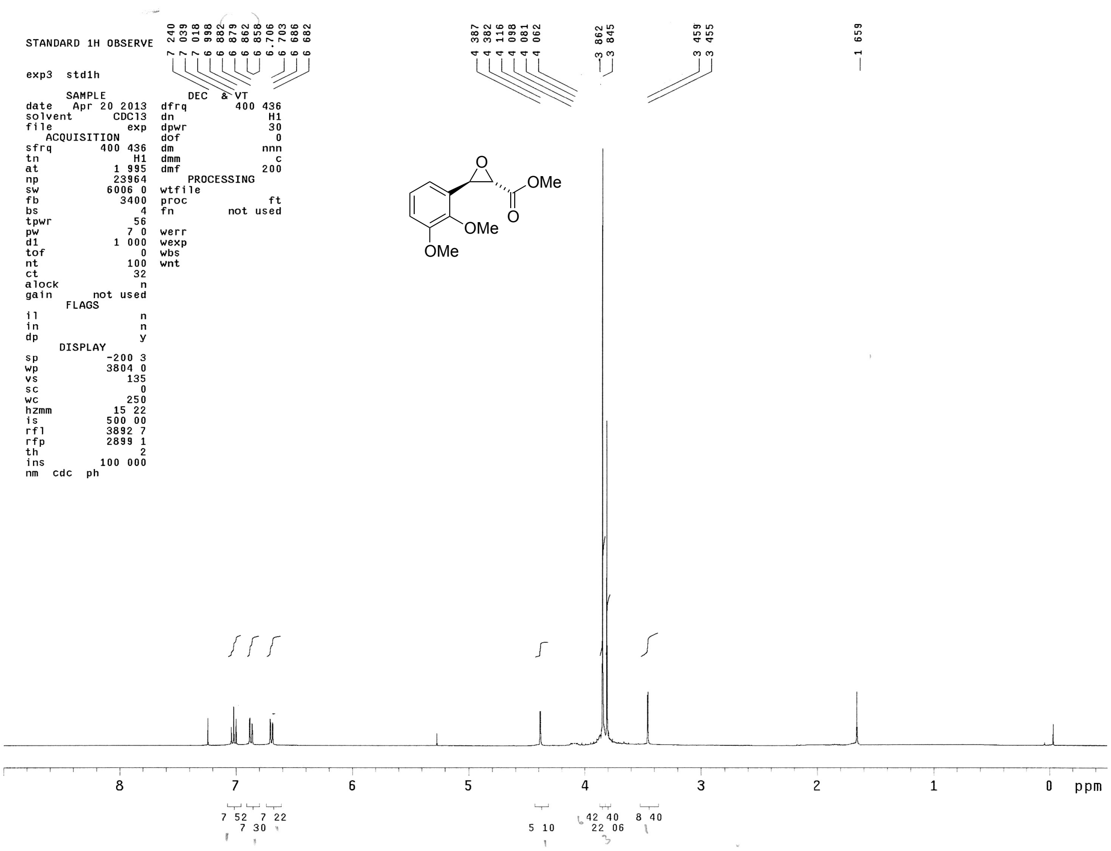

^{1}H NMR spectrum of compound **5j**

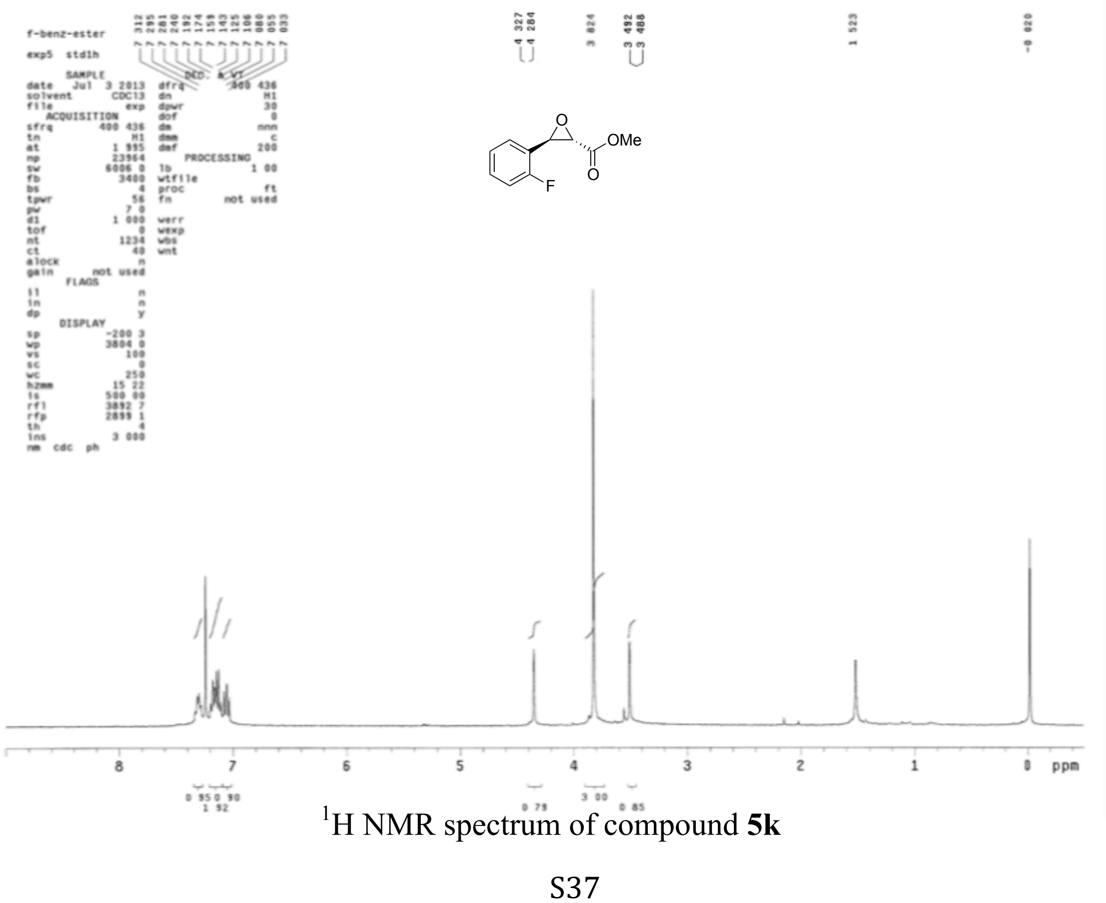

^{1}H NMR spectrum of compound **5k**

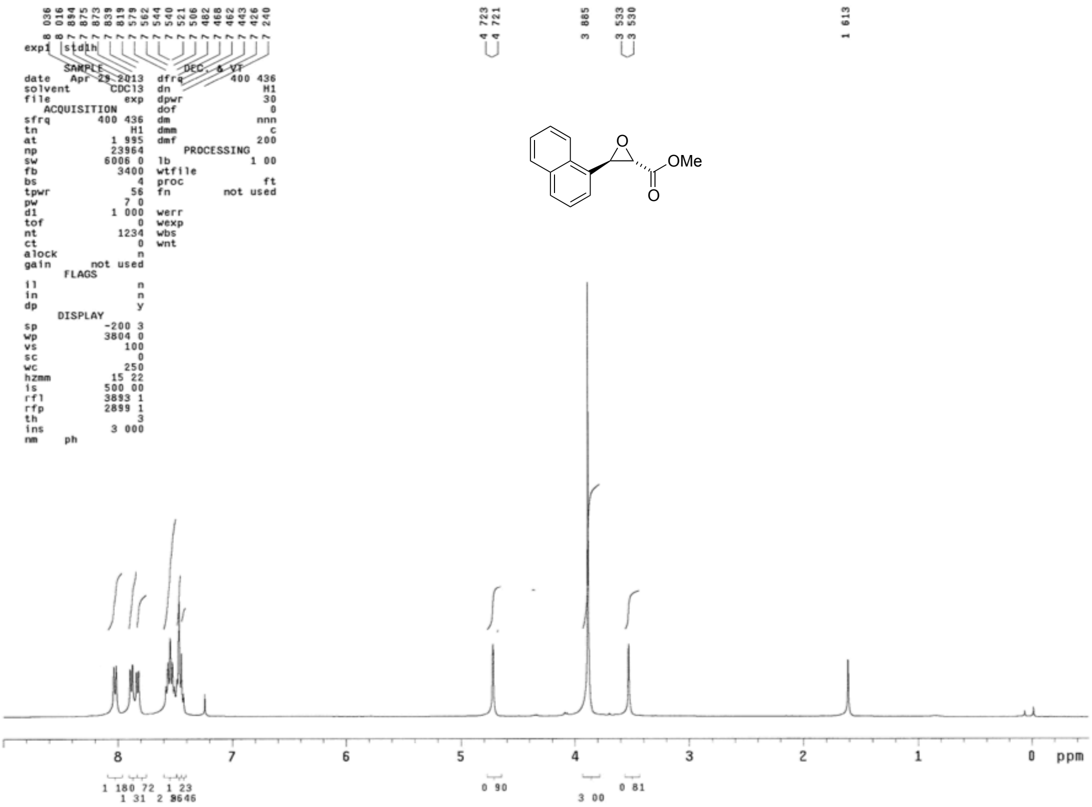

^{1}H NMR spectrum of compound **5l**

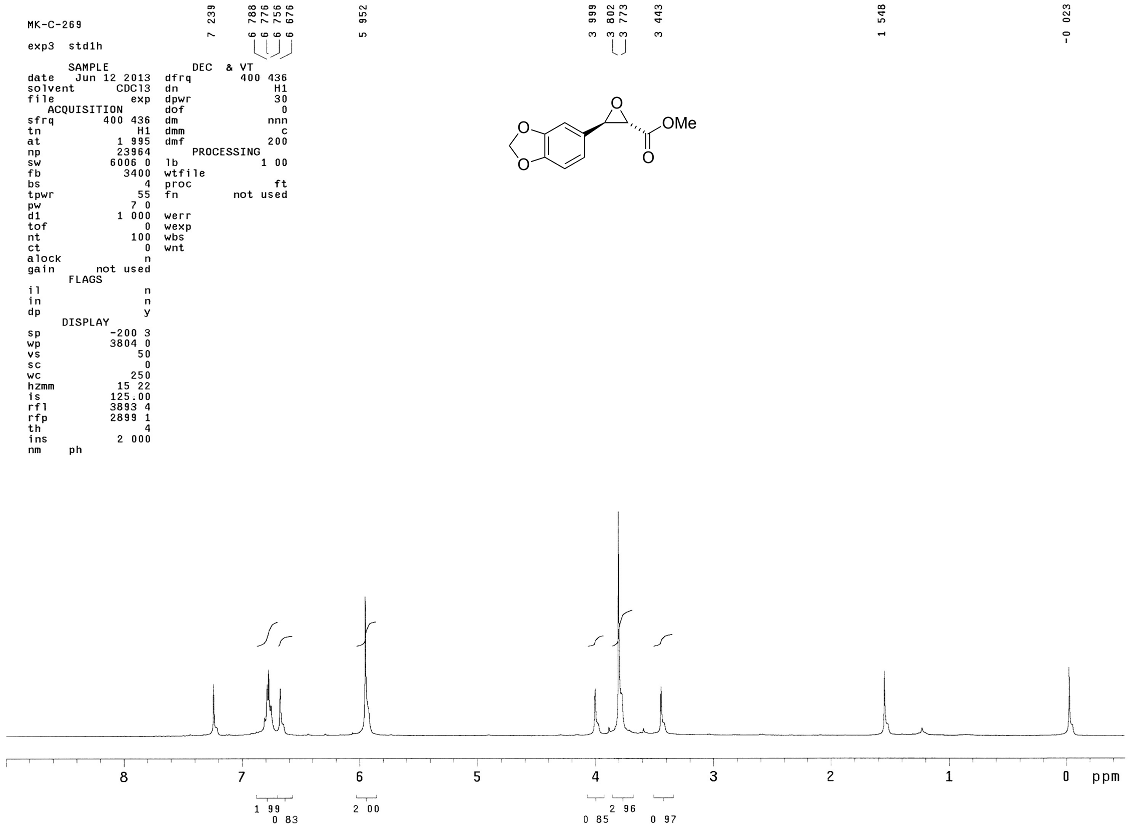

^{1}H NMR spectrum of compound **5m**

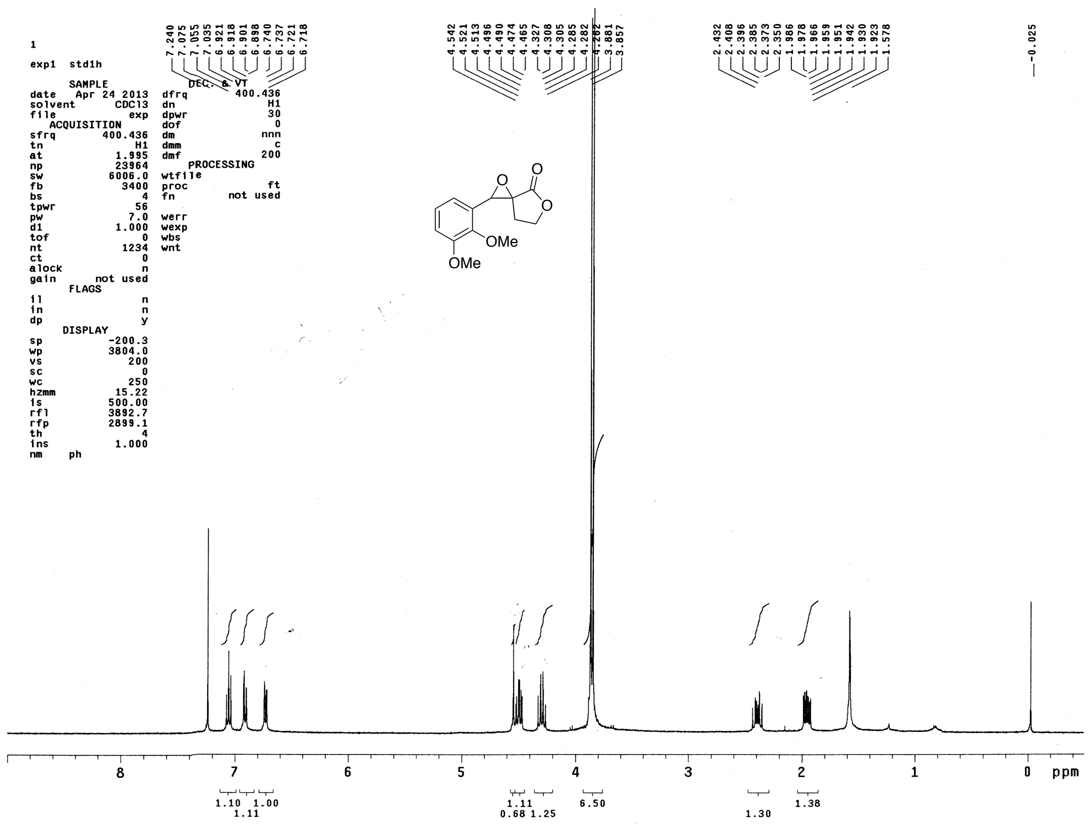

^{1}H NMR spectrum of compound **5n**

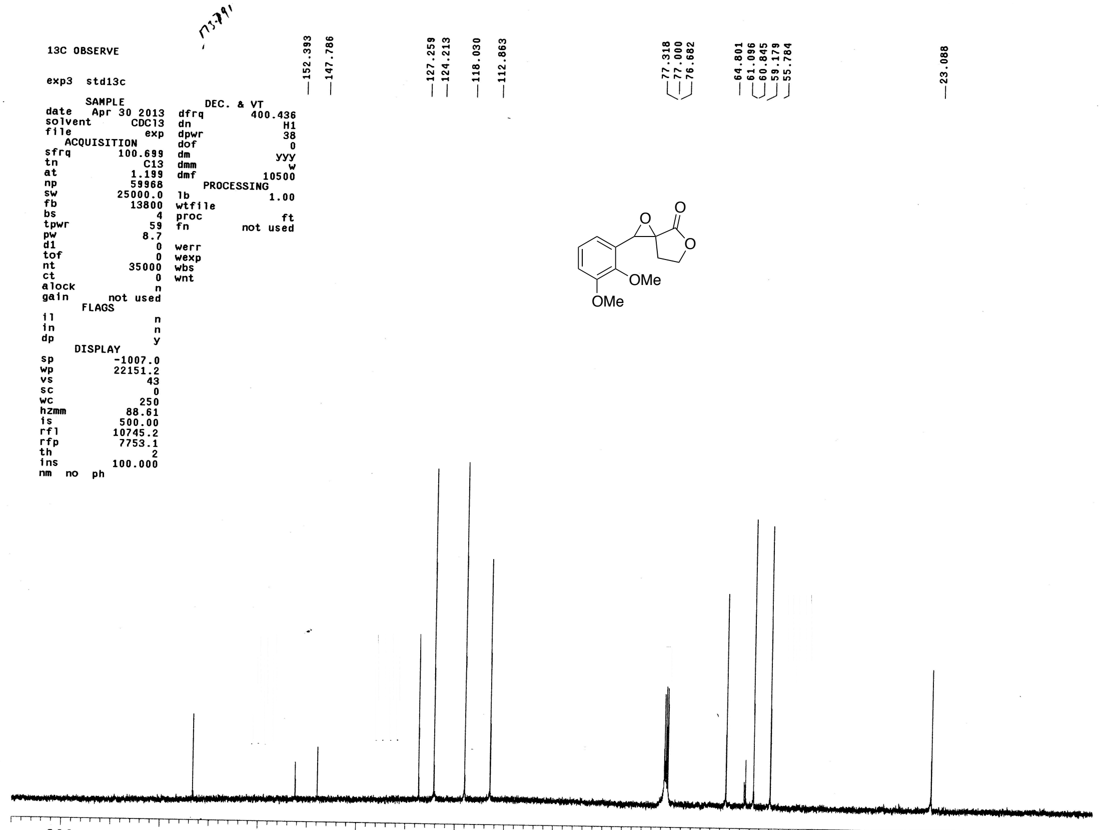

^{13}C NMR spectrum of compound **5n**

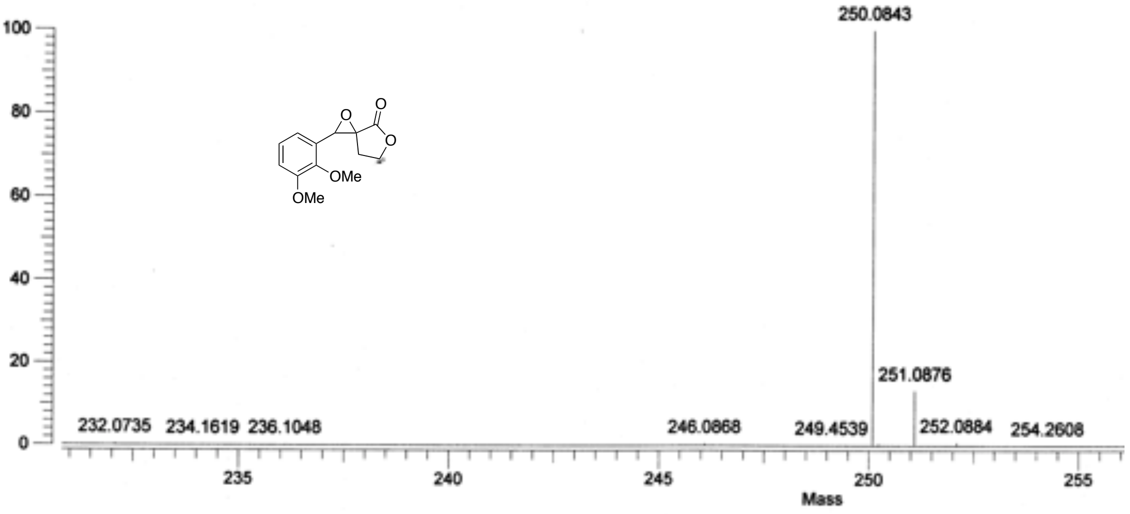

Mass spectrum of compound **5n**

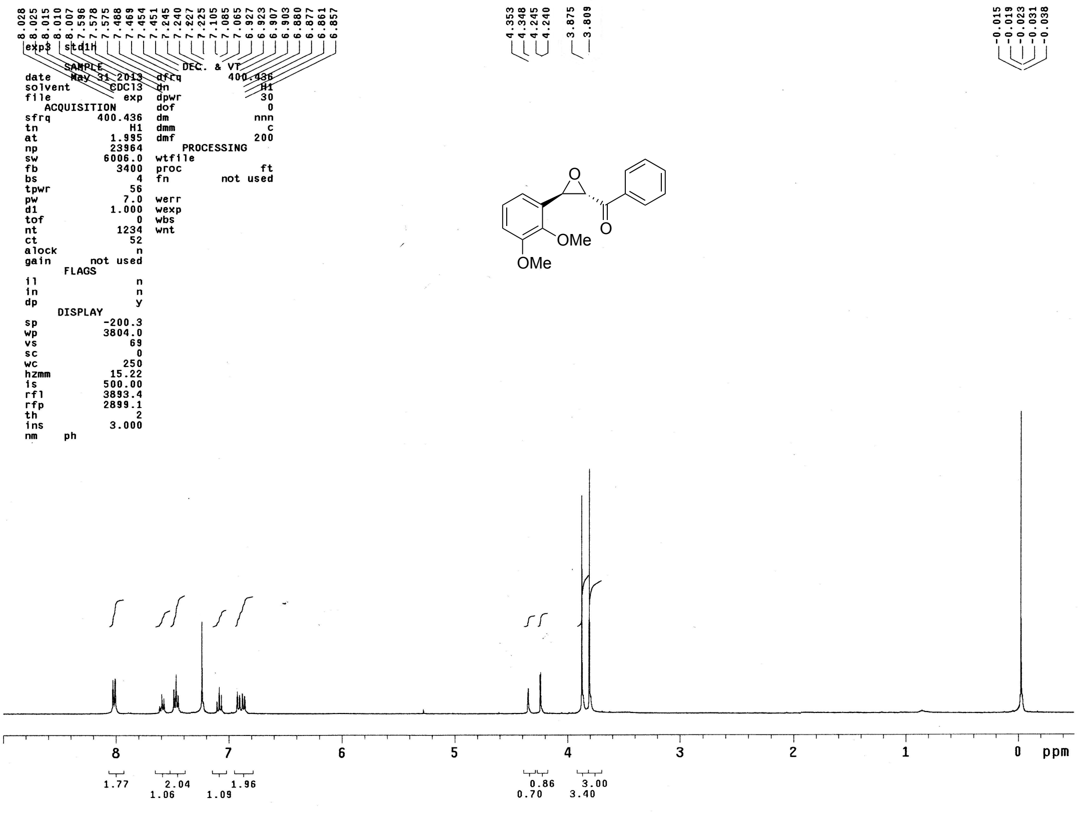

^{1}H NMR spectrum of compound **5o**

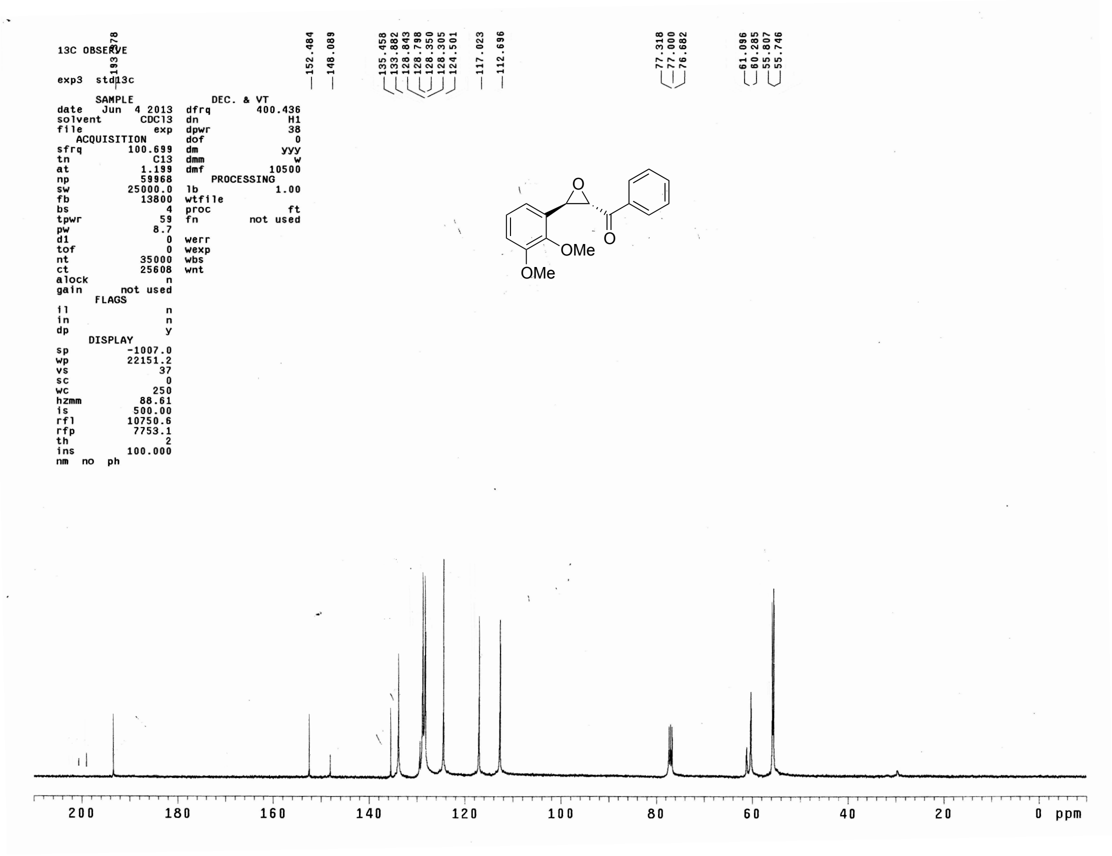

^{13}C NMR spectrum of compound **5o**

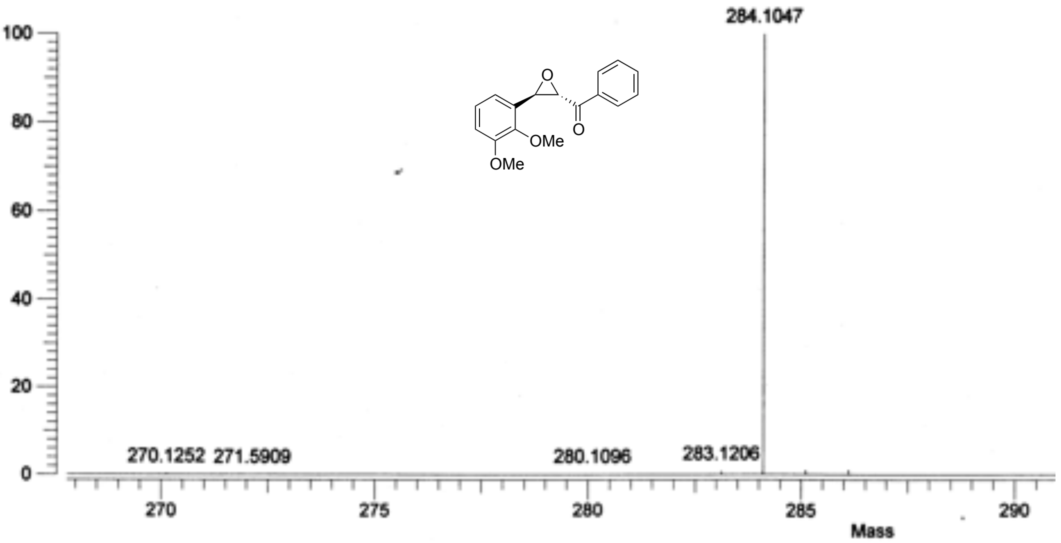

Mass spectrum of compound **5o**

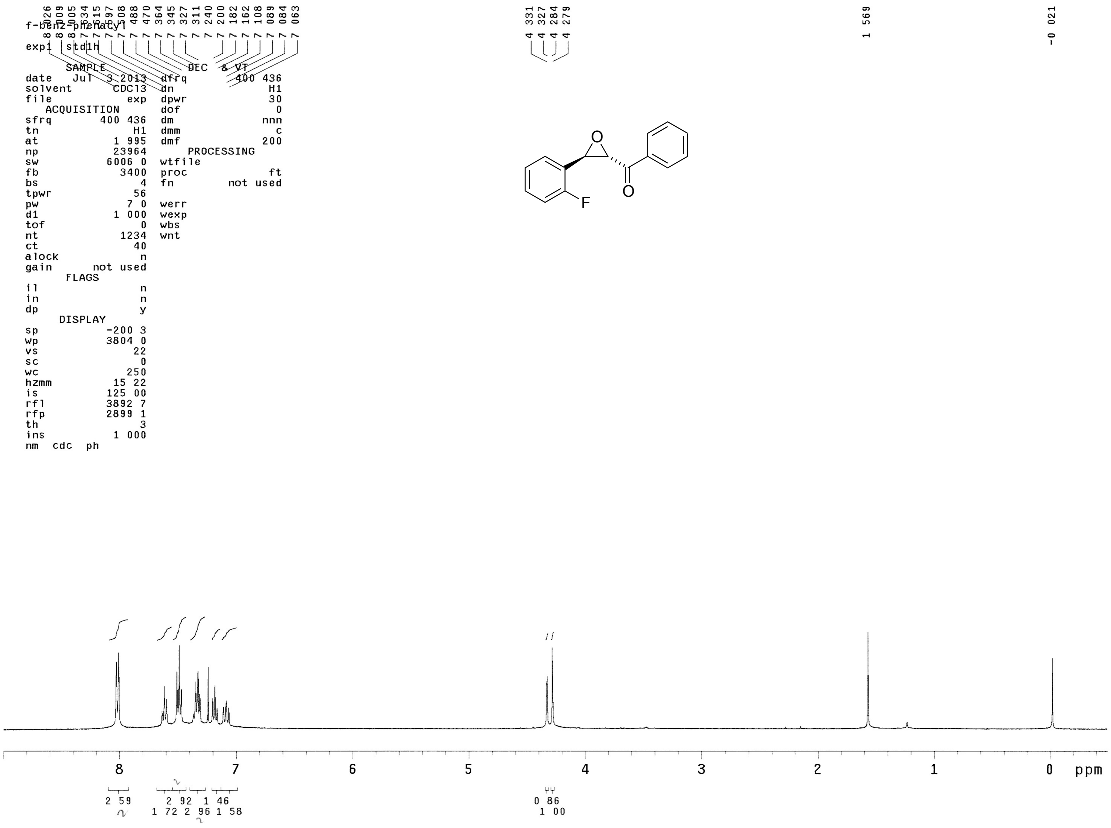

^{1}H NMR spectrum of compound **5p**

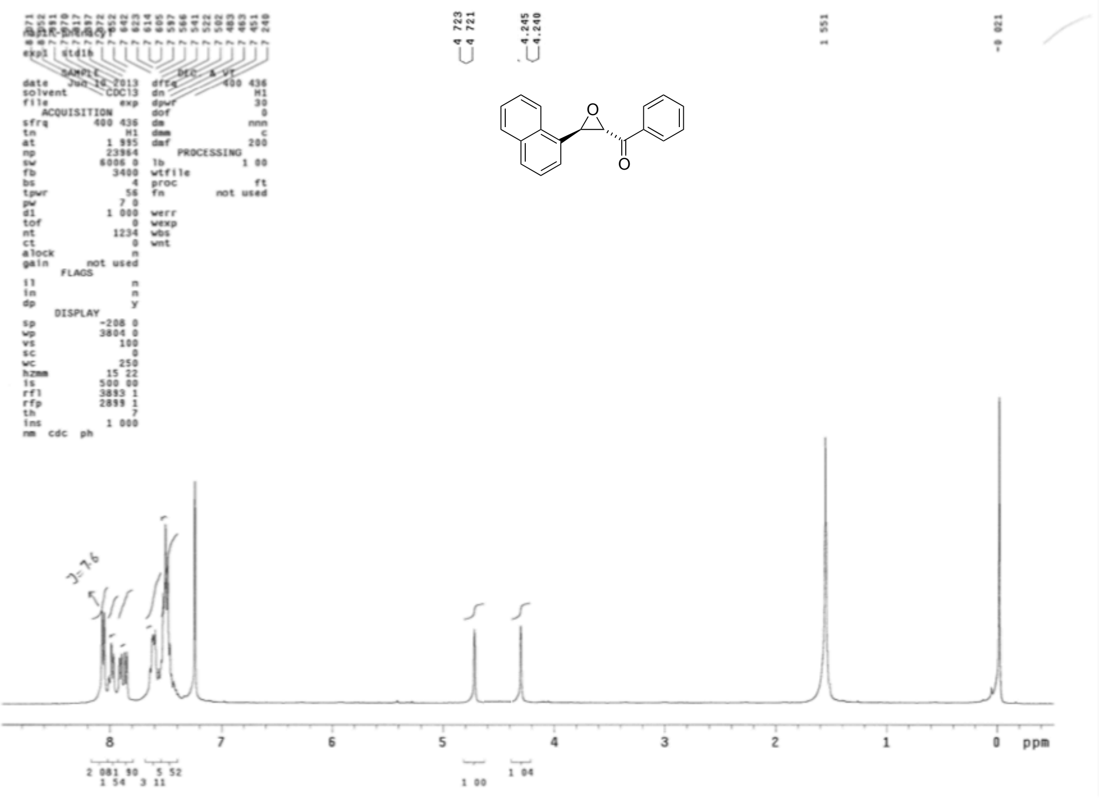

^{1}H NMR spectrum of compound **5q**

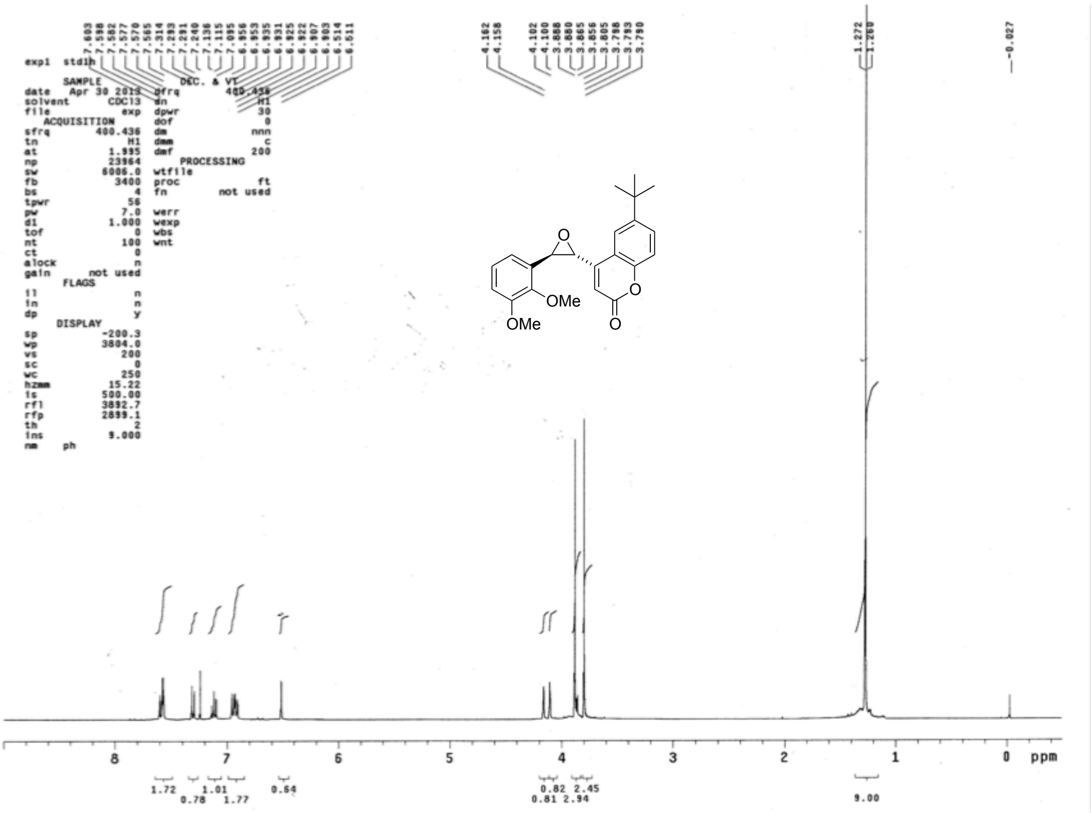

^{1}H NMR spectrum of compound **11a**

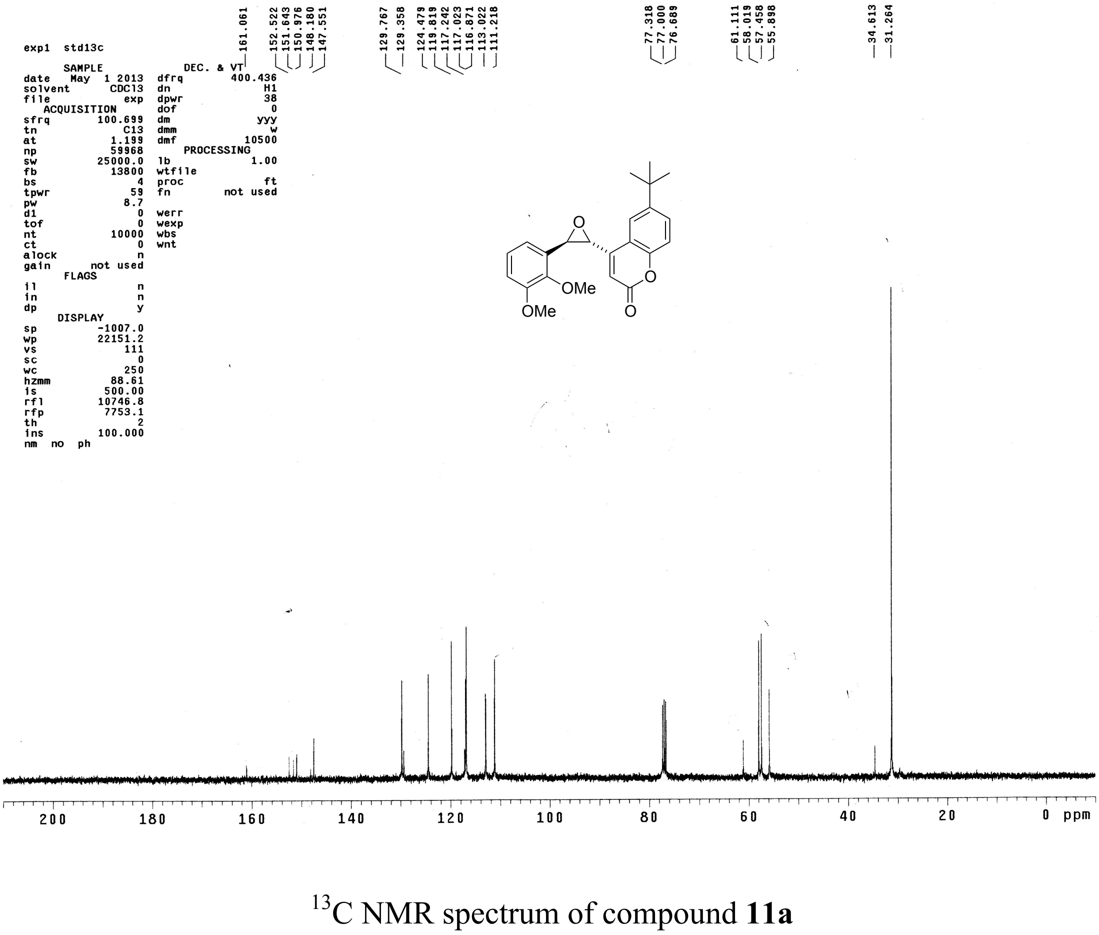

^{13}C NMR spectrum of compound **11a**

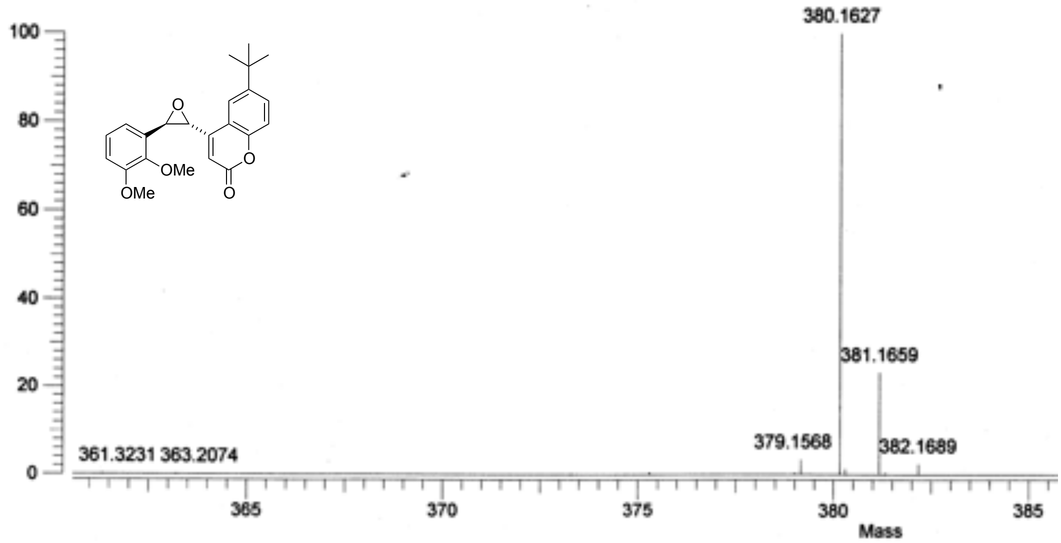

Mass spectrum of compound **11a**

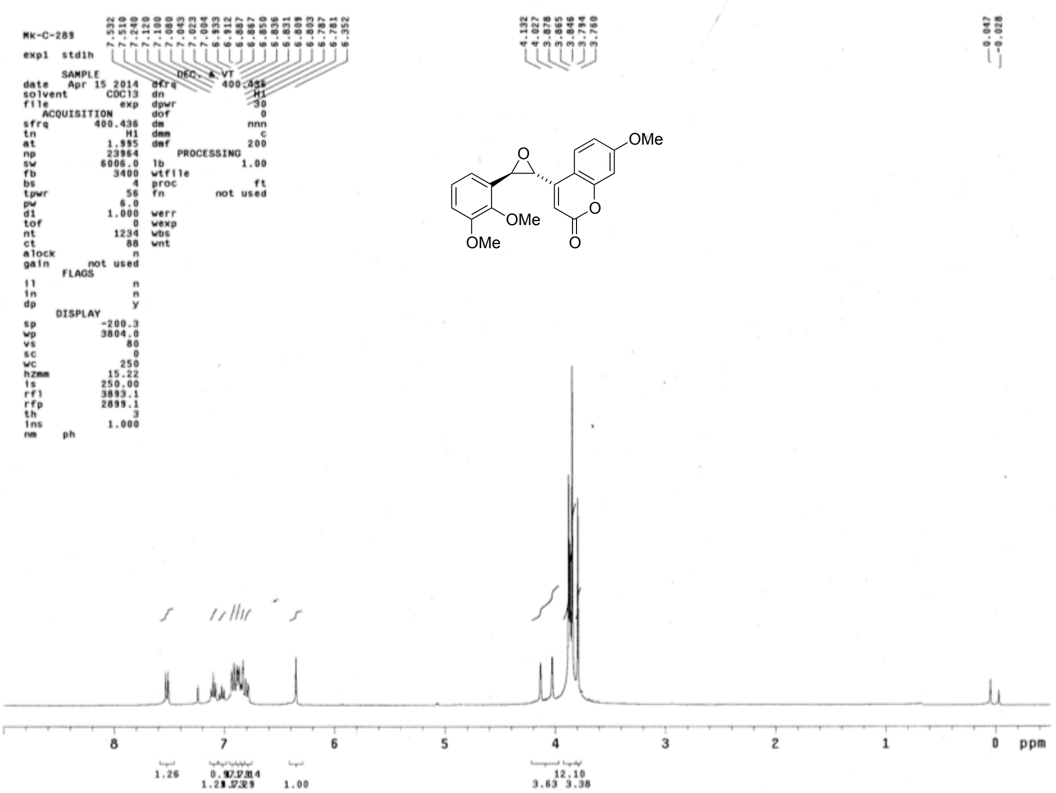

^{1}H NMR spectrum of compound **11b**

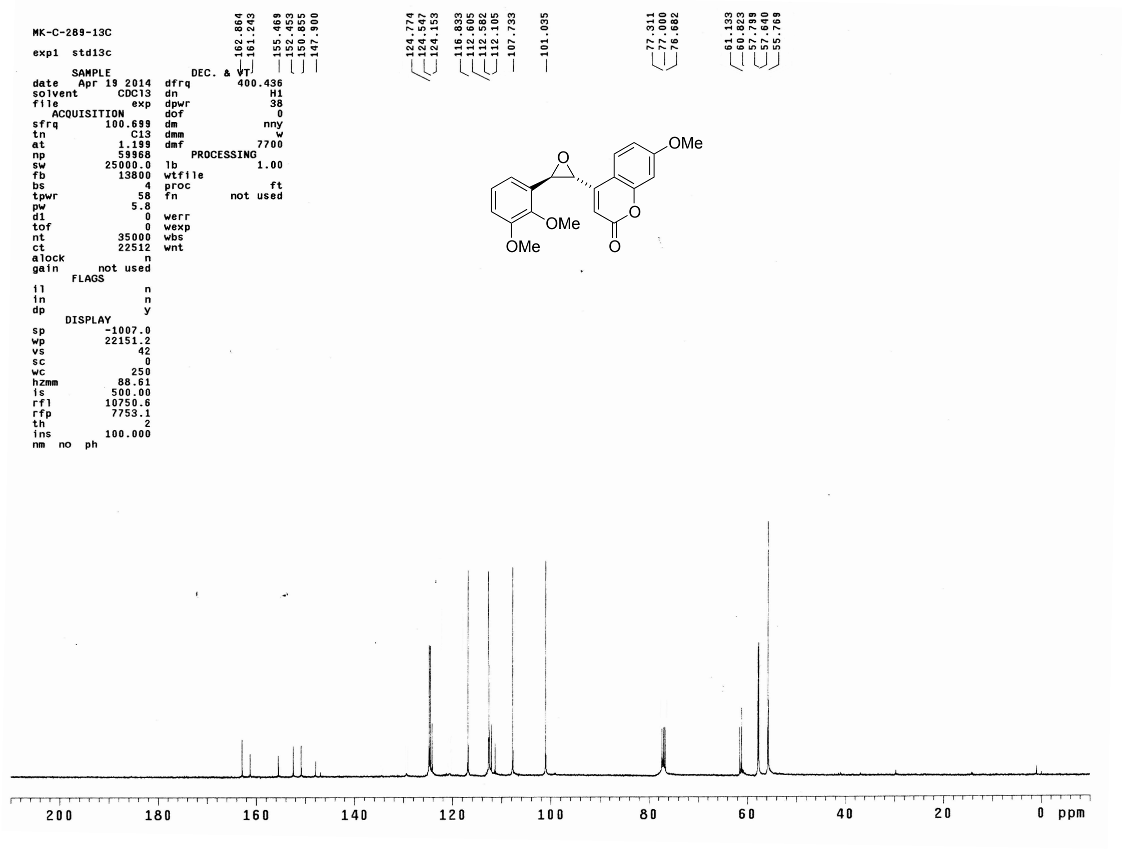

^{13}C NMR spectrum of compound **11b**

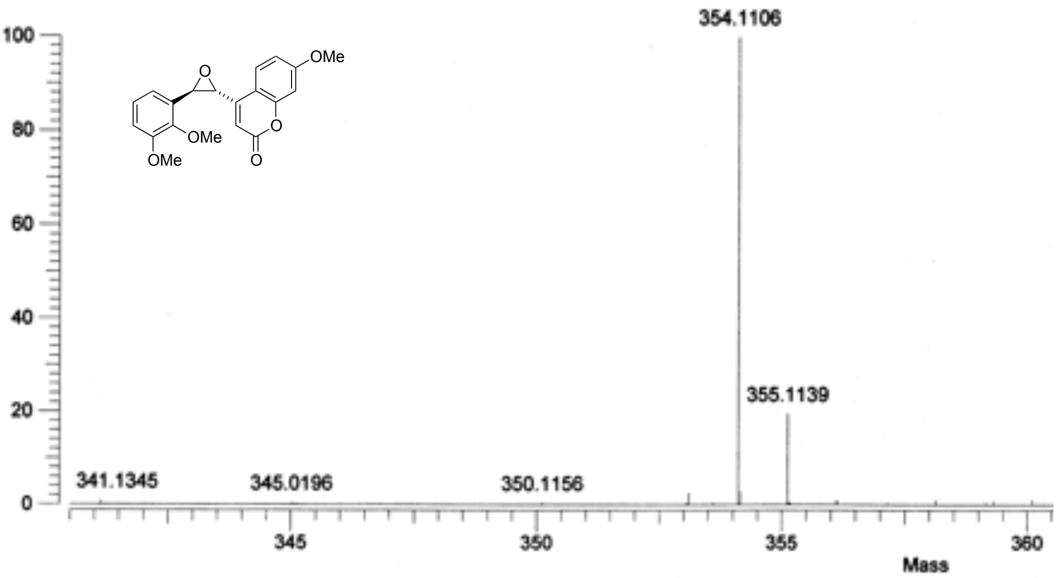

Mass spectrum of compound **11b**

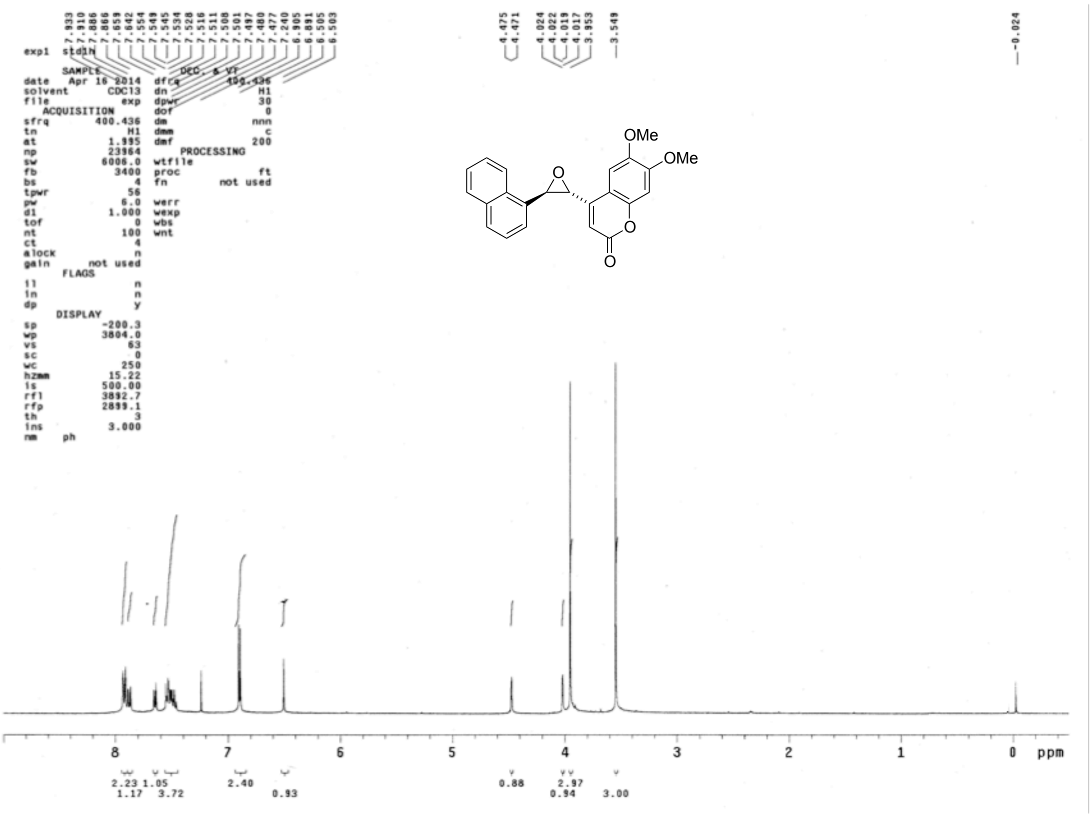

^{1}H NMR spectrum of compound **11c**

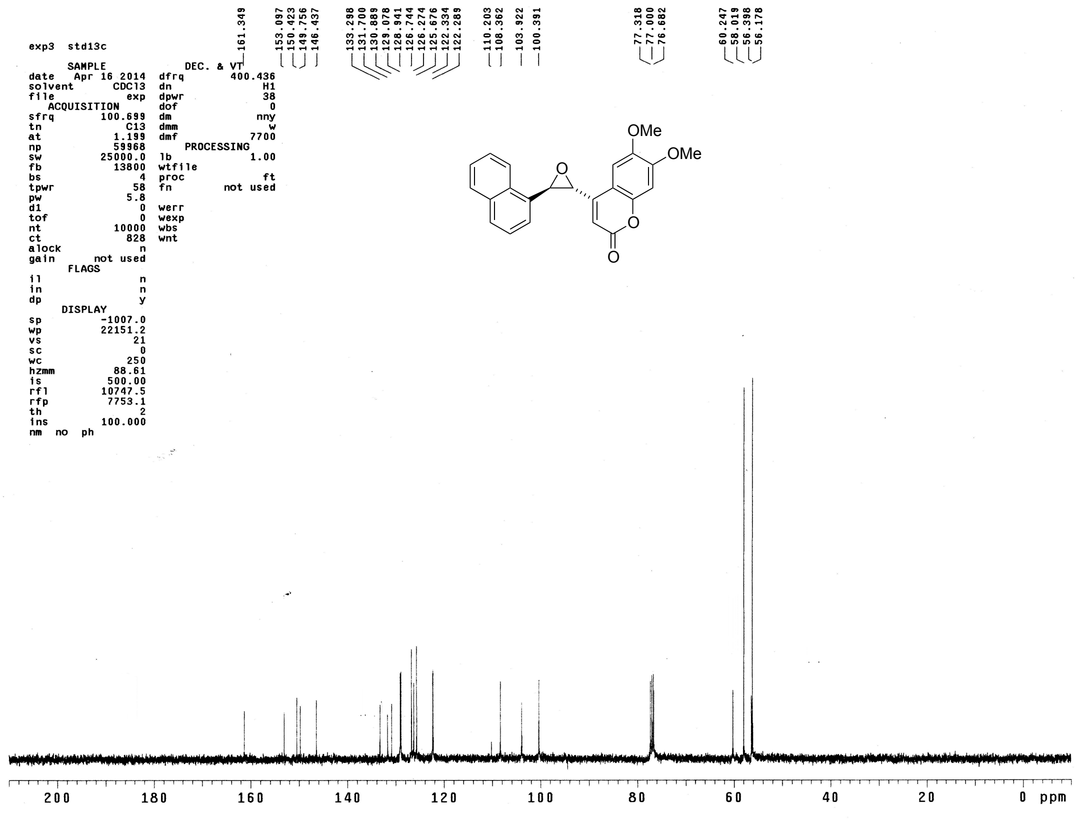

^{13}C NMR spectrum of compound **11c**

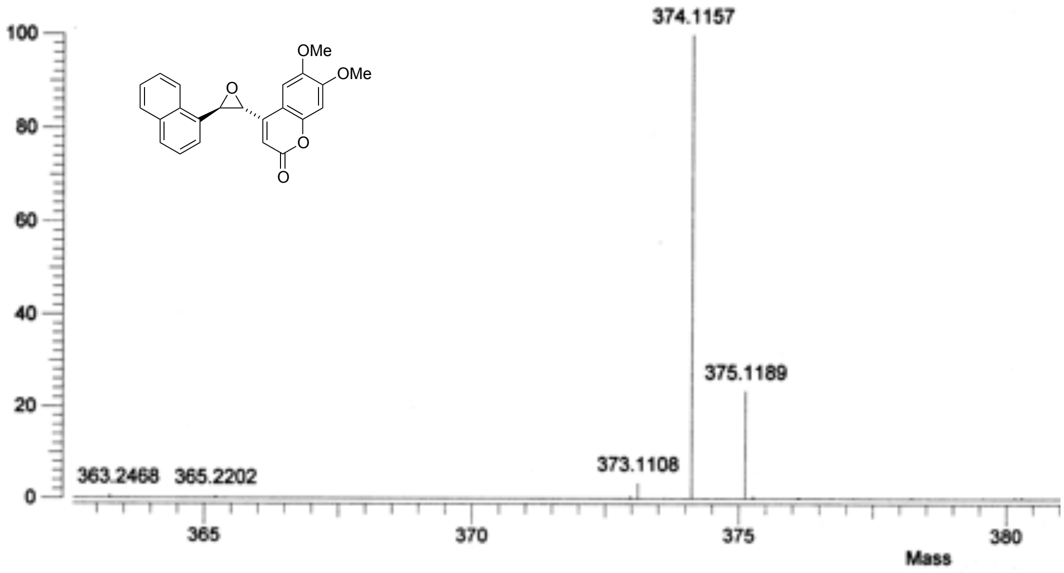

Mass spectrum of compound **11c**
